# Patient education interventions for the management of inflammatory bowel disease

**DOI:** 10.1002/14651858.CD013854.pub2

**Published:** 2023-05-04

**Authors:** Morris Gordon, Vassiliki Sinopoulou, Ummulkhulsum Ibrahim, Mansour Abdulshafea, Kelly Bracewell, Anthony K Akobeng

**Affiliations:** School of MedicineUniversity of Central LancashirePrestonUK; University of Central LancashirePrestonUK; Pediatric GastroenterologySidra MedicineDohaQatar

**Keywords:** Humans, Chronic Disease, Colitis, Ulcerative, Colitis, Ulcerative/therapy, Crohn Disease, Inflammatory Bowel Diseases, Inflammatory Bowel Diseases/therapy, Neoplasm Recurrence, Local, Patient Education as Topic, Quality of Life

## Abstract

**Background:**

Inflammatory bowel disease (IBD) is a life‐long condition for which currently there is no cure. Patient educational interventions deliver structured information to their recipients. Evidence suggests patient education can have positive effects in other chronic diseases.

**Objectives:**

To identify the different types of educational interventions, how they are delivered, and to determine their effectiveness and safety in people with IBD.

**Search methods:**

On 27 November 2022, we searched CENTRAL, Embase, MEDLINE, ClinicalTrials.gov, and WHO ICTRP with no limitations to language, date, document type, or publication status. Any type of formal or informal educational intervention, lasting for any time, that had content focused directly on knowledge about IBD or skills needed for direct management of IBD or its symptoms was included. Delivery methods included face‐to‐face or remote educational sessions, workshops, guided study via the use of printed or online materials, the use of mobile applications, or any other method that delivers information to patients.

**Selection criteria:**

All published, unpublished and ongoing randomised control trials (RCTs) that compare educational interventions targeted at people with IBD to any other type of intervention or no intervention.

**Data collection and analysis:**

Two review authors independently conducted data extraction and risk of bias assessment of the included studies. We analysed data using Review Manager Web. We expressed dichotomous and continuous outcomes as risk ratios (RRs) and mean differences (MDs) with 95% confidence intervals (CIs). We assessed the certainty of the evidence using GRADE methodology.

**Main results:**

We included 14 studies with a total of 2708 randomised participants, aged 11 to 75 years. Two studies examined populations who all had ulcerative colitis (UC); the remaining studies examined a mix of IBD patients (UC and Crohn's disease). Studies considered a range of disease activity states. The length of the interventions ranged from 30 minutes to 12 months. Education was provided in the form of in‐person workshops/lectures, and remotely via printed materials or multimedia, smartphones and internet learning.

Thirteen studies compared patient education interventions plus standard care against standard care alone. The interventions included seminars, information booklets, text messages, e‐learning, a multi professional group‐based programme, guidebooks, a staff‐delivered programme based on an illustrated book, a standardised programme followed by group session, lectures alternating with group therapy, educational sessions based on an IBD guidebook, internet blog access and text messages, a structured education programme, and interactive videos.

Risk of bias findings were concerning in all judgement areas across all studies. No single study was free of unclear or high of bias judgements.

Reporting of most outcomes in a homogeneous fashion was limited, with quality of life at study end reported most commonly in six of the 14 studies which allowed for meta‐analysis, with all other outcomes reported in a more heterogeneous manner that limited wider analysis. Two studies provided data on disease activity. There was no clear difference in disease activity when patient education (n = 277) combined with standard care was compared to standard care (n = 202). Patient education combined with standard care is probably equivalent to standard care in reducing disease activity in patients with IBD (standardised mean difference (SMD) ‐0.03, 95% CI ‐0.25 to 0.20), moderate‐certainty evidence.

Two studies provided continuous data on flare‐up/relapse. There was no clear difference for flare‐ups or relapse when patient education (n = 515) combined with standard care was compared to standard care (n = 507), as a continuous outcome. Patient education combined with standard care is probably equivalent to standard care in reducing flare‐ups or relapse in patients with IBD (MD ‐0.00, 95% CI ‐0.06 to 0.05; moderate‐certainty evidence).

Three studies provided dichotomous data on flare‐up/relapse. The evidence is very uncertain on whether patient education combined with standard care (n = 157) is different to standard care (n = 150) in reducing flare‐ups or relapse in patients with IBD (RR 0.94, 95% CI 0.41 to 2.18; very low‐certainty evidence).

Six studies provided data on quality of life. There was no clear difference in quality of life when patient education combined with standard care (n = 721) was compared to standard care (n = 643). Patient education combined with standard care is probably equivalent to standard care in improving quality of life in patients with IBD (SMD 0.08, 95% CI ‐0.03 to 0.18; moderate‐certainty evidence).

The included studies did not report major differences on healthcare access. Medication adherence, patient knowledge and change in quality of life showed conflicting results that varied between no major differences and differences in favour of the educational interventions.

Only five studies reported on adverse events. Four reported zero total adverse events and one reported one case of breast cancer and two cases of surgery in their interventions groups, and zero adverse events in their control group.

Two studies compared delivery methods of patient education, specifically: web‐based patient education interventions versus colour‐printed books or text messages; and one study compared frequency of patient education, specifically: weekly educational text messages versus once every other week educational text messages. These did not show major differences for disease activity and quality of life.

Other outcomes were not reported.

**Authors' conclusions:**

The ways in which patient educational support surrounding IBD may impact on disease outcomes is complex.

There is evidence that education added to standard care is probably of no benefit to disease activity or quality of life when compared with standard care, and may be of no benefit for occurrence of relapse when compared with standard care. However, as there was a paucity of specific information regarding the components of education or standard care, the utility of these findings is questionable.

Further research on the impact of education on our primary outcomes of disease activity, flare‐ups/relapse and quality of life is probably not indicated. However, further research is necessary, which should focus on reporting details of the educational interventions and study outcomes that educational interventions could be directly targeted to address, such as healthcare access and medication adherence. These should be informed by direct engagement with stakeholders and people affected by Crohn's and colitis.

## Plain language summary

**Education programmes for patients with inflammatory bowel disease (IBD)**

**Key messages**

It is likely that patient education programmes have no additional benefits when compared to usual medications and care for:

• improving inflammatory bowel disease (IBD);

• avoiding relapses and flare‐ups of the disease; or

• improving quality of life for patients with IBD.

**What is inflammatory bowel disease?**

Inflammatory bowel disease refers mainly to two conditions that cause inflammation of the gut. These are ulcerative colitis and Crohn's disease. Ulcerative colitis only affects the large intestine. Crohn's disease can affect any part of the gut, from mouth to bottom.

IBD can mainly cause tummy pain or discomfort, diarrhoea that can be bloody, weight loss, and tiredness.

**How is inflammatory bowel disease treated?**

There is no cure for IBD. Treatment usually involves medications and surgery, but milder cases may not need treatment. Additional treatments can include diets and other lifestyle changes.

**What did we want to find out?**

It is possible that education programmes may benefit people with IBD. The education can be delivered face‐to‐face, with the patient and educator being at the same or different locations, such as in live lectures, seminars and workshops, or at a distance without live communication, such as with the use of the Internet, smartphones, books and videos.

We wanted to find out if education programmes for patients with IBD can have benefits for disease improvement, relapses or flare‐ups, and quality of life. We also wanted to find out about their effects on healthcare access, missing medications, or overall patient knowledge of IBD. Additionally, we wanted to find out how safe the education programmes are, even though safety issues were unlikely.

**What did we do?**

We searched for randomised controlled trials (studies where participants are randomly assigned to one of two or more treatment groups) that compared patient education with any other treatment in people of all ages with IBD.

**What did we find?**

We found 14 trials, with a total of 2708 participants who were aged 11 to 75 years. The education programmes were delivered via the internet, smartphones, books or videos, or through face‐to‐face lectures.

The length of the interventions ranged from a single 30‐minute session to 12 months. Two studies examined populations where all the participants had ulcerative colitis, while the remaining studies examined people with a mix of ulcerative colitis and Crohn's disease. Thirteen of the studies compared patient education that was given alongside standard treatment to standard treatment alone.

Our conclusions were that

• Patient‐education programmes probably have no additional benefits to usual medications and care for:

‐ improving IBD symptoms; ‐ avoiding relapses and flare‐ups; ‐ improving the quality of life of people with IBD.

• We do not know if or how education impacts access to health care, missing medications, or overall patient knowledge of IBD, as these were not reported in a way that allowed us to make conclusions.

• The safety of the education programmes was not well‐reported, possibly because education programmes are unlikely to have any safety dangers.

One of the studies compared education given through the internet to education give through books, and another compared educational text message sent once every other week to texts sent weekly. The evidence for these comparisons was limited, and we could not reach meaningful conclusions.

**What next?**

Further research on patient education should focus on details within the education programmes and examine different targets, such as how education can help reduce missing medication and the best ways to access health care.

**What are the limitations of the evidence?**

One limitation of the evidence was that the educational programmes were not very well described. A lot of the studies were unclear about what their education programme aimed to achieve, how, and the resources needed. Another limitation is that some items the studies measured, such as disease improvement or flare‐ups might not have been the best targets for educational programmes. Others such as health care, missing medications and patient knowledge may be better, but they were measured in a variety of ways that did not allow us to combine them. Also, standard care, to which patient education programmes were added and compared, was not described in great detail. This means that standard care might vary from one study to another, which could make our findings less accurate. Finally, some of the research methods that the studies used were not of the best quality.

**How up‐to‐date is this review?**

This review is up‐to‐date as of 27 November 2022.

## Summary of findings

**Summary of findings 1:** Patient education and standard care compared to standard care for the management of inflammatory bowel disease

**Patient education and standard care compared to standard care for the management of inflammatory bowel disease**						
**Patient or population:** people with inflammatory bowel disease **Setting:** hospitals and tertiary centres in USA, Canada, Germany, Sweden, UK, the Netherlands **Intervention:** patient education plus standard care (the patient education interventions were information booklets, text messages, e‐learning, a multi professional group‐based programme, guidebooks, a staff‐delivered programme based on an illustrated book, a standardised programme followed by group session, lectures alternating with group therapy, educational sessions based on an inflammatory bowel disease guidebook, internet blog access and text messages, a structured education programme, and interactive videos) **Comparison:** standard care						
**Outcomes**	Anticipated absolute effects^*^ (95% CI)		**Relative effect (95% CI)**	**№ of participants (studies)**	**Certainty of the evidence (GRADE)**	**Comments**
	**Risk with standard care**	**Risk with patient education and standard care**				
Disease activity (3‐12 months)	‐	SMD^a^ 0.03 lower (0.25 lower to 0.2 higher)	‐	479 (2 studies)	⊕⊕⊕⊝ moderate ^b^	As a rule of thumb (i.e. a broadly accurate guide), 0.2 SMD represents a small difference, 0.5 SMD a moderate one, and 0.8 SMD a large effect.
Flare‐ups or relapse (mean number during study period, start‐12 months) (continuous outcome)	‐	MD 0.00 lower (0.06 lower to 0.05 higher)	‐	1022 (2 studies)	⊕⊕⊕⊝ moderate ^b^	‐
Flare‐ups or relapse (4‐12 months) (dichotomous outcome)	Study population		RR 0.94 (0.41 to 2.18)	307 (3 studies)	⊕⊝⊝⊝ very low^c^	‐
	67 per 1000	63 per 1000 (27 to 188)				
Quality of life (2 weeks‐12 months)	‐	SMD^a^ 0.08 higher (0.03 lower to 0.18 higher)	‐	1364 (6 studies)	⊕⊕⊕⊝ moderate ^b^	As a rule of thumb, 0.2 SMD represents a small difference, 0.5 SMD a moderate one, and 0.8 SMD a large effect.
*The risk in the intervention group (and its 95% confidence interval) is based on the assumed risk in the comparison group and the **relative effect** of the intervention (and its 95% CI). **CI:** confidence interval; **RR:** risk ratio; **SMD:** standardised mean difference						
**GRADE Working Group grades of evidence** **High certainty:** we are very confident that the true effect lies close to that of the estimate of the effect **Moderate certainty:** we are moderately confident in the effect estimate: the true effect is likely to be close to the estimate of the effect, but there is a possibility that it is substantially different **Low certainty:** our confidence in the effect estimate is limited: the true effect may be substantially different from the estimate of the effect **Very low certainty:** we have very little confidence in the effect estimate: the true effect is likely to be substantially different from the estimate of effect						

^a^ SMD was used when a continuous outcome was measured on two or more different scales by the studies included in the meta‐analysis ^b^Downgraded one level due to concerns with risk of bias, related mainly to blinding and allocation concealment ^c^Downgraded three levels: one level due to serious concerns with risk of bias, related mainly to blinding and allocation concealment, and two levels due to imprecision due to very low event numbers.

**Summary of findings 2:** Web‐based patient education versus other delivery of patient education for the management of inflammatory bowel disease

**Web‐based patient education versus other delivery of patient education for the management of inflammatory bowel disease**				
**Patient or population:** people with inflammatory bowel disease **Setting:** hospitals and tertiary centres in USA and Turkey **Intervention:** web‐based education **Comparison:** educational information via easy‐to‐read, illustrated, colour‐printed books				
**Outcomes**	**Impacts**	**№ of participants (studies)**	**Certainty of the evidence (GRADE)**	**Comments**
Disease activity (8 weeks)	UC participants: 8/16 in the web‐based group and 10/16 in the control education group were in remission; 6/16 and 4/16 had mild disease; 2/16 and 1/16 had severe disease; and 0/16 and 0/16 had very severe disease. CD participants: 5/14 in the web‐based group and 10/14 in the control education group were in remission; 7/14 and 3/14 had mild disease; 2/14 and 1/14 had severe disease; and 0/14 and 0/14 had very severe disease.	1 study (32 UC participants and 26 CD participants)	⊕⊝⊝⊝ Very low^a^	
Flare‐ups or relapse (continuous)	‐	‐	‐	‐
Flare‐ups or relapse (dichotomous)	‐	‐	‐	‐
Quality of life, IBDQ (32 minimum score to 224 maximum score; high score = better quality of life) (8 weeks)	Mean (SD) quality of life scores: Web‐based group 156.53 (30.97) Control group 155.63 (34.30)	1 study (58 participants)	⊕⊝⊝⊝ Very low^a^	
*The risk in the intervention group (and its 95% confidence interval) is based on the assumed risk in the comparison group and the **relative effect** of the intervention (and its 95% CI). **CD:** Crohn's Disease; **IBDQ:** Inflammatory Bowel Disease Questionnaire; **SD:** standard deviation; **UC:** ulcerative colitis				
**GRADE Working Group grades of evidence** **High certainty:** we are very confident that the true effect lies close to that of the estimate of the effect. **Moderate certainty:** we are moderately confident in the effect estimate: the true effect is likely to be close to the estimate of the effect, but there is a possibility that it is substantially different. **Low certainty:** our confidence in the effect estimate is limited: the true effect may be substantially different from the estimate of the effect. **Very low certainty:** we have very little confidence in the effect estimate: the true effect is likely to be substantially different from the estimate of effect.				

^a^ Downgraded three levels: two levels for serious imprecision due to very low participant and event numbers, and one level due to serious concerns with risk of bias for randomisation, allocation concealment, blinding and attrition.

**Summary of findings 3:** Weekly educational texts messages versus once every other week educational text messages for the management of inflammatory bowel disease

**Weekly educational texts messages versus once every other week educational text messages for the management of inflammatory bowel disease**				
**Patient or population:** people with inflammatory bowel disease **Setting:** hospital in USA **Intervention:** every other week educational text messages **Comparison:** weekly educational text messages				
**Outcomes**	**Impact**	**№ of participants (studies)**	**Certainty of the evidence (GRADE)**	**Comments**
Disease activity (12 months)	UC participants (SCCAI score, minimum 0, maximum 19; low score = better result): Mean (SD) disease activity for the every other week UC participants was 1.7 (1.9) Mean (SD) disease activity for the weekly UC participants was 2.0 (1.8). CD participants (HBI score, minimum 0, maximum 18; low score = better result): Mean (SD) disease activity for the every other week CD participants was 4.2 (3.9) Mean (SD) disease activity for the weekly CD participants 3.2 (3.4).	1 study (131 CD and 62 UC participants)	⊕⊝⊝⊝ Very low^a^	‐
Flare‐ups or relapse (continuous)	‐	‐	‐	‐
Flare‐ups or relapse (dichotomous)	‐	‐	‐	‐
Quality of life, IBDQ (32 minimum score ‐ 224 maximum score; high score = better quality of life) (12 months)	Mean (SD) quality of life scores for the every other week participants was 181.5 (28.2) and for the weekly participants was 179.2 (32.8)	1 study (193 participants)	⊕⊝⊝⊝ Very low^a^	‐
*The risk in the intervention group (and its 95% confidence interval) is based on the assumed risk in the comparison group and the **relative effect** of the intervention (and its 95% CI). **CD:** Crohn's disease; **HBI:** Harvey‐Bradshaw Index for Crohn's Disease; **IBDQ:** Inflammatory Bowel Disease Questionnaire; **SCCAI:** Simple Clinical Colitis Activity Index; **SD:** standard deviation; **UC:** ulcerative colitis				
**GRADE Working Group grades of evidence** **High certainty:** we are very confident that the true effect lies close to that of the estimate of the effect. **Moderate certainty:** we are moderately confident in the effect estimate: the true effect is likely to be close to the estimate of the effect, but there is a possibility that it is substantially different. **Low certainty:** our confidence in the effect estimate is limited: the true effect may be substantially different from the estimate of the effect. **Very low certainty:** we have very little confidence in the effect estimate: the true effect is likely to be substantially different from the estimate of effect.				

^a^Downgraded three levels: one level due to concerns with risk of bias due to blinding, and two levels due to serious concerns with imprecision due to very low participant numbers

## Background

### Description of the condition

Inflammatory bowel disease (IBD) is an umbrella term for a range of conditions that cause inflammation to the human gastrointestinal tract, with the most prominent ones being ulcerative colitis (UC) and Crohn's disease. Symptoms can include pain, cramping, swelling, diarrhoea, weight loss and tiredness. The aetiology of IBD is still undetermined, but it is thought to be caused via a complex interaction of genetic and environmental factors (De Souza 2017). More specifically, it is thought that IBD is due to an aberrant immune response to the gut commensal flora in a genetically susceptible individual (Pizarro 2019). IBD is a life‐long condition for which currently there is no cure. Treatment options include medications, lifestyle and diet changes, and surgery with the aim of inducing and maintaining remission of the disease. It is estimated that more than 6.8 million people are living with IBD globally, with incidences of the disease rising especially in regions that are newly adopting western lifestyles (Jairath 2020; Kaplan 2017). Apart from its physical manifestations, IBD can have a serious impact on patients' psychological and social well‐being by limiting the patient's ability to take part in social activities and engagements. It also places a significant burden on healthcare systems, with an estimated EUR 4.6 billion to EUR 5.6 billion of annual healthcare costs attributed to IBD in Europe and USD 7.2 billion in the USA (Burisch 2013; Windsor 2019).

### Description of the intervention

Patient educational interventions aim to deliver structured information to the recipient of the intervention and there is evidence to suggest patient education can have positive effects in other chronic diseases on specific clinical and quality of life outcomes (Anderson 2017; Howcroft 2016; Rush 2018). However, the content, delivery method, duration and specific purposes of any given intervention can vary considerably and there are no set standards for any of these parameters.

Local resources and healthcare systems, as well as individual patient factors, can have a major impact on patient education. Therefore, there is a need to understand whether such interventions can affect patient outcomes, and how and why they affect patient outcomes.

### How the intervention might work

Education will enhance patient knowledge surrounding IBD. However, the question of how this may impact on their disease outcomes is complex. One point of focus has been about advising patients how to determine when their disease is deteriorating, so they can contact their healthcare provider. Improving medication adherence, recognising adverse effects and when to report them, and improving compliance might be some ways patient education interventions might work.

IBD can affect patients' daily lives in several ways and can lead to a lower health‐related quality of life (HRQoL). Together with provider‐led management, self‐management and knowledge about their disease can play an important role in giving patients control over their condition. IBD educational interventions can provide patients with important information and advice towards that end.

### Why it is important to do this review

More clarity about the types of educational interventions targeting people with IBD that have been researched at a randomised controlled trial (RCT) level; what they entail and to what extent they are effective is vital for people with IBD to make better informed decisions for the self‐management of their condition.

It is important to review the evidence that has sought to address deficits identified in education systematically (NRAD 2015), and to assess the attributes of training packages, so they can be applied effectively (Norcini 2011). The extent to which we can answer 'how' training can be designed, 'why' it is effective and 'for whom and when' will depend on descriptive data within primary studies, but it is important to highlight this information to help professionals understand and deliver health education in a reliable and reproducible manner (Gordon 2011; Gordon 2013).

## Objectives

To identify the different types of educational interventions, how they are delivered, and to determine their effectiveness and safety in people with inflammatory bowel disease (IBD).

## Methods

### Criteria for considering studies for this review

#### Types of studies

All published, unpublished and ongoing RCTs that compare educational interventions targeted at people with IBD to any other type of intervention or no intervention.

Cluster‐randomised and cross‐over trials that met our criteria were included.

#### Types of participants

People with IBD of all ages.

#### Types of interventions

Any type of formal or informal educational intervention, lasting for any time, that has content focused directly on knowledge about IBD or skills needed for direct management of IBD or its symptoms. Interventions that use education to deliver a different set of skills or outcomes that may by proxy enhance patients outcomes were not included (e.g. cognitive behavioural therapy (CBT) training, hypnotherapy training, relaxation therapy training, training on how to use a remote or other health tool for monitoring disease, training on diagnostic tools).

Delivery methods can include face‐to‐face or remote educational sessions or workshops, guided study via the use of printed or online materials, the use of mobile applications or any other method that delivers information to patients.

It became clear through data extraction that many papers did not mention details about standard therapies. Our team discussed this, and decided that it was highly unlikely that patients would be denied treatment in lieu of patient education or the control therapies. In addition, we could not assume the use of placebo if it was not mentioned by the authors. We considered terms such as “standard care”, “usual care”, “treatment as usual”, “routine follow‐up”, as interchangeable. We recognise this is a source of clinical heterogeneity, as these terms can refer to different approaches of standard care which are not identical in every way, however, we agreed they were probably similar enough for the meta‐analysis purposes of this review.

We have listed all intervention and comparator groups in the 'Characteristics of included studies' table.

#### Types of outcome measures

We considered both dichotomous and continuous outcomes for this review. These were not used as criteria for considering inclusion.

##### Primary outcomes

- Disease activity at study end, using a recognised disease activity scoring system as described by the study authors.
- Flare‐ups or relapse measured clinically, endoscopically or histologically, during the study period.
- Quality of life at study end using validated scales or tools.

##### Secondary outcomes

- Number of episodes of accessing health care (outpatient, remote or inpatient) during the study follow‐up.
- Change in disease activity using a recognised score at study end.
- Change in quality of life using a validated tool at study end.
- Medication adherence.
- Patient knowledge or skill (or both) as measured by a study, at study end.

###### Adverse effects

- Total adverse effects (serious and minor) at study end (e.g. functional bowel symptoms, worsening disease state symptoms, hospitalisation).
- Adverse events leading to withdrawal during the study (as per examples above).

### Search methods for identification of studies

#### Electronic searches

On 27 November 2022, the information Specialist searched the following sources:

- Cochrane Central Register of Controlled Trials (CENTRAL via Cochrane Library, from inception to issue 11, November 2022) (Appendix 1);
- MEDLINE (via Ovid SP, 1946 to 27 November 2022) (Appendix 2);
- Embase (via Ovid SP, 1974 to 27 November 2022) (Appendix 3);
- ClinicalTrials.gov (www.clinicaltrials.gov; Appendix 4);
- World Health Organization International Clinical Trials Registry Platform (ICTRP, www.who.int/trialsearch/, Appendix 5).

We followed the latest guidelines from Cochrane in designing and running the searches (Lefebvre 2019). We also used the Cochrane highly sensitive search strategy for identifying randomised trials in MEDLINE (sensitivity‐maximising version, 2008 revision, Ovid format) and Cochrane's RCT search filter for Embase (Glanville 2019) for identifying the randomised controlled trials. The MEDLINE search strategy was adapted and translated into the syntax of other sources. We did not apply any date, language, document type, or publication status limitations to this search.

#### Searching other resources

As complementary search methods, we carefully checked relevant systematic reviews for studies for potential inclusion in our review. In addition, we scrutinised the references of included studies in our review. We sought unpublished trials by contacting experts in the field.

We attempted to obtain translations of papers when necessary. If this was needed, translation was completed first and then the study managed for screening and extraction as other papers.

### Data collection and analysis

We carried out data collection and analysis according to the methods recommended in the *Cochrane Handbook for Systematic Reviews of Interventions* (Higgins 2020).

#### Selection of studies

Two review authors (UI and MA) independently screened the titles and abstracts identified from the literature search. We discarded studies that did not meet the inclusion criteria. We then obtained the full report of studies that appeared to meet our inclusion criteria, or for which there was insufficient information to make a final decision. Two review authors independently assessed the reports to establish whether the studies met the inclusion criteria. We resolved disagreements by discussion, and consulted a third review author if resolution was not possible. We entered studies rejected at this or subsequent stages in the 'Characteristics of excluded studies' tables and recorded the main reason for exclusion. We recorded the selection process in sufficient detail to complete a PRISMA flow diagram.

Where studies had multiple publications, we identified and excluded duplicates, and collated the reports of the same study so that each study, rather than each report, is the unit of interest for the review, and such studies have a single identifier with multiple references.

#### Data extraction and management

Two review authors independently carried out data extraction using piloted data extraction forms. We extracted relevant data from full‐text articles that met the inclusion criteria including:

- trial setting: country and number of trial centres;
- methods: study design, total study duration and date;
- participant characteristics: age, socio‐demographics, ethnicity, diagnostic criteria and total number;
- eligibility criteria: inclusion and exclusion criteria;
- intervention and comparator — this included description of the learning outcomes planned for the intervention by the teacher or designer, methods of education used, target audience and any resources required;
- patient outcomes: patient outcome definition, unit of measurement and time of collection;
- outcomes from education: educational outcomes, if described, reported and classified as either satisfaction/reaction, attitudes or knowledge and skills;
- results: number of participants allocated to each group, missing participants, sample size;
- funding source.

#### Assessment of risk of bias in included studies

During data extraction, two review authors independently assessed all studies that met the inclusion criteria for their risk of bias using criteria outlined in the *Cochrane Handbook for Systematic Reviews of Interventions* (Higgins 2011). The domains that we assessed are as follows.

- Sequence generation (selection bias).
- Allocation concealment (selection bias).
- Blinding of participants and personnel (performance bias).
- Blinding of outcome assessment (detection bias).
- Incomplete outcome data (attrition bias).
- Selective reporting (reporting bias).
- Other bias.

We judged the studies to be at low, high or unclear risk of bias for each domain assessed, based on guidance in the *Cochrane Handbook for Systematic Reviews of Interventions* (Higgins 2011).

After data extraction, two review authors compared the extracted data to discuss and resolve discrepancies before the data were transferred into the 'Characteristics of included studies' table. For cluster‐RCTs, we judged risk of bias as prescribed in section 16.3.2 “Assessing risk of bias in cluster‐randomized trials” of the *Cochrane Handbook for Systematic Reviews of Interventions* (Higgins 2011).

#### Measures of treatment effect

For dichotomous outcomes, we expressed treatment effect as risk ratios (RRs) with corresponding 95% confidence intervals (CIs). For continuous outcomes, we expressed the treatment effect as mean difference (MD) with 95% CI if studies used the same scales and methods. However, if studies assessed the same continuous outcome using different methods, we estimated the treatment effect using the standardised mean difference (SMD) with 95% CIs. SMD was used when a continuous outcome was measured on two or more different scales by the studies included in the meta‐analysis. We presented SMDs as standard deviation (SD) units and interpreted them as follows: 0.2 represents a small effect, 0.5 a moderate effect and 0.8 a large effect.

#### Unit of analysis issues

The participant is the unit of analysis. For studies comparing more than two intervention groups, we made multiple pair‐wise comparisons between all possible pairs of intervention groups. To avoid double counting, we divided out shared intervention groups evenly among the comparisons. For dichotomous outcomes, we divided up both the number of events and the total number of participants. For continuous outcomes, we divided up the total number of participants and left the means and standard deviations unchanged (this occurred for Cross 2019). We included cross‐over studies if data were reported separately before and after cross over and we only used data from the first phase for our analysis. In the case of cluster RCTs, we used study data only if the authors used appropriate statistical methods that took the clustering effect into account. We also excluded cluster‐RCTs from a sensitivity analysis to assess their impact on the results.

If studies reported dichotomous event data per episode instead of per patient, given the risk of unit of analysis issues, we contacted the authors for further data. If papers reported outcomes at several time points, we used the longest follow‐up.

#### Dealing with missing data

We contacted authors where there were missing data or where studies had not reported data in sufficient detail. We attempted to estimate missing standard deviations using relevant statistical tools and calculators when studies reported standard errors. We judged studies that failed to report measures of variance as being at high risk of selective reporting bias.

For negative outcomes we used the plausible worst‐case scenario and added the numbers of dropouts to the numerator, as is normal practice for reviews for IBD given the chronic nature of the condition and the high rates of adverse events and treatment failures across a patient's journey. For withdrawals that were specifically due to adverse events, we considered all unspecified reasons and all reasons that did not automatically preclude the possibility of an adverse event, as adverse events. For analyses using continuous outcomes, we used the sample numbers as reported by the authors for each particular continuous outcome. If the sample numbers were not reported, we estimated the sample number based on the attrition percentages reported. For cluster‐trial data we estimated effective sample sizes based on Chapter 23 of the *Cochrane Handbook for Systematic Reviews of Interventions* (Higgins 2020).

#### Assessment of heterogeneity

We scrutinised studies to ensure that they were clinically homogeneous in terms of participants, interventions, comparators and outcomes. To test for statistical heterogeneity, we used a Chi^2^ test. A P value of less than 0.1 gives an indication of the presence of heterogeneity. Inconsistency was quantified and represented by the I^2^ statistic. We interpreted the thresholds as follows (Higgins 2020):

- 0% to 40%: might not be important;
- 30% to 60%: may represent moderate heterogeneity;
- 50% to 90%; may represent substantial heterogeneity;
- 75% to 100%: considerable heterogeneity.

#### Assessment of reporting biases

Most reporting biases were minimised by using an inclusive search strategy. We intended to investigate publication bias using a funnel plot if there were 10 or more studies that contributed to a meta‐analysis. We would determine the magnitude of publication bias by visual inspection of the asymmetry of the funnel plot. In addition, we would test funnel plot asymmetry by performing a linear regression of intervention effect estimate against its standard error, weighted by the inverse of the variance of the intervention effect estimate (Egger 1997).

#### Data synthesis

To summarise the study characteristics, we conducted a narrative synthesis of all the included studies. We then carried out a meta‐analysis if there were two or more studies that assessed similar populations, interventions and outcomes. We synthesised data using the random‐effects model in RevMan Web (RevMan Web 2022). We combined effect estimates of studies which report data in a similar way, in the meta‐analysis. We pooled RRs for dichotomous outcomes and MDs or SMDs for continuous outcomes with 95% CIs. Where we were unable to carry out a meta‐analysis (e.g. due to lack of uniformity in data reporting), we presented a narrative summary of the included studies.

We recorded and synthesised the following to characterise educational interventions.

- Educational content (primary material, learning outcomes, theoretical underpinning).
- Teaching attributes of training programmes used (staff and resource requirements, length of course, methods including whether e‐learning, asynchronous or synchronous, any follow‐up service or session).
- Any knowledge assessment, including method used and reported pre‐ and post‐test scores.

#### Subgroup analysis and investigation of heterogeneity

In case of heterogeneity, we planned to investigate possible causes and address them using methods described in the *Cochrane Handbook for Systematic Reviews of Interventions* (Higgins 2020). We planned to undertake subgroup analyses of potential effect modifiers if there were 10 studies or more. If enough data were available, we planned to perform subgroup analyses by age, gender and disease type for all primary outcomes, as these are the most likely to impact the pedagogical methods (Gordon 2011) and content of education (Hoffman 2014).

There were not sufficient studies included and so these analyses did not take place.

#### Sensitivity analysis

Where enough data were available, we planned to undertake sensitivity analyses on the primary outcomes, to assess whether the findings of the review were robust to the decisions made during the review process. In particular, we excluded studies at high or unclear risk of bias in any field except for performance bias from analyses that had a mix of studies with different risk of bias judgements. Where data analyses included studies with reported and estimated standard deviations, we planned to exclude those with estimated standard deviations to assess whether this affected the findings of the review. We investigated whether the choice of model (fixed‐effect versus random‐effects) impacted the results to explore heterogeneity. For quality of life, when a mixture of validated and unvalidated measures were used, we performed a sensitivity analysis with only validated measures (e.g. Inflammatory Bowel Disease Questionnaire (IBDQ).

#### Summary of findings and assessment of the certainty of the evidence

We presented the main results in a summary of findings table. Each comparison and primary outcome was exported to GRADEprofiler software (developed by the GRADE Working Group) for quality assessment (GRADE 2015). We included all primary outcomes. Based on risk of bias, inconsistency, imprecision, indirectness and publication bias, we rated the certainty of the evidence for each outcome as high, moderate, low or very low. These ratings have been defined as follows.

- **High certainty:** we are very confident that the true effect lies close to that of the estimate of the effect.
- **Moderate certainty:** we are moderately confident in the effect estimate; the true effect is likely to be close to the estimate of the effect, but there is a possibility that it is substantially different.
- **Low certainty:** our confidence in the effect estimate is limited; the true effect may be substantially different from the estimate of the effect.
- **Very low certainty:** we have very little confidence in the effect estimate; the true effect is likely to be substantially different from the estimate of effect.

We justified all decisions to downgrade the quality of studies using footnotes and we made comments to aid reader's understanding of the review where necessary.

## Results

### Description of studies

Information on the results of the search, included and excluded studies, and risk of bias assessment is provided below.

#### Results of the search

We completed our literature search on 27 November 2022, identifying a total of 4046 records through database searching. After removal of duplicates, 3334 unique records remained. Title and abstract screening revealed 112 records for full‐text review. After assessing all 112 records, we identified 34 records of 14 studies that met the inclusion criteria and were included in the review. We also identified seven records of six ongoing studies, and 27 records of 20 studies awaiting classification (five of the studies awaiting classification were identified during the update search for this review and will be included in the analysis when this review is updated). We excluded 44 records of 37 studies for various reasons (see Characteristics of excluded studies). The results of the search are presented in a PRISMA flow diagram (Figure 1).

**1:**
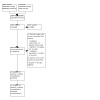


#### Included studies

Additional details on the studies, participants, and interventions can be found in Table 4, Table 5, and Table 6.

**1:** Study and participant details

**Study ID**	**Trial registration**	**Disease type (IG/CG)**	**Disease state (relapse/remission) (IG/CG)**	**Disease duration**	**Numbers randomised (IG/CG)**	**Concurrent therapies (number of participants in IG/CG)**
Berding 2017	NR	IBD (UC and CD) for study completers IG UC/CD (n = 86): 57%/43% = 49/37 CG UC/CD (n = 95): 52.6%/47.4% = 50/45	All in remission	Mean (SD) years IG: 10.9 (10.8), CG: 9.6 (8.9)	IG: 105 CG: 102	5‐aminosalicylic acid: IG 57.8%; CG 64.9% Steroids: IG 28.9%; CG 53.2% Immunosuppressants: IG 45.3%; CG 34.7% Biologicals: IG 10.5%; CG 12.6%
Borgaonkar 2002	NR	IG: CD/UC 18/16 CG: CD/UC: 18/7	IG active/inactive disease: 40% of 34 = 13.6 probably rounded to 14/20 CG active/inactive disease: 48% of 25 = 12/13	Mean (SD) months IG: 96.4 (85.21), CG: 43 (124.2)	IG: 34 CG: 25	Steroids IG: 11 (32%); CG: 5 (20%) Immunosuppressives IG: 3 (9%); CG: 5 (20%) 5‐aminosalicylates IG: 15 (44%); CG: 15 (60%) None IG: 6 (18%); CG: 4 (16%)
Cross 2019	NR	CD participants (n = 236) IG1 (TELE‐IBD EOW): 79 IG2 (TELE‐IBD W): 78 CG: 79 CD participants (n = 112) IG1 (TELE‐IBD EOW): 36 IG2 (TELE‐IBD W): 38 CG: 38	Number of participants with active disease: IG1 (TELE‐IBD EOW): 31 (41%) IG2 (TELE‐IBD W): 25 (36%) CG: 40 (54%) Number of participants in remission: IG1 (TELE‐IBD EOW): 44 (59%) IG2 (TELE‐IBD W): 45 (64%) CG: 34 (46%)	Mean (SD) years IG: 12.4 (9.7), CG: 11.7 (10.0)	IG1 (TELE‐IBD EOW): 115 IG2 (TELE‐IBD W): 116 CG: 117	NR
De Jong 2017	ClinicalTrials.gov (NCT02173002)	IG: 282 (61%) CD patients and 183 (39%) UC patients CG: 262 (59%) CD patients and 182 (41%) UC patients	IG: remission 394 (85%) and active disease 71 (15%) CG: remission 380 (86%) and active disease 64 (14%)	Mean (SD) years IG: 12.8 (10.4), CG: 13.1 (10.8)	IG: 465 CG: 444	No medication or mesalazine: IG: 173 (37%); CG: 147 (33%) Immunosuppressive drugs: IG: 122 (26%); CG: 131 (30%) Biological therapy: IG: 170 (37%); CG: 166 (37%)
Jaghult 2007	NR	CD IG/CG: 26/16 UC IG/CG: 26/16	All participants were in remission	Mean (range) years IG: 1.60 (1‐2), CG: 1.59 (1‐2)	IG: 55 CG: 44	NR
Kennedy 2002	NR	IBD (Crohn's or UC)	Active disease CG: 85 (23.3%) IG: 69 (29.6%) Relapse in past 18 months CG: 196 (53.7%) IG: 137 (50.7%) In remission—no flare‐ups in past 18 months CG: 58 (15.9%) IG: 47 (17.4%)	Diagnosed in the past year: IG: 15/119 CG: 21/121 Diagnosed over 20 years ago: IG: 14/119 CG: 12/121	IG: 119 (9 clusters) CG: 121 (10 clusters)	NR
Moreau 2021	NCT02550158	Number(%) IG: CD 95 (71.4%); UC 38 (28.6%) CG: CD 97 (75.2%); UC: 32 (24.8%)	NR	Median (IQR) months IG: 49.5 (6.4‐111.9), CG: 40.6 (7.3‐ 122.8)	IG: 133 CG: 130	Steroids: IG: 39 (92.5%); CG: 107 (83.0%) Thiopurines or methotrexate: IG: 94 (70.7%); CG: 83 (64.3%) Anti‐TNF‐α (infliximab or adalimumab): IG: 80 (60.2%); CG 77 (59.7%)
Nikolaus 2017	DRKS00008905	All participants had UC.	Clinical activity index used to measure disease state (CAI) CAI 0–4 (remission): IG: 82 (65.1%), CG: 89 (73%) CAI > 4–9 (mild to moderate activity): IG 25 (19.9%), CG 14 (11.5%) CAI > 9 (severe activity/relapse): IG 3 (2.4%),CG 5 (4%) Missing: IG: 16 (12.7%), CG: 14 (11.5%)	Median (range) years IG: 5.34 (0.35–40.36), CG: 5.71 (0.27–26.64)	IG: 126 CG: 122	Steroids: IG: 84 (67.7%); CG: 93 (76.2%) Mesalamine: IG: 124 (98.4%); CG: 122 (100%) Sulphasalazine: IG: 6 (5%); CG: 9 (7.6%) Azathioprine: IG: 54 (43.6%); CG: 56 (46.3%) Methotrexate: IG: 9 (7.3%); CG: 7 (5.8%) Cyclosporine: IG: 2 (1.6%); CG: 3 (2.5%) Tacrolimus: IG: 2 (1.6%); CG: 2 (1.7%) Anti‐TNF: IG: 31 (25%); CG: 13 (10.7%)
Oxelmark 2007	NR	Both UC and CD. UC: IG: 11; CG: 6 CD: IG: 13; CG: 14	All patients were in remission or had low disease activity at inclusion, but numbers were not specified.	Mean (range) years IG: 4.6 (1‐11), CG: 5.2 (1‐10)	IG: 24; CG: 22	Prednisolone (< 10 mg): IG: 10; CG: 3 Budesonide: IG: 1; CG: 0 5‐aminosalicylic acid/sulfasalazine: IG: 8; CG: 5 Immunomodulator: IG: 9; CG: 5 Antibiotics: IG: 4; CG: 4 None: IG: 5; CG: 7
Uran 2019	NR	IG (web‐based education): 16 UC and 14 CD CG (standard education): 16 UC and 14 CD	IG: Disease activity of UC: Remission: 5 Mild disease: 5 Severe disease:6 Disease activity of CD: Remission: 5 Mild disease: 8 Severe disease: 1 CG: Disease activity of UC: Remission: 4 Mild disease: 8 Severe disease: 4 Disease activity of CD: Remission: 9 Mild disease: 4 Severe disease: 1	Mean (SD) months IG: 82.23 (54.52), CG: 81.93 (56.71)	IG (web‐based education): 30 CG (standard education): 30	NR
Uran 2019	NR	Both UC and CD. 67% of participants had CD and 33% had UC. Numbers not specified for IG and CG	All randomised participants had inactive disease at baseline.	NR	IG: 7; CG: 6	All participants were prescribed at least 1 daily oral medication for the control of IBD (i.e. steroid, thiopurine, or aminosalicylate) but specific figures not given for IG and CG.
Walkiewicz 2011	NR	IBD (UC and CD). Specific numbers for IG and CG NR	NR	NR	Total randomised 36 Specific numbers for IG and CG NR	NR
Waters 2005	NR	Both UC and CD UC/CD: IG: 14/31, CG: 18/26	Mean (SD) Measured using the Crohn's Disease Activity Index (CDAI) and Activity Index (mean score): IG: 126.8 (93.3); CG: 188.3 (117.1) Activity index (mean score): IG: 111.8 (25.8); CG: 114.1 (37.8)	Mean (SD) years IG: 10.5 (9.0), CG: 13.4 (9.84)	IG: 45 CG: 44	Steroids: IG:3 (7); CG: 9 (20) Azathioprine/6‐mercaptopurine: IG: 9 (20); CG: 9 (20) Methotrexate: IG: 1 (2); CG: 1 (2) 5‐aminosalicylate: IG: 12 (27); CG: 22 (50) Antibiotics (chronic therapy): IG: 3 (7); CG: 3 (7) Monoclonal antibody: IG: 4 (9); CG: 3 (7) Osteoporosis therapy: IG: 9 (20); CG: 13 (29) Alternative therapy: IG: 3 (7); CG: 6 (14)
Weizman 2021	NCT02569333	91 patients with UC	All participants with active disease flare up	NR	IG: 46; CG: 45	5‐aminosalicylate: IG: 16 (36%); CG:18 (43%) Steroids: IG:18 (40%); CG:21 (50%) Thiopurine: IG:3 (7%); CG:7 (17%) Anti‐TNF: IG:12 (27%); CG:16 (38%)

**CAI:** Colitis Activity Index; **CD:** Crohn's disease; **CG:** control group**;HRQoL:** health‐related quality of life; **IBD:** inflammatory bowel disease; **IG:** Intervention group; **IQR:** interquartile range; **SD:** standard deviation; **TNF‐α:** tumour necrosis factor alpha; **UC:** ulcerative colitis

**2:** Intervention details

**Study ID**	**Intervention description**	**Educational content (primary material, learning outcomes, theoretical underpinning)**	**Control intervention description**	**Type of control intervention**	**Intervention length**	**Outcome measurement points**	**Follow‐up measurement points**
Berding 2017	A two‐part patient education seminar over 2 days (11.5 hours)	Patient education seminar involving tasks and discussions. First part covered medical information about the anatomy and function of the digestive system, epidemiology, pathogenesis, clinical aspects, diagnosis and therapy (pharmaceutical and surgical), complications, extraintestinal manifestations as well as nutrition and pregnancy. Second part covered coping and self‐management skills. It included moderated exchange of experiences and individuals strategies for coping with pain and negative emotions. Also, use of worksheets to address stress prevention and self‐care. Finally, use of patient vignettes to discuss when and how to discuss confidently about suffering with IBD. **Theoretical underpinning:** NR **Learning outcomes:** The aim of the seminar was to empower the patients to cope with living with IBD	Treatment as usual (no education)	Waitlist control	2 days	At 2 weeks after the end of the seminar	At 3 months after the seminar
Borgaonkar 2002	Information booklets available from the Crohn’s and Colitis Foundation of Canada served as the educational intervention.	Primary material content: the booklets administered to the education group covered the following topics: general information about IBD, such as the symptoms, complications, treatments, prognosis, and possible etiologies; currently available medications, efficacy, side effects, and the rationale for choosing them; the role of surgery in the management of IBD including the available procedures and their indications; issues of sexuality, fertility, and pregnancy, and how these might be affected by IBD and its therapies. **Theoretical underpinning:** NR **Learning outcomes:** NR	Standard therapy	No details	2 weeks	End of study (2 weeks)	None
Cross 2019	Delivering educational messages through a mobile telemedicine system for IBD patients. There were two intervention groups where IG1 (TELE‐IBD EOW) received a message every other week, IG2 (TELE‐IBD W) received the messages weekly, and CG did not receive any messages.	TELE‐IBD was designed using a mobile phone for participants and a decision support server and website for staff and providers. The website provided an interface for staff and participant profiles and collected data from each testing session. The provider could individualise alerts and action plans for each participant. TELE‐IBD participants received educational tips and periodic “pragmatic” educational messages at the discretion of the provider. The content was based materials from the Crohn’s and Colitis Foundation. The messages were a short factual summary about IBD like "What is IBD" or "short summary of immunosuppressants and its side effects" **Theoretical underpinning:** NR **Learning outcomes:** NR	Standard care	Standard of care was based on current evidence‐based professional guidelines including a comprehensive assessment, a guideline‐concordant therapy plan, scheduled and as needed visits, scheduled and as needed calls, and administration of fact sheets about disease‐specific topics. Administration of educational materials for control participants was not standardised and was at the discretion of the treating provider. The treating physician of the participant could provide educational materials as needed throughout the study. For example, if a patient was changing therapy, the provider could give information about the drug to be started (infliximab, adalimumab, certolizumab, etc).	12 months	At baseline, 6 months and 12 months at end of the intervention Author stated "incomplete assessment of disease knowledge at baseline and follow‐up for participants"	At 12 months
De Jong 2017	IG: participants received instructions for accessing the telemedicine system (myIBDcoach) which is a secured webpage with an HTML application for tablet or smartphone. The system includes monthly monitoring modules about disease activity, medication use, etc. The system also includes questions on factors affecting disease like nutritional status, smoking, etc. Participants also had access to e‐learning modules	The main e‐learning components were interactive patient‐tailored information, on topics such as medications, adherence to medication, smoking cessation, (mal)nutrition, methods to prevent or reduce symptoms (self‐management), fatigue, work productivity, anxiety, and depression **Theoretical underpinning:** NR **Learning outcomes:** NR	CG: those participants continued their routine follow‐up visits following the local protocol, with an opportunity to schedule an extra visit if symptoms relapsed	No details	12 months	At baseline, 6 months and 12 months at end of the intervention	NR
Jaghult 2007	Three weekly 2‐hour multi‐professional group‐based education programme sessions held with Crohn's disease participants in separate groups from UC participants.	The topics for the first session included the aetiology and nature of the diseases, examinations, medical treatments, efficacy, side effects and new research. At the second session the participants were informed and educated about the importance of nutrition, economic issues, psychological reactions, coping and behavioural changes. At the third and last session, information was provided concerning the organisational and care of IBD patients at the clinic. At this session a sigmoidoscope and a proctoscope were demonstrated for the patients. The content was based on clinical experience, literature studies and contacts with other gastroenterological clinics with experience of similar education programmes. **Theoretical underpinning:** NR **Learning outcomes:** NR	Regular information	Participants received regular information during visits to the IBD clinic	3 weeks	IG: at 1 month and 6 months CG: at 6 months	NR
Kennedy 2002	Guidebooks for both CD and UC	Guidebook divided into 2 parts. First part contained lay and traditional evidence‐based information about the UC/CD. Second part was a record book for participant and doctor to write details of diagnosis, tests, treatments, symptoms and self‐management plans. Guidebook was developed with patients prior to the study, and was based on experiences of patients living with IBD and their specific information requirement. The aim of the guidebook is to increase patient involvement in the management of their IBD through, self‐management shared care and decision‐making. **Theoretical underpinning:** NR **Learning outcomes:** NR	Participants continued to receive IBD management as deemed by specialist doctor as usual	NR	Package including the guidebook was accessible for 1 year	IBDQ score was measured at the start and end of the trial	NR
Moreau 2021	Education programme (EDU‐MICI) delivered by a dedicated staff (mainly nurses) using an illustrated book, covering the different dimensions of life with IBD.	The sessions were standardised in all the centres and were based on an illustrated book (portfolio) that reviewed different aspects of the disease: aetiology, evolution, treatment, and social and personal problems. The five main topics raised during the sessions were: ‘To organise my daily life and improve my quality of life’ ‘To understand my disease’ 'To talk about my disease and express my needs’ ‘To benefit from my care and treatments’, and ‘To consider preoccupations of a young IBD patient’. **Learning outcomes:** Better patient knowledge of the disease, its management and principles of treatment, could improve disease outcomes and decrease impact on daily life. **Theoretical underpinning:** NR	No education programme during first 6 months. After 6 months, there was a cross‐over procedure and participants from the control group followed the same programme as the educated group.	Waitlist control	4‐6 months	At 6 months	At 12 months
Nikolaus 2017	A standardised education programme delivered using standardised slide set, followed by a group session in which all participants asked questions and a contact for further individual questions (e.g. by telephone or email) was established.	The education programmed consisted of a slide presentation of at least 2 h and consecutive discussion. The presentation comprised modules summarising aetiology of UC, course of disease, complications, therapy regimen (including the necessity and benefits of mesalamine therapy), and strategies to prevent acute relapses. **Theoretical underpinning**: NR **Learning outcomes:** NR	Participants received standard care and were offered participation in the education programme also after termination of the study. No further description given of "standard care".	Waitlist control.	Education was administered during a dedicated study visit between day 0 and Week 4.	At week 8	At month 5, month 8, month 11 and month 14 after the intervention
Oxelmark 2007	Nine different sessions comprising lectures alternating with group therapy.	**Lectures:** Covered aetiology of IBD and the different stages. Medical treatment, efficacy, side effects and new research results were outlined. The anatomy and physiology of the gut were explained. A video‐endoscope and a rigid sigmoidoscope were demonstrated. Surgical interventions were explained and information given on diet. Information about the Swedish Association of People with Stomach and Bowel Diseases was provided. **Group therapy:** Psychological education covering psychological reactions, receiving information of the diagnosis, coping, stress management, positive and negative stress, and self‐image. **Theoretical underpinning:** NR **Learning outcomes:** Educational programmes could enhance the sense of control and skills in coping with the relapses of the diseases and its complications and the long‐term effects of having a chronic disease.	Participants in the control groups received conventional “on demand” medical and psychosocial/psychological treatment during the study period.	NR	Approximately 3 months	At 6 months and 12 months after study start	NR
Uran 2019	**IG:** (web‐based education): which presented information via online website, participants had access to this website via using a username and password which were created for each participant, and they were informed about how to use the website by means of a slide show.	The content and scope for both IG (web‐based) and CG (standard education) were exactly the same. The content of the education material was about the definitions of IBD, UC, CD, anatomy, and physiology of IBD. It also contained information about indications, diagnostic tests, treatment principles, the importance of drug use, nutritional principles, and specific descriptions for special cases such as pregnancy, sexuality and puberty. The researcher relied on literature (referenced in the paper) to build up the educational materials. **Theoretical underpinning:** NR **Learning outcomes:** NR	**CG:** (standard education): which presented information via easy‐to‐read, illustrated, colour‐printed books	NR	8 weeks	At 2 weeks, 4 weeks and 8 weeks	NR
Vaz 2019	An educational session using the IBD Pocket Guide.	Participants randomised to the IG met individually with the educator for a 30‐minute educational intervention session. Educational content was delivered using the IBD Pocket Guide. The IBD Pocket Guide provides an overview of gastrointestinal function and anatomy, information about gastro‐intestinal procedures, and information on common medications as well as importance of medication adherence. The guide provides tips for adherence promotion, transition readiness, and information on where to obtain additional resources about IBD and self‐management. The guide can be personalised for each patient. **Theoretical underpinning**: NR **Learning outcomes:** NR	Participants received usual care. No details explaining "usual care"	Waitlist control. The CG was offered the educational intervention after the final assessment.	30 minutes	At 4 weeks after the intervention.	NR
Walkiewicz 2011	IG1: "Internet blog access" IG2: "the receipt of text messaging" IG3: "combination of Internet blog access and text messaging."	NR	Standard care	NR	3 months	NR	NR
Waters 2005	In addition to standard of care, patients in the IG attended a structured education programme.	The education programme included general information about basic gut and immune system anatomy and physiology, explored the pathophysiology of IBD, and reviewed current and future therapy. Group discussion about disease management was tailored to address the identified worries and concerns of the subjects derived from baseline data. Participants received copies of each presentation, a booklet on IBD medication and management, and an overview of the group discussion information. **Theoretical underpinning:** NR **Learning outcomes:** NR	Received standard care consisting of physician visits, at the discretion of the physicians and patients, with physician‐directed ad hoc teaching during visits and the presentation of printed educational literature. Printed educational literature included that provided by the Crohn’s and Colitis Foundation of Canada and local gastroenterologists.	Waitlist control. The control group was offered the full education programme after the study data collection was completed.	4 weeks	Immediately post‐education (4 weeks from study start) and 8 weeks post‐education.	NR
Weizman 2021	IG: participants were provided with an iPad containing patient‐directed educational material which focused on the optimal in‐hospital management of acute severe UC.	The educational intervention was an original, interactive video that provided a summary of the 2012 Canadian consensus statements on the treatment of hospitalised adult patients with severe UC, and it used a patient‐friendly languages and images. **Theoretical underpinning:** NR **Learning outcomes:** Education and awareness of IBD guideline‐based management strategy could lead to “a greater sense of control in management, engagement in the care process and understanding of the overall management plan which translated to the observed improvements in trust in physician and satisfaction”	Standard care	NR	NR (Participants could access the educational material on demand throughout the hospital admission)	At discharge and after 6 months	NR

**CG:** control group**;IBD:** inflammatory bowel disease; **IG:** Intervention group; **NR:** not reported; **TELE‐IBD W:** group that received a telemedicine message every week; **TELE‐IBD EOW:** group that received a telemedicine message every other week

**3:** Education details

**Study ID**	**Teaching attributes of training programmes used (staff and resource requirements, length of course, methods including whether e‐learning, asynchronous, synchronous or self‐directed, any follow‐up service or session).**	**Any knowledge assessment, including method used** **(Formative or summative)**	**Is the intervention part of a package of measures (e.g. diagnostic tools etc)?**	**Who or what is delivering the intervention**	**Resources required for the intervention to happen and who provides them**	**Access issues as reported on studies (disabilities, financial issues etc)**
Berding 2017	A one‐off face‐to‐face seminar lasting for 2 days (day 1 lasted 8 hours and day 2 lasted 3.5 hours.). **Synchronous** It was provided to batches of about 15 participants with about16 sessions held. The intervention followed a manual written by gastroenterologists and a psychologist. It considered the aims and principles of self‐management patient education, the expertise of the project’s advisory board (gastroenterologists, a nutritionist, a surgeon, and representatives of medical societies), recommendations of a centre for patient education, and the results of a formative evaluation. A focus group of IBD patients also provided input about needs and expectations concerning patient education.	NR	No	Conducted by IBD physician specialists experienced in performing patient education. The second part on coping and self‐management skills was held by a psychologist.	A manual (protocol) written by gastroenterologists and a psychologist.	Patients with insufficient language skills, severe vision or hearing impairment, serious physical or psychological comorbidity were excluded.
Borgaonkar 2002	Asynchronous: to be read within 2 weeks 4 booklets	NR	No	Booklets	Booklets provided by the research team and developed by Crohn's and Colitis Canada	"These pamphlets are freely available to most IBD patients, irrespective of socioeconomic status and learning ability"
Cross 2019	**Asynchronous** as the IG received educational text messages which were based on materials from the Crohn’s and Colitis Foundation were delivered every other week for IG1 (TELE‐IBD EOW) and once weekly for IG2 (TELE‐IBD W)	**Summative assessment** (There was no continuous assessment or feedback during the intervention) Participant knowledge was assessed with the Crohn’s and Colitis Knowledge (CCKNOW) survey the CCKNOW is a 30‐item questionnaire, with 1 point given for each correct answer.	No	Educational text messages which were sent to IGs mobiles. There was no mention of who was sending these messages.	Educational curriculum was developed based on materials from the Crohn’s and Colitis Foundation which was sent over phones.	NR
De Jong 2017	**Asynchronous** Educational component was in form of an interactive e‐learning module on various subjects, allowing participants to review modules when they or their health‐care providers considered it desirable.	NR	Yes, monitoring modules, which contained questions regarding disease activity, medication use etc. The system also included questions on biopsychosocial aspect of the disease like nutritional status, anxiety and social support. The system included intensified monitoring modules, outpatient visit modules, e‐learning modules, a personal care plan, and an administrator page used by the health‐care provider.	E‐learning modules	Access to computer, tablet, or smartphone	People without access to computer, tablet, or smartphone were excluded.
Jaghult 2007	The educational programme took place over 3 weeks (1 session per week for 2 hours). The session was delivered to groups of 8 to 10 participants, and each participant was invited to bring a significant other of his/her own choice. Participants with CD and those with UC were divided into separate groups **Synchronous** The sessions were face‐to‐face. In every session there was time to ask questions and to discuss personal experiences. At the last session, the participants received a written summary of the contents of the education programme.	NR	No	A specialist nurse, gastroenterologist, dietician and medical social worker gave the lectures. The specialist nurse worked as a co‐ordinator and attended every meeting.	The specialist nurse worked as a co‐ordinator for the project and attended every meeting.	Participants that did not have a good understanding of the Swedish language and those that could not complete a questionnaire.
Kennedy 2002	Patient‐centered consultations conducted by a clinician during which self‐management plans were negotiated and written in a guidebook. It was a mixture of **synchronous and asynchronous** and participants were asked to telephone a specific number if they require an unscheduled appointment according to the circumstances listed in the guidebook.	NR	Yes, other components of the package included guided self‐management, direct access to services and patient‐centred approach to care.	Participants went through the guidebooks themselves and the clinicians wrote the self‐management plan in the guidebook during the consultation.	Clinicians were given a two‐hour training to empower them with the skills to deliver the intervention.	Inability to write in English
Moreau 2021	At least two health professionals per centre were trained to become ‘educators’, following 50 h (8 days) of training. All the educators performed at least 10 education sessions. **Synchronous** It was a face‐to‐face session. The education programme lasted for 6 months.	**Summative assessment** Knowledge assessed using Étude randomisée et contrôlée évaluant l'impact du programme d'éducation (ECIPE) sub‐score pre‐ and post‐intervention. Raw scores were given for the pre‐test but not for the post test.	No	Education was performed by a dedicated staff (mainly nurses) who received 50 hours of training.	A scientific committee, including professionals from GETAID and a patients’ association, ‘Association François Aupetit (AFA)’, designed the specific education programme 'EDU‐MICI'.	Patients unable to communicate, understand, or participate in the educational programme, mainly for linguistic reasons were excluded.
Nikolaus 2017	The education programme was delivered through a standardised slide presentation. The slide presentation lasted for at least 2 hours. It was a mixture of **synchronous and asynchronous** methods. The education programme included a group session in which all participants asked questions and a contact for further individual questions (e.g. by telephone or email) was established.	NR	No	The education programme was delivered by either a certified nurse or the trial physician, who underwent a mandatory training programme beforehand to ensure standardised delivery of the programme training.	The interventions took place at the participating centres of the German National IBD Study Group (GISG).	NR
Oxelmark 2007	Nine different sessions (once a week, each session lasted for 1.5 hours) for about 3 months. **Synchronous:** lecture sessions included time for questions and discussions. At the final session all participants were given the opportunity to ask additional questions or to discuss issues that had emerged during the lectures and the group therapy sessions.	NR	Yes. The other part was the group therapy session which has been described.	The lectures were presented by a gastroenterologist and specialist nurse. The group therapy sessions in the present study were led by a medical social worker/psychotherapist. The gastroenterologist, specialist nurse, and medical social worker/psychotherapist all participated in the final session.	NR	NR
Uran 2019	**Asynchronous** as the IG were able to access the educational material using an online website or for CG read colour‐printed books.	NR	No	Self‐study where patients had to read the material themselves via book or website.	NR	Those that were unable to use computer, internet and mobile phone.
Vaz 2019	IBD Pocket Guide was used in delivering the session. The session lasted for 30 minutes. **Synchronous** The participants met individually with the educator for the educational intervention session.	**Summative assessment** IBD knowledge was assessed using the IBD Knowledge Inventory Device (The IBD‐KID) It was used to evaluate pre‐post changes in overall knowledge and in 4 domains: gastro‐intestinal anatomy, general IBD knowledge, medications, and nutrition.	No	The session was delivered by an educator. No further information was given about the educator.	The IBD Pocket Guide (digital content)was developed specifically for this study and is inexpensive. It was created in collaboration with paediatric IBD specialists, psychologists, social workers, pharmacists, and parents of patients with IBD.	NR
Walkiewicz 2011	Blogs were posted twice weekly. Text messages were also sent out twice weekly. **Asynchronous**	Disease‐related knowledge was assessed using a modified version of the Crohn's & Colitis Foundation of America (CCFA) Knowledge Score (I‐M‐AWARE) Not enough information provided to determine whether it was summative or formative.	No	Content for the blogs and text messages were determined by paediatric and adult gastroenterologists specialising in IBD.	NR	NR
Waters 2005	The overall duration of the education programme was 12 h, provided in 3 h blocks over four consecutive weeks. **Synchronous:** The principles of adult teaching and learning were applied, and a variety of teaching strategies were used to enhance learning and improve critical thinking skills.	**Summative assessment** The KQ and CCKNOW were used to assess knowledge levels in five topic categories: diet gut anatomy and physiology general IBD knowledge complications, and medications. This was measured at baseline, immediately after the intervention and at 8 weeks after the intervention.	No	The education programme was designed and provided by a Nurse Practitioner. A dietitian provided nutrition management education tailored to the diseases and their common complications. A surgeon presented information about surgical interventions, focusing on how surgical options are determined and the benefits of surgery.	NR	Participants unable to attend the education programme (e.g. due to lack of transportation) and those not fluent in written and spoken English were excluded.
Weizman 2021	The education programme lasted for 6 months **Asynchronous:** participants had to do self‐directed learning	NR	No	The educational material was based on an original, interactive video that provided a summary of managing UC using patient‐friendly languages and images. Who made and appeared in the video was not reported.	Video of the 2012 Canadian consensus statements on the treatment of hospitalised adult patients with severe UC.	NR

**CCKNOW:** Crohn's and Colitis Knowledge questionnaire; **IBD:** inflammatory bowel disease; **KQ:** Knowledge Questionnaire; **NR:** not reported; **TELE‐IBD W:** group that received a telemedicine message every week; **TELE‐IBD EOW:** group that received a telemedicine message every other week

##### Setting

Fourteen RCTs involving a total of 2708 participants met our inclusion criteria. Three studies were conducted in the USA (Cross 2019; Vaz 2019; Walkiewicz 2011), three in Canada (Borgaonkar 2002; Waters 2005; Weizman 2021*),* two in Germany (Berding 2017; Nikolaus 2017), two in Sweden (Jaghult 2007; Oxelmark 2007), one in the UK (Kennedy 2002), one in France (Moreau 2021), one in the Netherlands (De Jong 2017), and one in Turkey (Uran 2019*).* All the included studies were conducted in hospitals and tertiary centres*.* Seven studies were single‐centre (Borgaonkar 2002; Jaghult 2007; Oxelmark 2007; Uran 2019; Vaz 2019; Walkiewicz 2011; Waters 2005), and seven were multi‐centre (Berding 2017; Cross 2019; De Jong 2017; Kennedy 2002; Moreau 2021; Nikolaus 2017; Weizman 2021). Two studies were cluster‐RCTs (Kennedy 2002; Weizman 2021).

##### Participants

Age ranged from 11 years in Walkiewicz 2011 to 75 years in De Jong 2017. There were two studies in paediatric populations (Vaz 2019; Walkiewicz 2011). Vaz 2019 included adolescents between 11 and 18 years of age, and Walkiewicz 2011 participants between 11 and 21 years of age. Both interventions were targeted towards the participating adolescents and not towards their caregivers.

Two studies examined exclusively ulcerative colitis (UC) populations (Nikolaus 2017; Weizman 2021), whilst the remaining studies examined a mix of IBD patients (Berding 2017; Borgaonkar 2002; Cross 2019; De Jong 2017; Jaghult 2007; Kennedy 2002; Moreau 2021; Oxelmark 2007; Uran 2019; Vaz 2019; Walkiewicz 2011; Waters 2005).

Six studies examined participants in both active and inactive states of the disease (Borgaonkar 2002; Cross 2019; De Jong 2017; Kennedy 2002; Nikolaus 2017; Uran 2019); two studies examined participants in an inactive state of the disease (Jaghult 2007; Vaz 2019); one study examined participants in an active state of the disease (Weizman 2021); two studies examined participants in remission or low disease activity (Berding 2017; Oxelmark 2007). One study reported the disease activity of its participants as a mean value using the Crohn's Disease Activity Index (CDAI) and the Activity Index (AI) (Waters 2005). Two studies did not report on activity of the disease (Moreau 2021; Walkiewicz 2011).

Four of the studies had trial registrations *(*De Jong 2017; Moreau 2021; Nikolaus 2017; Weizman 2021).

##### Interventions

The following interventions were assessed in the included trials.

- A 2‐part patient education seminar versus *“*treatment as usual” (Berding 2017).
- Information booklets available from the Crohn’s and Colitis Foundation of Canada versus *“*usual care” (Borgaonkar 2002).
- Weekly educational text messages versus once every other week educational text messages versus routine clinic visits (Cross 2019).
- E‐learning module accessible via telemedicine system (myIBDcoach) versus routine follow‐up visits (De Jong 2017).
- Multi professional group‐based education programme versus regular information during visits to the IBD clinic (Jaghult 2007).
- Guidebooks for Crohn's Disease (CD) and UC versus *“*standard care” (Kennedy 2002).
- Education programme delivered by a dedicated staff using an illustrated book versus no intervention (Moreau 2021).
- A standardised education programme, followed by a group session versus standard care (Nikolaus 2017).
- Nine sessions of lectures alternating with group therapy versus conventional “on demand” medical and psychosocial/psychological treatment (Oxelmark 2007).
- Web‐based education versus education which presented information via easy‐to‐read, illustrated, colour‐printed books (educational content was exactly the same for both groups) (Uran 2019).
- A 30‐minute educational session using the IBD Pocket Guide versus usual care (Vaz 2019).
- Internet blog access versus the receipt of text messaging versus Internet blog access and receipt of text messaging versus standard care (Walkiewicz 2011).
- Structured education programme and standard care versus standard care consisting of physician visits, at the discretion of the physicians and patients, with physician‐directed ad hoc teaching during visits and the presentation of printed educational literature (Waters 2005).
- Original, interactive video that provided a summary of the 2012 Canadian consensus statements on the treatment of hospitalised adult patients with severe UC versus standard care (Weizman 2021).

##### Outcomes

The length of the interventions ranged from 30 minutes, in Vaz 2019, to 12 months in De Jong 2017.

###### Primary outcome: Disease activity

Only four studies mentioned disease activity as an outcome. Berding 2017 measured IBD disease activity as a continuous outcome using the Bowel Disease Activity Index (GIBDI), and Cross 2019 used the Crohn's Disease Harvey‐Bradshaw Index (HBI) for CD participants and the Simple Clinical Colitis Activity Index (SCCAI) for patients with UC/indeterminate colitis. In Nikolaus 2017 the authors stated disease activity as an outcome, and that they measured it using the Colitis Activity Index (CAI), however the data were not presented. Uran 2019 reported the numbers of participants with mild and severe disease at each stage of the study.

###### Primary outcome: Flare‐ups or relapse

Five studies measured flare‐ups or relapse. De Jong 2017 and Kennedy 2002 evaluated mean number of flare‐ups (SD) during the study as continuous data. Nikolaus 2017 reported numbers with acute relapse per group with relapse defined as clinical activity index ≥ 9. Oxelmark 2007 and Vaz 2019 also reported numbers of patients with relapse during the study.

###### Primary outcome: Quality of life

Ten studies reported quality of life (Berding 2017; Borgaonkar 2002; Cross 2019; De Jong 2017; Jaghult 2007; Kennedy 2002; Moreau 2021; Oxelmark 2007; Uran 2019; Waters 2005). The Inflammatory Bowel Disease Questionnaire (IBDQ) was used in seven studies, (Borgaonkar 2002; Cross 2019; Jaghult 2007; Kennedy 2002; Oxelmark 2007; Uran 2019; Waters 2005). The short Inflammatory Bowel Disease Questionnaire (SIBDQ) was used by De Jong 2017* *and Moreau 2021*.* The SF‐12 short form health survey was used by Berding 2017. Borgaonkar 2002 also used the Quality Index in Crohn’s and Colitis (QuICC) questionnaire, and Jaghult 2007 the Rating Form of IBD Patient Concerns (RFIPC).

###### Secondary outcome: Number of episodes accessing health care

Four studies stated the number of episodes of accessing health care (Cross 2019; De Jong 2017; Kennedy 2002; Waters 2005). Cross 2019 reported total encounters, IBD‐related hospitalisations, non‐IBD‐related hospitalisations, non‐invasive diagnostic tests, electronic encounters and telephone encounters, all as rates, adjusted for 100 participants per year. De Jong 2017 reported hospital admissions and emergency visits, Kennedy 2002 reported kept hospital appointments and numbers of patients who did not attend. Moreau 2021 measured hospitalisations, and Waters 2005 rate of healthcare use.

###### Secondary outcome: Change in disease activity

No studies reported this outcome.

###### Secondary outcome: Change in quality of life

Only one study reported the change in quality of life in its participants (Borgaonkar 2002). The study used the IBDQ (the questionniare has 32 questions and the score ranges from a minimum of 32 to a maximum of 224, but the authors presented results as mean scores for each question with a range; high score = better result) and the QuICC (range 1 = excellent to 5 = poor) at the start and after two weeks of the intervention to report the mean values (SD) on its sample.

###### Secondary outcome: Medication adherence

Five studies measured medication adherence (De Jong 2017; Moreau 2021; Nikolaus 2017; Vaz 2019; Waters 2005). De Jong 2017, Moreau 2021, and Nikolaus 2017 used the Morisky Medication Adherence Scale. Vaz 2019 reported adherence rates based on recordings with the MedMinder system. Waters 2005 reported incidents and rates of missed medications, and rate of non‐adherence as measured by the Patient Satisfaction Questionnaire and participant self‐report.

###### Secondary outcome: Patient knowledge and/or skill

Patient knowledge/skills was reported in seven studies (Berding 2017; Cross 2019; De Jong 2017; Moreau 2021; Vaz 2019; Walkiewicz 2011; Waters 2005).

Cross 2019 measured knowledge using the Crohn’s and Colitis Knowledge questionnaire, while Vaz 2019 used the IBD knowledge Inventory Device (IBD‐KID) and Walkiewicz 2011 a modified version of the Crohn's & Colitis Foundation of America (CCFA) Knowledge Score (I‐M‐AWARE).

Waters 2005 used both the Chron's and Colitis Knowledge (CCKNOW) questionnaire and the Knowledge questionnaire (KQ), while it also assessed self‐perceived knowledge on a visual analogue scale (VAS).

Moreau 2021 used the ECIPE (Étude randomisée et contrôlée évaluant l'impact du programme d'éducation (Controlled multicentre study of the impact of a programme of therapeutic Education in IBD)) score they developed for their education programme and defined success as a dichotomous outcome of improvement in patients' skills by an increase of the ECIPE score of more than 20%, from baseline to six months.

In Berding 2017 medical and psychological knowledge was self‐reported by the participants on a Likert scale, while in De Jong 2017 IBD knowledge and medication knowledge were self‐reported on a VAS.

###### Secondary outcome: Total adverse events (serious and minor)

Only two studies reported total adverse events (De Jong 2017; Vaz 2019).

###### Secondary outcome: Withdrawals due to adverse events

Only three studies reported this outcome (Cross 2019; De Jong 2017; Vaz 2019). There were no withdrawals due to adverse events in these studies as no participant reported any adverse events related to use of the telemedicine intervention.

###### Qualitative synthesis: Educational content

The details on the contents of each intervention can be found in Table 5.

Five studies relied on face‐to‐face workshops, seminars or teaching session for delivering their educational content (Berding 2017; Jaghult 2007; Nikolaus 2017; Vaz 2019; Waters 2005). Five used e‐learning or distance learning via mobile phones (Cross 2019; De Jong 2017; Uran 2019; Walkiewicz 2011; Weizman 2021). Three studies used written material as their primary material (Borgaonkar 2002; Kennedy 2002; Moreau 2021). One study used mixed methods of lectures and group therapy for delivering information on IBD and psychological coping methods for IBD, respectively (Oxelmark 2007).

The educational learning outcomes were not clearly stated in any of the studies. Some studies mentioned generic aims such as empowering patients (Berding 2017), enhancing the sense of control and skills in coping with relapses (Oxelmark 2007), and a greater sense of control in management, engagement in the care process and understanding of the overall management plan (Weizman 2021).

None of the studies described the educational theoretical underpinning of their interventions.

###### Qualitative synthesis: Teaching attributes of training programmes used (staff and resource requirements, length of course, any follow‐up service or session)

Six studies employed synchronous interventions (Berding 2017; Jaghult 2007; Moreau 2021; Oxelmark 2007; Vaz 2019; Waters 2005), and six asynchronous interventions (Borgaonkar 2002; Cross 2019; De Jong 2017; Uran 2019; Walkiewicz 2011; Weizman 2021). Two studies were a mix of synchronous and asynchronous (Kennedy 2002; Nikolaus 2017).

Three interventions were part of a package of measures that contained other elements as well (De Jong 2017; Kennedy 2002; Oxelmark 2007).

Staff delivering the interventions included nurses, gastroenterologists and other physicians, psychologists, dietitians, medical social workers and educators. Resources included computers, tablets, smartphones, booklets and other written materials, as well as physical space and equipment for delivering workshops or lectures. Access issues included participants with insufficient language skills, severe vision or hearing impairments, serious physical or psychological comorbidities, people without access to computers, tablets, or smartphones and non‐access to transport (Table 6).

###### Qualitative synthesis: Knowledge assessments (formative or summative assessment)

Four of the five studies that assessed patient knowledge used summative assessment (Cross 2019; Moreau 2021; Vaz 2019; Waters 2005); we did not have enough information to judge the type of assessment in Walkiewicz 2011.

The pre‐ and post‐knowledge scores, or changes in knowledge scores from baseline, are presented in Table 6.

##### Funding sources and conflicts of interest

Nine studies reported their sources of funding (Cross 2019; Berding 2017; De Jong 2017; Kennedy 2002; Moreau 2021Nikolaus 2017; Uran 2019; Vaz 2019; Weizman 2021). Four studies were funded via government grants (Berding 2017; Cross 2019; Kennedy 2002; Vaz 2019), three studies by private sources (De Jong 2017; Nikolaus 2017; Weizman 2021), one study by a non‐profit research association (Moreau 2021), and one study declared that it received no financial support (Uran 2019).

Five studies did not report anything about their source of funding (Borgaonkar 2002; Jaghult 2007; Oxelmark 2007; Walkiewicz 2011; Waters 2005).

Eight studies made declarations about conflicts of interest (Berding 2017; Cross 2019; De Jong 2017; Moreau 2021; Nikolaus 2017; Uran 2019; Vaz 2019; Weizman 2021), and five of these declared no conflicts of interest (Berding 2017; Cross 2019; Nikolaus 2017; Uran 2019; Vaz 2019). One study declared that one of the authors was an employee of the industrial partner that provided funding (Weizman 2021), one study declared that several authors received honoraria from private industrial partners (Moreau 2021), and one study declared that several authors had connections to healthcare companies unrelated to the study (De Jong 2017).

Six studies did not make any declarations about conflicts of interest (Borgaonkar 2002; Jaghult 2007; Kennedy 2002; Oxelmark 2007; Walkiewicz 2011; Waters 2005).

#### Excluded studies

We excluded 37 full‐text studies (44 records) for various reasons. The reasons for exclusion of each study are presented in the Characteristics of excluded studies table and are summarised below.

- Wrong intervention (23 studies) (Cross 2012; Dewulf 2011; Eaden 2002; Elkjaer 2010a; Greenley 2015; Hueppe 2020; IRCT2015041921850N1; Karia 2012; Kyaw 2014; Ley 2020; Long 2020; Maya 2012; Meng 2018; NCT03059186; NCT03186872; NCT04207008; NCT00248742 Reusch 2016; Robinson 2001; Siegel 2018; Sutton 2019; Tsavdaroglou 2019; Wierstra 2018).
- Not RCTs (12 studies) (Bregenzer 2005; Chapman 2020; Cheema 2020; Elkjaer 2010b;Gerbarg 2015;Kamat 2018; Korzenik 2016; Lange 1996; Lim 2020; Schimdt 2018; Tung 2015; Wang 2020).
- Wrong population: (2 studies) (Norton 2015; Zhang 2020).

There are 20 studies awaiting classification (Almario 2022; Atreja 2015; De Dycker 2022; DRKS00022935; Haslbeck 1996; Homel 2015; IRCT20180520039736N1; IRCT20191026045251N1; ISRCTN67674151; Lorenzon 2016; Magharei 2019; Martinato 2022; Menze 2022; Moshkovska 2010; NCT03695783; NCT04183608; Otilia 2019; Stewart 2009; Ying  2020; Zhuo 2021).

There are six ongoing RCTs (IRCT201510137612N2; IRCT20170731035424N2; IRCT20200613047757N1; Kim 2020; Kim 2020; NCT03827109; RBR‐79dn4k).

### Risk of bias in included studies

Below we present the results of our risk of bias assessment (Figure 2; Figure 3). Further details can be found in the risk of bias tables (beneath Characteristics of included studies tables).

**2:**
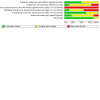


**3:**
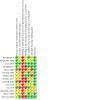


#### Allocation

Randomisation was described clearly in seven studies (Berding 2017; Borgaonkar 2002; Cross 2019; De Jong 2017; Jaghult 2007; Oxelmark 2007; Vaz 2019), which we rated at low for risk of bias, and was not sufficiently described in the other seven studies ( Kennedy 2002; Moreau 2021; Nikolaus 2017; ; Uran 2019; Walkiewicz 2011; Waters 2005; Weizman 2021), which we rated unclear for risk of bias.

We rated two studies at low risk from allocation concealment (Cross 2019; Weizman 2021), as the method of random allocation of participants to intervention and control groups and allocation concealment was described or the risk was low due to cluster randomisation. We rated nine studies at unclear risk of allocation concealment (Berding 2017; Borgaonkar 2002; De Jong 2017; Kennedy 2002; Moreau 2021; Nikolaus 2017; Uran 2019; Walkiewicz 2011; Waters 2005), as they did not provide enough information about their selection and allocation concealment process. Three studies had no allocation concealment and were judged to be at high risk (Jaghult 2007; Oxelmark 2007; Vaz 2019).

#### Blinding

All studies were rated as high in performance bias, as the interventions they studied could not be blinded for both participants and personnel.

Detection bias was rated as low in two studies that mentioned assessors being blinded (Cross 2019; Moreau 2021), unclear in six studies that did not provide enough information for a judgement (Berding 2017; Borgaonkar 2002; Kennedy 2002; Uran 2019; Walkiewicz 2011; Waters 2005), and high in six that confirmed or mentioned that the assessors were not blinded (De Jong 2017; Jaghult 2007; Nikolaus 2017; Oxelmark 2007;Vaz 2019; Weizman 2021).

#### Incomplete outcome data

We judged attrition bias as low in nine studies that provided enough information for judgement (Berding 2017; Cross 2019; De Jong 2017; Kennedy 2002; Moreau 2021; Oxelmark 2007; Vaz 2019; Waters 2005; Weizman 2021). The rest of the studies we rated at unclear risk (Borgaonkar 2002; Jaghult 2007; Nikolaus 2017; Uran 2019; Walkiewicz 2011).

#### Selective reporting

We rated reporting bias as low in three studies that reported all outcomes they had set out to report either in their protocols or trial registrations (Cross 2019; De Jong 2017; Weizman 2021). We rated nine studies at unclear risk (Berding 2017; Borgaonkar 2002; Kennedy 2002; ;Nikolaus 2017Oxelmark 2007; Uran 2019; Vaz 2019; Walkiewicz 2011; Waters 2005), and two at high risk (Jaghult 2007; Moreau 2021).

#### Other potential sources of bias

We rated 12 studies as low risk for other potential sources of bias (Berding 2017; Borgaonkar 2002; Cross 2019; De Jong 2017; Jaghult 2007; Kennedy 2002; Moreau 2021; Nikolaus 2017; Oxelmark 2007; Uran 2019; Waters 2005; Weizman 2021). We rated two studies at unclear risk due to lack of information (Vaz 2019; Walkiewicz 2011).

### Effects of interventions

See: Table 1; Table 2; Table 3

A summary of primary and secondary outcome data can be found in Table 7 and Table 8 respectively. Any planned subgroup and sensitivity analyses that were not carried out because of a lack of data are mentioned in Differences between protocol and review.

**4:** Primary outcome data

**Study ID**	**Disease activity at study end**	**Flare‐ups or relapse**	**Quality of life at study end**
Berding 2017	Perceived disease activity measured using the German Inflammatory Bowel Disease Activity Index (GIBDI) Mean (SD) 2 weeks post intervention: IG: 2.89 (2.36) CG: 3.64 (2.28) 3 months post intervention: IG 3.04 (2.77) CG 3.76 (2.53)	NR	Measured using the SF‐12 questionnaire Physical HRQoL mean (SD) : 2 weeks post‐intervention: NR 3 months post‐intervention: IG: 47.62 (9.08); CG: 46.60 (9.16) Mental HRQoL mean (SD): 2 weeks post‐intervention: NR 3 months post‐intervention: IG: 46.41 (11.00); CG: 42.70 (10.89)
Borgaonkar 2002	NR	NR	IBDQ (total) mean (SD): IG 167.8 (39.9) CG 162.6 (32.4) IBDQ (mean score/question): (range 1–7) IG: 5.26 (1.2) CG: 5.1 (1.0) (the paper also provides results for the 4 items that comprise this questionnaire) QuICC (total) mean (SD): IG: 87.0 (20.61) CG: 85.7 (19.83) QuICC (mean score/question): (range 1–5) IG: 2.4 (0.57) CG: 2.3 (0.54)
Cross 2019	To assess disease activity for participants with CD, the HBI was used and the SCCAI was used to assess disease activity for patients with UC/indeterminate colitis HBI scores at study end, mean (SD): IG1 (TELE‐IBD EOW): 4.2 (3.9) IG2 (TELE‐IBD W): 3.2 (3.4) CG: 3.7 (3.6) SCCAI scores at study end, mean (SD): IG1 (TELE‐IBD EOW): 1.7 (1.9) IG2 (TELE‐IBD W): 2.0 (1.8) CG: 1.4 (1.4)	NR	Disease‐specific QOL was assessed with the IBD Questionnaire (IBDQ). IBDQ scores at study end, mean (SD): IG1 (TELE‐IBD EOW): 181.5 (28.2) IG2 (TELE‐IBD W): 179.2 (32.8) CG: 179.3 (28.2)
De Jong 2017	NR	Mean number of flare‐ups (SD): IG: 0.19 (0.42) CG: 0.19 (0.44)	Short Inflammatory Bowel Disease Questionnaire (SIBDQ) scores at 12 months IG: N = 340, with mean score (SD) 54.44 (9.05) CG: N = 331, with mean score (SD) 53.71 (9.87)
Jaghult 2007	NR	NR	Mean score at 6 months, no SDs given IBDQ: IG 57.85; CG 55.58 IBDQ1: bowel symptoms IG 19.48; CG 19.13 IBDQ2: systemic symptoms IG 11.65; CG 10.55 IBDQ3: social functions IG 6.31; CG 6.13 IBDQ4: emotional functions IG 20.40; CG 19.77 Rating Form of IBD Patient Concerns (RFIPC), median sum score IG 34.75 (25.96); CG 32.14 (21.44) (source material not clear about whether the numbers in brackets are SDs)
Kennedy 2002	NR	Mean number of reported relapses during the trial year: IG: 1.8 (2.2) CG: 2.2 (2.5) Relapses intraclass correlation coefficient (ICC) = 0.054 design effect for clustering: IG: 1.4 CG: 1.5 Effective sample size: IG: 85 CG: 81 Effective sample size after dropouts: IG: 50 CG: 63	IBDQ questionnaire score at study end: IG: mean (SD) 172.3 (36.6) CG: mean (SD) 167.7 (37.5) IBDQ ICC = 0.033 Design effect for clustering: IG: 1.3 CG: 1.3 Effective sample size IG: 92 CG: 93 Effective sample size after dropouts IG: 54 CG: 72
Moreau 2021	NR	NR	QOL measured using the SIBDQ Odds ratio (95% CI) 1.02 (1.01–1.03)
Nikolaus 2017	Authors stated disease activity as an outcome and that they measured it using the CAI, however the data were not presented	Acute relapse defined as CAI ≥ 9 IG: 9 CG: 10	NR
Oxelmark 2007	NR	IG: 1 CG: 0	Mean score (SD) IBDQ at 6 months IG: 175.7 (35.0) CG: 187.9 (27.7) IBDQ at 12 months IG: 171.8 (28.2) CG: 173.7 (28.2) (The paper also provided results for the 4 items that comprise this questionnaire)
Uran 2019	Number of participants at 8 weeks: IG (web‐based education): UC: remission 8, mild disease 6, severe disease 2, very severe disease 0 CD: remission 5, mild disease 7, severe disease 2, very severe disease 0 CG (standard education): UC: remission 10, mild disease 4, severe disease 1, very severe disease 1 CD: remission 10, mild disease 3, severe disease 1, very severe disease 0	NR	IBD Quality of Life Scale (IBDQ) mean (SD), at 8 weeks IG (web‐based education): 156.53 (30.97) CG: 155.63 (34.30)
Vaz 2019	NR	All participants remained in remission throughout the study	NR
Walkiewicz 2011	NR	NR	NR
Waters 2005	NR	NR	Raw results not provided. Author stated, "No difference was found for IBDQ total scores between groups at baseline, T2 or T3." "No differences were found between the education and control groups for mean total RFIPC scores over the course of the study"
Weizman 2021	NR	NR	NR

**CAI:** Colitis Activity Index; **CG:** control group; **HBI:** Harvey‐Bradshaw Index for Crohn's disease; **HRQoL:** health‐related quality of life; **IBD:** inflammatory bowel disease; **IBDQ:** Inflammatory Bowel Disease Questionnaire; **IG:** Intervention group; **NR:** not reported; **QOL:** quality of life; **QuICC:** Quality Index in Crohn’s and Colitis; **RFIPC:** Rating Form of IBD Patient Concerns; **SCCAI:** Simple Clinical Colitis Activity Index; **SD:** standard deviation; **SIBDQ:** Short Inflammatory Bowel Disease Questionnaire; **TELE‐IBD W:** group that received a telemedicine message every week; **TELE‐IBD EOW:** group that received a telemedicine message every other week; **UC:** ulcerative colitis

**5:** Secondary outcome data

**Study ID**	**Number of episodes accessing health care (outpatient, remote or inpatient)**	**Change in disease activity**	**Change in quality of life**	**Medication adherence**	**Patient knowledge and/or skill**	**Total adverse effects (serious and minor)**	**Withdrawals due to adverse events**
Berding 2017	NR	NR	NR	NR	Self‐reported using a 5‐point Likert scale (high score = better result) Mean (SD): **Medical knowledge:** IG: At 2 weeks: 4.23 (0.48) At 3 months: 4.05 (0.41) CG: At 2 weeks: 3.44 (0.65) At 3 months: 3.42 (0.71) **Psychological knowledge** IG: 2 weeks: 3.81 (0.72) At 3 months: 3.65 (0.67) CG: 2 weeks: 2.99 (0.70) At 3 months: 2.98 (0.74)	NR	NR
Borgaonkar 2002	NR	NR	Mean (SD) IBDQ IG: −0.17 (0.49); CG: 0.28 (0.62) (high score = better result) QuICC IG: −0.05 (0.28) CG: −0.01 (0.25) (low score = better result)	NR	NR	NR	NR
Cross 2019	Hospitalisations, surgery, emergency department and office visits, procedures, intravenous therapeutics, and telephone and electronic encounters for one year before and after randomisation were extracted from participants' electronic medical records. Total encounters are reported as rates, adjusted for 100 participants per year IG1 (TELE‐IBD EOW): 2235 IG2 (TELE‐IBD W): 1935 CG: 2099 IBD‐related hospitalisations IG1 (TELE‐IBD EOW): 14.4 IG2 (TELE‐IBD W): 9.8 CG: 16.4 Non‐IBD‐related hospitalisations IG1 (TELE‐IBD EOW): 0.9 IG2 (TELE‐IBD W): 2.7 CG: 11.2 Non‐invasive diagnostic tests IG1 (TELE‐IBD EOW): NR IG2 (TELE‐IBD W): 86.6 CG: 112.9 Electronic encounters IG1 (TELE‐IBD EOW): NR IG2 (TELE‐IBD W): 238.4 CG: 250.9 Telephone encounters IG1 (TELE‐IBD EOW): 988.3 IG2 (TELE‐IBD W): 953.6 CG: 900.9	NR	NR	NR	Because participants without a completed CCKNOW survey at baseline and 12 months were excluded, the authors assessed a total of 219 patients for this outcome. When analysing only the 219 patients with CCKNOW scores at baseline and the 12‐month visit, there were significant differences in age, race and disease activity among the arms. CCKNOW mean difference from baseline (mean, SD) (positive numbers = improvement) IG1 (TELE‐IBD EOW): +2.4 IG2 (TELE‐IBD W): +2.0 CG: +1.8 SDs requested but not provided	NR	IG1 (TELE‐IBD EOW): 1 (breast cancer) IG2 (TELE‐IBD W): 2 (needed surgery) CG: 0 (Information provided in author correspondence)
De Jong 2017	Mean number of hospital admissions (SD): IG: 0.05 (0.28) CG: 0.10 (0.43) Mean number of emergency visits (SD): IG: 0.07 (0.35) CG: 0.10 (0.54)	NR	NR	Medication adherence measured using the Morisky Medication Adherence Scale At 12 months: IG: (n = 340) with mean (SD) 7.01 (1.40); CG: (n = 331) with mean (SD) 6.77 (1.61)	Self‐reported knowledge of IBD measured on a visual analogue scale (0–10; higher score indicates better knowledge) At 12 months, mean (SD): Knowledge of IBD: IG:8.17 (1.16) CG: 7.84 (1.47) Knowledge of medication: IG: 7.75 (1.58) CG: 7.58 (1.51)	No adverse events related to use of the telemedicine intervention occurred	No adverse events related to use of the telemedicine intervention occurred
Jaghult 2007	NR	NR	NR	NR	NR	0 (information provided by the authors)	0 (information provided by the authors)
Kennedy 2002	Mean (SD) number of kept hospital appointments: IG: 1.9 (2.2) CG: 3.0 (2.5) Reported number of participants who did not attend appointments: IG 8% of 274 = 22, + 5 withdrawals CG 12.1% of 364 = 44, + 38 withdrawals Number of hospital appointments ICC: 0.109	NR	NR	NR	NR	NR	NR
Moreau 2021	Mentioned as an outcome but no data reported, only that no significant difference was noted.	NR	NR	Measured using the adherence score which evaluated treatment observance Odds ratio (95% CI) 1.05 (0.91–1.21)	Measured on the ECIPE score. An improvement in patients' skills was defined by an increase of the ECIPE score of more than 20%, from baseline to 6 months. IG (n = 61): 45.9% CG (n = 31): 25% Per protocol ECIPE scores, median (range) Baseline: CG (n = 129): 19 (14‐23) IG (n = 132): 19 (15‐24) 6 months: CG (n = 117): 20 (16‐25) IG (n = 105): 26 (22‐30)	NR	NR
Nikolaus 2017	NR	NR	NR	Non‐adherence rate measured using the Morisky Medication Adherence Scale (MMAS): IG: 52.4% of 126 = 66 participants CG: 52.5% of 122 = 64 participants	NR	NR	NR
Oxelmark 2007	NR	NR	NR	NR	NR	0 (information provided by the authors)	0 (information provided by the authors)
Uran 2019	NR	NR	NR	NR	NR	NR	NR
Vaz 2019	NR	NR	NR	Adherence was calculated by averaging adherence rates (actual number of openings recorded with the MedMinder system divided by expected number of openings based on prescribed regimen) for each adherence period (i.e. 4‐week run in and 4‐week post‐randomisation) for the IG and CG Difference in average adherence rates (pre‐ and post‐randomisation): Mean (SD) IG: 0.36 (10.28) CG: −15.3 (25.34)	Measured using the IBD Knowledge Inventory Device (IBD‐KID) **Baseline total scores** CG: 12.25 (3.30) IG: 11.40 (2.19) **NR at 4 weeks** **Mean (SD) rank scores at baseline** Gastro‐intestinal anatomy: IG: 1.00 (0.71) CG: 1.5 (0.58) General IBD knowledge: IG: 8.00 (2.12) CG: 7.75 (2.1) Medications: IG: 1.4 (0.55) CG: 2.5 (0.58) Nutrition: IG: 1.00 (1.00) CG: 0.50 (1.00) **Mean rank scores at 4 weeks** GI anatomy: IG: 5.8 CG: 4.0 General IBD knowledge (SD not available): IG: 5.6 CG: 4.3 Medications: IG: 6.1 CG: 3.6 Nutrition: IG: 4.2 CG: 6.0	0 (information provided by the authors)	0 (information provided by the authors)
Walkiewicz 2011	NR	NR	NR	NR	Knowledge was assessed using a modified version of the Crohn's & Colitis Foundation of America (CCFA) Knowledge Score (I‐M‐AWARE) The mean pre‐intervention score was 48.7% (range 13.4% to 82.4%). Post‐intervention the mean score on the assessment was 55.6% (range 35.0% to 95.6%). Not reported per intervention group. (High score = better result) Scores for groups NR.	NR	NR
Waters 2005	Rate of health care use measured at 8 weeks post‐education IG: M = 0.63 CG: M = 0.95 No variance reported	NR	NR	Medication adherence was assessed by three methods: survey at baseline; a set of questions on the Patient Satisfaction Questionnaire; and participant self‐report 166 incidents of missed medications with a mean of 2.31 incidents per participant were reported. Mean number of missed medication during the study: IG: 0.91 CG: 3.43 IG rate of non‐adherence: Immediately after intervention: median = 0.32 8 weeks after intervention: median = 0.25	Mean (SD) T2 = Immediately post‐education T3 = 8 weeks post education **Knowledge Questionnaire** IG: T1: 17.13 (7.00) T2:27.77 (3.23) T3: 27.19 (3.03) CG: T1: 17.24 (5.81) T2: 20.84 (6.34) T3: 21.47 (6.81) **CCKNOW** IG: T1: 11.58 (5.64) T2:19.29 (3.30) T3: 19.52 (2.55) CG: T1: 9.79 (4.94) T2:13.34 (5.66) T3:13.84 (4.86) **Perceived knowledge** (no values given, approximation from figure) IG: 5.5 T2: 7.8 T3: 7.6 CG: 5.5 T2: 6 T3: 6.2	NR	NR
Weizman 2021	NR	NR	NR	NR	NR	NR	NR

**CCKNOW:** Chron's and Colitis Knowledge questionnaire; **CG:** control group; **ECIPE:** Étude randomisée et contrôlée évaluant l'impact du programme d'éducation; **HBI:** Harvey‐Bradshaw Index for Crohn's disease; **HRQoL:** health‐related quality of life; **IBD:** inflammatory bowel disease; **IBDQ:** Inflammatory Bowel Disease Questionnaire; **ICC:** intraclass correlation coefficient IG**:** intervention group; **QuICC:** Quality Index in Crohn’s and Colitis; **NR:** not reported; **SCCAI:** Simple Clinical Colitis Activity Index; **SD:** standard deviation; **TELE‐IBD W:** group that received a telemedicine message every week; **TELE‐IBD EOW:** group that received a telemedicine message every other week; **UC:** ulcerative colitis

#### 1. Patient education and standard care versus standard care

Thirteen studies compared patient education interventions against no intervention (Berding 2017, Borgaonkar 2002; Cross 2019; De Jong 2017; Jaghult 2007; Kennedy 2002; Moreau 2021; Nikolaus 2017; Oxelmark 2007; Vaz 2019; Walkiewicz 2011; Waters 2005; Weizman 2021).

##### Primary outcomes

###### Disease activity at study end

Two of the studies that reported this outcome provided continuous data that we could use for a meta‐analysis (Berding 2017; Cross 2019). There was no clear difference in disease activity when patient education (n = 277) combined with standard care was compared to standard care (n = 202). Patient education combined with standard care is probably equivalent to standard care in reducing disease activity in patients with IBD (standardised mean difference (SMD ‐0.03, 95% confidence interval (CI) ‐0.25 to 0.20). The certainty of the evidence was moderate due to concerns with risk of bias (Analysis 1.1; Table 1).

A fixed‐effect sensitivity analysis had similar results (Analysis 1.2).

Nikolaus 2017 mentioned disease activity as an outcome, but did not present any results.

###### Flare‐ups or relapse

Two of the studies that reported this outcome reported it as a continuous outcome (De Jong 2017; Kennedy 2002), and three reported it as a dichotomous outcome (Nikolaus 2017; Oxelmark 2007; Vaz 2019).

For the continuous data meta‐analysis, there was no clear difference for flare‐ups or relapse when patient education (n = 515) combined with standard care was compared to standard care (n = 507), as a continuous outcome. Patient education combined with standard care is probably equivalent to standard care in reducing flare‐ups or relapse in patients with IBD (mean difference (MD) ‐0.00, 95% CI ‐0.06 to 0.05). The certainty of the evidence was moderate due to concerns with risk of bias (Analysis 1.3; Table 1).

A fixed‐effect sensitivity analysis had similar results (Analysis 1.4).

From the dichotomous data, 10 participants experienced relapse in the patient education combined with standard care group (n = 157) and 10 participants experienced relapse in the standard care group (n = 150). The evidence is very uncertain on whether patient education combined with standard care is different to standard care in reducing flare‐ups or relapse in patients with IBD (RR 0.94, 95% CI 0.41 to 2.18). The certainty of the evidence was very low due to serious concerns with risk of bias and imprecision (Analysis 1.5; Table 1).

A fixed‐effect sensitivity analysis had similar results (Analysis 1.6).

Oxelmark 2007 mentioned that one participant relapsed during their study but did not clarify to which group they belonged.

###### Quality of life at study end

Six of the studies that reported this outcome provided continuous data that we could use for a meta‐analysis (Berding 2017; Borgaonkar 2002; Cross 2019; De Jong 2017; Kennedy 2002; Oxelmark 2007).

There was no clear difference in quality of life when patient education combined with standard care (n = 721) was compared to standard care (n = 643). Patient education combined with standard care is probably equivalent to standard care in improving quality of life in patients with IBD (SMD 0.08, 95% CI ‐0.03 to 0.18). The certainty of the evidence was moderate due to concerns with risk of bias (Analysis 1.7; Table 1).

A fixed‐effect sensitivity analysis had similar results (Analysis 1.8).

We conducted a sensitivity analysis excluding five studies at high risk of bias (Berding 2017; Borgaonkar 2002; De Jong 2017; Kennedy 2002; Oxelmark 2007). There was no clear difference in quality of life when patient education combined with standard care (n = 193) was compared to standard care (n = 107). Patient education combined with standard care is probably equivalent to standard care in improving quality of life in patients with IBD (MD 1.11, 95% CI ‐5.74 to 7.97). The certainty of the evidence was moderate due to imprecision (Analysis 1.9).

We conducted a sensitivity analysis excluding one cluster RCT (Kennedy 2002). There was no clear difference in quality of life when patient education combined with standard care (n = 667) was compared to standard care (n = 571). Patient education combined with standard care is probably equivalent to standard care in improving quality of life in patients with IBD (SMD 0.07, 95% CI ‐0.04 to 0.18). The certainty of the evidence was moderate due to concerns with risk of bias (Analysis 1.10).

We conducted a further sensitivity analysis including only the studies that used the full IBDQ (high score = better result) and as such allowed the use of the mean difference (MD) (Borgaonkar 2002; Cross 2019; Kennedy 2002; Oxelmark 2007). There was no clear difference in quality of life when patient education combined with standard care (n = 297) was compared to standard care (n = 217). Patient education combined with standard care may be equivalent to standard care in improving quality of life in patients with IBD (MD 1.82, 95% CI ‐3.72 to 7.36). The certainty of the evidence was low due to concerns with risk of bias and imprecision (Analysis 1.11).

Berding 2017 also measured mental quality of life, in addition to the physical quality of life that was included in the meta‐analysis. The intervention group had a reported score of 46.41 (11.00) and the control group a score of 42.70 (10.89) at 3 months from study end (high score = better result). Borgaonkar 2002 measured quality of life using the QuICC (low score = better result), in addition to the IBDQ that was used in the above meta‐analysis. The intervention group had a reported score of 87.0 (20.61) and the control group a score of 85.7 (19.83) at study end. Jaghult 2007 used the IBDQ and provided mean scores with variance values. The intervention group had a reported score of 57.85 and the control group a score of 55.58 at study end (high score = better result). Moreau 2021 and Waters 2005 did not provide the raw mean and variance scores per group at study end, only presenting the results of their own analysis.

##### Secondary outcomes

###### Number of episodes of accessing health care

In Cross 2019, hospitalisations, surgery, emergency department and office visits, procedures, intravenous therapeutics, and telephone and electronic encounters were extracted from the electronic medical record (EMR) for one year before and after randomisation, and encounters were reported as rates, adjusted for 100 participants per year. The intervention group that received a telemedicine message every other week (IG1 (TELE‐IBD EOW)) had 2235 total encounters, the intervention group that received a telemedicine message every week (IG2 (TELE‐IBD W)) had 1935, and the control group had 2099 (the data on the specific types of encounters are presented in Table 8). De Jong 2017 reported mean numbers of hospital admissions, which were 0.05 (SD 0.28) for the intervention group and 0.10 (SD 0.43) for the control group; and mean numbers of emergency visits, which were 0.07 (SD 0.35) for the intervention group and 0.10 (SD 0.54) for the control group. Kennedy 2002 reported mean number of kept hospital appointments as 1.9 (SD 2.2) for the intervention group and 3.0 (SD 2.5) for the control group, as well as number of participants who did not attend appointments as 22/279 for the intervention group and 44/403 for the control group. Waters 2005 reported rate of health care use as a mean of 0.63 for the intervention group and 0.95 for the control group without providing variance values. Moreau 2021 mentioned it as an outcome, but did not report data.

###### Change in disease activity

This outcome was not reported in any of the studies.

###### Change in quality of life

This outcome was only reported in Borgaonkar 2002. The mean difference in the intervention group was −0.17 (SD 0.49) and in the control group 0.28 (SD 0.62) for the IBDQ and −0.05 (SD 0.28) and −0.01 (SD 0.25), respectively, for the QuICC.

###### Medication adherence

De Jong 2017 reported medication adherence as a mean of 7.01 (SD 1.40) for the intervention group and 6.77 (SD 1.61) for the control group. Nikolaus 2017 reported 66/126 and 64/122 as non‐adherent in the intervention and control groups, respectively. In Vaz 2019, difference in average adherence rates pre‐ and post‐randomisation was +0.36 (SD 10.28) for the intervention group and −15.3 (SD 25.34) for the control group. Waters 2005 reported 166 incidents of missed medications, with a mean of 2.31 incidents per participant, and calculated the mean number of missed medications during the study as 0.91 for the intervention group and 3.43 for the control group.

Moreau 2021 did not provide the raw mean and variance scores per group at study end, instead the authors presented the results of their own analysis.

###### Patient knowledge or skill at study end

In Cross 2019, the mean difference from baseline (no variance provided) was +2.4 in the TELE‐IBD EOW intervention group +2.0 in the TELE‐IBD W intervention group and +1.8 in the control group. Vaz 2019 reported that mean rank scores (no variance provided; high score = better result) at end of study were: 5.8 for the intervention group and 4.0 for the control group for gastrointestinal anatomy; 5.6 and 4.3 for general IBD knowledge; 6.1 and 3.6 for medications; and 4.2 and 6.0 for nutrition. Walkiewicz 2011 reported that post‐intervention the mean score on the assessment was 55.6% (range 35.0% to 95.6%), but did not report results per intervention group. Waters 2005 reported CCKNOW scores of 19.52 (SD 2.55) for the intervention group and 13.84 (SD 4.86) for the control group, and KQ scores of 27.19 (SD 3.03) and 21.47 (SD 6.81) respectively, at study end. In Moreau 2021, an improvement in patients' skills was defined by an increase of the ECIPE score of more than 20%, from baseline to six months. In the intervention group 61 patients achieved that and 31 in the control. Per protocol median ECIPE scores were reported as 26 (range 22‐30) in the intervention group (n = 105) and 20 (range 16‐25) (n = 117) in the control group.

In each of the results in this paragraph, higher scores indicate improvement. Self‐reported medical knowledge was reported in three studies as 4.05 (SD 0.41) for the intervention group and 3.42 (SD 0.71) for the control and psychological knowledge as 3.65 (SD 0.67) and 2.98 (SD 0.74), respectively in Berding 2017. Knowledge of IBD was reported as 8.17 (SD 1.16) for the intervention group and 7.84 (SD 1.47) for the control group, and knowledge of medication as 7.75 (SD 1.58) and 7.58 (SD 1.51), respectively in De Jong 2017. Self‐perceived knowledge was reported as 7.6 for the intervention group and 6.2 for the control group at study end in Waters 2005.

###### Total adverse effects

Jaghult 2007, Oxelmark 2007, De Jong 2017 and Vaz 2019 reported zero total adverse effects in their studies.

###### Withdrawals due to adverse events

The only study that reported withdrawals due to adverse effects was Cross 2019, which reported that in the TELE‐IBD EOW intervention group one participant withdrew due to breast cancer and in the TELE‐IBD intervention group two participants withdrew because they needed surgery. No participants withdrew due to adverse effects from the control group.

#### 2. Web‐based patient education versus other delivery of patient education

Two studies compared delivery methods of patient education in the form of web‐based interventions against other delivery methods (Uran 2019; Walkiewicz 2011).

##### Primary outcomes

Only Uran 2019 reported any of our primary outcomes.

###### Disease activity at study end

Uran 2019 reported numbers of UC and CD participants in remission, or with mild, severe, or very severe disease at study end. For UC participants, 8/16 in the web‐based group and 10/16 in the control education group were in remission, 6/16 and 4/16 had mild disease, 2/16 and 1/16 had severe disease, and 0/16 and 0/16 had very severe disease. For CD participants, 5/14 and 10/14 were in remission, 7/14 and 3/14 had mild disease, 2/14 and 1/14 had severe disease, and 0/14 and 0/14 had very severe disease.

###### Flare‐ups or relapse

This outcome was not reported.

###### Quality of life at study end

Mean quality of life score on the IBDQ for the web‐based group was 156.53 (SD 30.97) and 155.63 (SD 34.30) for the control group (high score = better result).

##### Secondary outcomes

No secondary outcomes were reported except for the limited knowledge score data in Walkiewicz 2011, which we reported above.

#### 3. Weekly educational texts messages versus once every other week educational text messages

Cross 2019 compared frequency of patient education in the form of weekly educational text messages versus once every other week educational text messages (in addition to comparing these interventions to standard care, the results of which we included in the patient education and standard care versus standard care comparison above).

##### Primary outcomes

###### Disease activity at study end

Mean disease activity for the TELE‐EOW CD participants was 4.2 (SD 3.9) and for the TELE‐W CD participants 3.2 (SD 3.4). Mean disease activity for the TELE‐EOW UC participants was 1.7 (SD 1.9) and for the TELE‐W UC participants was 2.0 (SD 1.8).

###### Flare‐ups or relapse

This outcome was not reported.

###### Quality of life at study end

Mean quality of life scores for the TELE‐EOW participants was 181.5 (SD 28.2) and for the TELE‐W participants was 179.2 (SD 32.8)

##### Secondary outcomes

These have been reported in Comparison 1, patient education and standard care versus standard care.

## Discussion

### Summary of main results

Education is clearly of vital importance within any chronic disease and almost certainly offered to all people affected by the condition in some form. However, this review has investigated the use of education as a specific intervention to enhance outcomes for patients. Given the complexity of educational interventions, there are several ways in which this eclectic mix of packages could be categorised. There were synchronous learning sessions which offered live teaching through a number of methods (Berding 2017; Jaghult 2007; Moreau 2021; Nikolaus 2017; Oxelmark 2007; Vaz 2019; Waters 2005) versus those which offered asynchronous access to learning materials (Borgaonkar 2002; Cross 2019; De Jong 2017; Kennedy 2002; Uran 2019; Walkiewicz 2011; Weizman 2021). There were also materials in either digital forms (Cross 2019; Uran 2019; Walkiewicz 2011; Weizman 2021), or traditional printed educational materials (Borgaonkar 2002; Kennedy 2002; Uran 2019). Most studies compared one of these forms of education to normal care, but descriptions of normal care were limited to a few words and no study defined how much education, whether formally or informally, was offered in these standard care groups.

Reporting of most outcomes in a homogeneous fashion was limited, with quality of life at study end reported most commonly in six of the 14 studies which allowed for meta‐analysis, with all other outcomes reported in a more heterogeneous manner that limited analysis. The analysis found that there was no difference in quality of life in the education group (Analysis 1.7). The poor reporting of other outcome measures severely limited the scope for meta‐analysis and also significantly impacted the certainty of evidence due to the imprecision in other results, and may have contributed to inconsistency. Whilst these judgements are objective and in line with guidance, it is possible that further studies could impact the results.

Since no studies reported knowledge or skill assessments in a manner that allowed meta‐analysis, conclusions cannot be drawn about whether the body of evidence for education in inflammatory bowel disease (IBD) shows that such education can educate people in a measurable way. Similarly, medication adherence was discussed in just five studies and was not reported in a manner that allowed meta‐analysis in any of these studies. Safety was also not reported in most studies, but this may reflect the primary authors' inference that education is unlikely to lead to harm. However, in those that did mention this outcome, no adverse events were reported.

### Overall completeness and applicability of evidence

Despite the issues with heterogeneity of reporting discussed above, efficacy outcomes demonstrate with moderate certainty that there is no benefit to quality of life or disease state from patient education interventions. In these areas, it is questionable whether further research would be beneficial. There are, however, a number of areas where the evidence remains incomplete.

The reporting of the educational interventions themselves is a concern. As shown in Table 6 there was capricious reporting of the details of the education. Only those that used standard educational resources, such as booklets or guidebooks) could be considered reproducible (Borgaonkar 2002; Kennedy 2002. For the other interventions it was unclear what content was delivered to achieve which learning outcomes, which pedagogical techniques were deployed in detail to support dissemination, and with what resources. No details of any underpinning theoretical or conceptual frameworks and not much detail of the resources used were reported.

Unlike pharmacological intervention reviews, readers of this review will not just require information about whether something is effective or safe, but about which specific interventions are effective (Gordon 2016) to offer utility in clinical practice (Daniel 2021). This information is not available for most studies in this review. This is a recognised problem in non‐pharmacological trial reporting, even though there is published guidance for primary study authors to help rectify the issue (Hoffman 2014); this guidance clearly was not employed in the primary studies included in this review. In a recent study, 65% of authors within non‐pharmacological intervention trials forwarded the required information on request (Hoffman 2013). This was not the case in this review, with no authors returning further educational details on request, mirroring our previous experience in Cochrane reviewing (Kew 2017). Future studies must rectify this gap and provide details about interventions and utility, for a more complete evidence base.

The choice of outcomes that were used by primary researchers was also a concern. The primary outcomes in many of these studies, which are mirrored in this review, focused on clinically common and important outcomes within IBD research. Disease activity, change of disease state and quality of life are all vital outcomes. As the evidence from this review suggests that for two of these outcomes there is probably no benefit to education, this clearly challenges the initial assumption that led to a focus on these outcomes. On the surface it appears an entirely appropriate hypothesis that these outcomes should be the focus for educational studies. However, on reflection, if education were to have such an impact, it would raise deep questions about the level of basic medical discussion, consent and information sharing of professionals in standard care. Rather, it is the secondary outcomes of this review that have not been fully addressed by the evidence, and it would appear that in many ways these are not only more likely to be impacted by such interventions, but they would seem to have more utility and relevance to the people and professionals investigating such education effects (Rathert 2013).

Medication adherence is a common issue and enhancing education to improve this by empowering patients to make their own choices proactively would seem a suitable outcome for such interventions, but these data were poorly reported in a heterogenous fashion that did not facilitate any meta‐analysis (Conn 2016). Whilst, in the long run, medication adherence may also impact the previously discussed primary outcomes, this in many ways is indirect and would probably require a far longer follow‐up than any of the included studies had. Attendance at, or need for interventions from primary or secondary care sources also seems a useful focus (Alsayed Hassan 2020). It may not be as simple as reducing these, but rather changing patterns of behaviour. As such, investigators may want to consider not just whether attendance changed, but in what way, and ‐ most importantly ‐ why. Empowering patients to seek support at the times that are most vital to enhance their care is as important as reducing attendance, and so simple quantitative comparisons may not be sufficient for such studies (Sokol 2018). Similarly, quality of life measures overall may not be the best to consider for such studies. The Inflammatory Bowel Disease Questionnaire (IBDQ) was the most reported measure (Guyatt 1989), but most of the items included are clinically and symptom focused, with only two subsets that are potentially relevant (emotional and social activity sets). As data from these subsets were rarely reported, this once again represents a gap in the synthesised evidence, and future researchers may wish to consider separate subset reporting (Riordain 2011).

Standard care was commonly used as the comparison, and was poorly reported in all of these studies, with no study providing clear and concise descriptions of what specific education, in what forms, by which people and at what intervals were offered routinely within it. This information is vital, as it is possible that there are huge differences between this and the interventions. The reverse could also be true, with the same education being offered to both study groups, just in different forms. Without clarity about this issue, the completeness and utility of the evidence is limited.

For our analyses we used study end outcome data and we recognise the variability in the timing of outcome assessment as a limitation. Follow‐ups in IBD interventional studies can vary widely, as this is a chronic remitting and relapsing non‐curable condition, which makes it different to other areas of health care.

We identified six ongoing studies, which appear to have the potential to add to the evidence base. However, it is not clear if these studies will be presented in a way that will address the pervasive issues discussed above.

### Quality of the evidence

There were significant issues related to risk of bias in the studies included in this review. Despite our requests emailed to authors of all included studies, we received little data to change our judgements in these key areas.

Whilst most studies were not blinded for performance or detection bias, this can be seen as acceptable given the context of the review. However, there were issues in all other areas that cannot be similarly accepted.

The reporting of the interventions themselves is a source of potential bias, as it is difficult for readers of the studies to understand what specific intervention was delivered, and this limits consideration in all other areas. As already discussed, this is recognised as a problem within health intervention reporting (Hoffman 2013), and within health education systematic review (Gordon 2016), although it is not explicitly identified when applying GRADE to evidence (Gordon 2020). This is the biggest issue with the evidence base, and it limits the utility of any outcomes, as these interventions cannot be replicated or disseminated.

We downgraded certainty for the outcome of disease activity one level due to issues with risk of bias related to blinding, allocation concealment and randomisation in the two studies that provided data for this outcome.

Flare‐ups as a continuous outcome had the same issues with risk of bias, for which we downgraded the certainty by one level.

We downgraded flare‐ups as a dichotomous outcome by a total of three levels; two levels due to serious issues with risk of bias for the three studies that provided data related to blinding, allocation concealment, randomisation, selective reporting and other sources of bias, as well as one level for imprecision due to limited event numbers.

We downgraded quality of life one level due to concerns with risk of bias related to blinding and allocation concealment.

### Potential biases in the review process

Clinical heterogeneity is a key area of concern in this review. Most studies included patients with both Crohn's Disease (CD) and ulcerative colitis (UC) and at different disease states. It would not have been possible to exclude studies that did not differentiate between CD and UC, as this would have affected the vast majority of studies. Exclusion of these studies would exclude a key source of evidence in this area, but their inclusion clearly introduces a source of bias.

We decided that in order for a study to be included in the review, the educational component had to be the primary focus of the study and not part of a larger package. Our decisions were clearly systematic, but it is possible that we missed relevant studies. It is also possible that education may have been part of a package, but again this was not included in the review.

Missing data or unclear outcome data were ongoing issues we encountered for many studies, which represent ways in which the evidence base is lacking. To deal with this, we made a number of methodological choices which have in turn influenced the findings of the review. We contacted authors for missing data and we used the data for analysis, when provided to us. For analyses using dichotomous data, we used the numbers randomised as denominators. As numerators we used the numbers as reported by the authors for positive outcomes. For negative outcomes we used the plausible worst‐case scenario and added the numbers of dropouts to the numerator, as is normal practice for reviews for IBD, given the chronic nature of the condition and the high rates of adverse events and treatment failures across a patient's journey. For withdrawals due to adverse events specifically, we considered as adverse events all unspecified reasons and all reasons that did not automatically preclude the possibility of an adverse event. For analyses using continuous outcomes, we used the sample numbers as reported by the authors, for each particular continuous outcome. If the sample numbers were not reported, we estimated the sample number based on the attrition percentages reported. For cluster‐trial data we calculated effective sample sizes based on chapter 23 of the *Cochrane Handbook for Systematic Reviews of Interventions* (Higgins 2020).

Finally, there are 20 studies awaiting classification. These represent a mix of studies that are potential inclusions, but that have either not produced an output after trial registration, or published an abstract only that would not allow the study to be included. This large number of studies must be considered as another source of bias.

### Agreements and disagreements with other studies or reviews

This is the first Cochrane Review on this topic, and as far as we can tell no other systematic reviews on the topic exist.

None of the international guidelines for IBD mentions the evidence base in support of, or to propose, any specific educational interventions for people with IBD.

## Authors' conclusions

**Implications for practice**

There is evidence that education is probably of no benefit to disease activity or quality of life when compared with standard care, and may be of no benefit to occurrence of relapse when compared with standard care. However, as there was a paucity of specific information regarding the components included in either education or standard care, the utility of these findings is questionable.

**Implications for research**

Further research to investigate the impact of education on our primary outcomes of disease activity, disease state and quality of life is probably not indicated. This conclusion is not based on the outcomes of the analyses in this review alone, but on consideration of the likely mechanism of action of extra or bespoke inflammatory bowel disease (IBD) education, and indeed the goals of educational interventions for the stakeholders they are likely to impact.

Further research should focus on two key areas. The first is to report details of the educational interventions in a manner that supports transparency, dissemination and replication using existing guidance. The second is to focus on outcomes that educational interventions can be directly targeted to address. These should be informed by direct engagement with stakeholders and people affected by Crohn's disease and ulcerative colitis. Medication adherence and quality of life subsets would be good targets for further work.

Further research on subsets of patients ‐ such as the newly diagnosed, or socially and financially disadvantaged ‐ who may be in greater need of educational support, should also be encouraged.

Within all such studies, reporting in a manner that is consistent with clarity for risk of bias judgements is vital.

## History

Protocol first published: Issue 1, 2021

## Appendices

### Appendix 1. CENTRAL search strategy (Cochrane Library)

#1 ([mh "Inflammatory Bowel Diseases"] or (Inflammatory Bowel Disease* or IBD or Crohn* or Colitis or Enteritis or Proctocolitis or Colorectitis or Ileocolitis):ti,ab) AND ([mh ^"Patient Education as Topic"] or [mh ^"Health Education"] or [mh "Consumer Health Information"] or ((Health NEXT (Education* or Information or Literacy)) or Twitter or Facebook or Instagram or YouTube or Social Media or Multi?medi* or Compact Disk? or Compact Disc? or DVD or Video* or Audio* or Web or Website? or Podcast* or E?mail* or Mail or Mobile Application* or App or Apps or Smartphone? or iPhone* or Handout or Printed or Print or Online or Internet or Booklet* or Poster or Posters or Written Material* or Pamphlet* or Brochure* or Leaflet* or Flyer* or ((Patient* or Consumer*) NEAR (Educat* or Inform* or Literacy or Training))):ti,ab)

in Trials 941

### Appendix 2. MEDLINE search strategy (Ovid)

Database: Ovid MEDLINE(R) ALL 1946 to November 27, 2022

1 exp Inflammatory Bowel Diseases/ or (Inflammatory Bowel Disease* or IBD or Crohn* or Colitis or Enteritis or Proctocolitis or Colorectitis or Ileocolitis).ti,ab. (163153)

2 Patient Education as Topic/ or Health Education/ or exp Consumer Health Information/ or ((Health adj (Education* or Information or Literacy)) or Twitter or Facebook or Instagram or YouTube or Social Media or Multi?medi* or Compact Disk? or Compact Disc? or DVD or Video* or Audio* or Web or Website? or Podcast* or E?mail* or Mail or Mobile Application* or App or Apps or Smartphone? or iPhone* or Handout or Printed or Print or Online or Internet or Booklet* or Poster or Posters or Written Material* or Pamphlet* or Brochure* or Leaflet* or Flyer* or ((Patient* or Consumer*) adj (Educat* or Inform* or Literacy or Training))).ti,ab. (977825)

3 ((randomized controlled trial or controlled clinical trial).pt. or (randomi?ed or placebo or randomly or trial or groups).ab. or drug therapy.fs.) not (exp animals/ not humans.sh.) (4826695)

4 and/1‐3 (1127)

Note: Line 3 is Cochrane Highly Sensitive Search Strategy for identifying randomised trials in MEDLINE: sensitivity‐maximising version (2008 revision)

### Appendix 3. Embase search strategy (Ovid)

Database: Embase <1974 to 2022 November 27>

1 exp *Inflammatory Bowel Disease/ or (Inflammatory Bowel Disease* or IBD or Crohn* or Colitis or Enteritis or Proctocolitis or Colorectitis or Ileocolitis).ti,ab. (240536)

2 *Patient Education/ or *Health Education/ or *Health Literacy/ or *Consumer Health Information/ or ((Health adj (Education* or Information or Literacy)) or Twitter or Facebook or Instagram or YouTube or Social Media or Multi?medi* or Compact Disk? or Compact Disc? or DVD or Video* or Audio* or Web or Website? or Podcast* or E?mail or Mail or Mobile Application* or App or Apps or Smartphone? or iPhone* or Handout or Printed or Print or Online or Internet or Booklet* or Poster or Posters or Written Material? or Pamphlet* or Brochure* or Leaflet* or Flyer* or ((Patient* or Consumer*) adj (Educat* or Inform* or Literacy or Training))).ti,ab. (1199865)

3 Randomized controlled trial/ or Controlled clinical study/ or randomization/ or intermethod comparison/ or double blind procedure/ or human experiment/ or (random$ or placebo or (open adj label) or ((double or single or doubly or singly) adj (blind or blinded or blindly)) or parallel group$1 or crossover or cross over or ((assign$ or match or matched or allocation) adj5 (alternate or group$1 or intervention$1 or patient$1 or subject$1 or participant$1)) or assigned or allocated or (controlled adj7 (study or design or trial)) or volunteer or volunteers).ti,ab. or (compare or compared or comparison or trial).ti. or ((evaluated or evaluate or evaluating or assessed or assess) and (compare or compared or comparing or comparison)).ab. (5982927)

4 (random$ adj sampl$ adj7 ("cross section$" or questionnaire$1 or survey$ or database$1)).ti,ab. not (comparative study/ or controlled study/ or randomi?ed controlled.ti,ab. or randomly assigned.ti,ab.) (9212)

5 Cross‐sectional study/ not (randomized controlled trial/ or controlled clinical study/ or controlled study/ or (randomi?ed controlled or control group$1).ti,ab.) (327953)

6 (((case adj control$) and random$) not randomi?ed controlled).ti,ab. (20471)

7 (Systematic review not (trial or study)).ti. (229188)

8 (nonrandom$ not random$).ti,ab. (18254)

9 ("Random field$" or (random cluster adj3 sampl$)).ti,ab. (4297)

10 (review.ab. and review.pt.) not trial.ti. (1044406)

11 "we searched".ab. and (review.ti. or review.pt.) (44743)

12 ("update review" or (databases adj4 searched)).ab. (55333)

13 (rat or rats or mouse or mice or swine or porcine or murine or sheep or lambs or pigs or piglets or rabbit or rabbits or cat or cats or dog or dogs or cattle or bovine or monkey or monkeys or trout or marmoset$1).ti. and animal experiment/ (1175895)

14 Animal experiment/ not (human experiment/ or human/) (2468723)

15 or/4‐14 (4102891)

16 3 not 15 (5291310)

17 and/1‐2,16 (1861)

18 limit 17 to (conference abstracts or embase) (1770)

### Appendix 4. ClinicalTrials.Gov search strategy

Advanced Search

*Condition or disease*: Inflammatory Bowel Disease OR IBD OR Crohn* OR Colitis OR Enteritis OR Proctocolitis OR Colorectitis OR Ileocolitis

*Other terms*: Randomized

*Study type*: Interventional Studies (Clinical Trials)

*Intervention/treatment*: Education OR Information OR Literacy OR Training

121 Studies found

### Appendix 5. WHO ICTRP search strategy

Advanced Search

Inflammatory Bowel Disease OR IBD OR Crohn* OR Colitis OR Enteritis OR Proctocolitis OR Colorectitis OR Ileocolitis *in the Condition*

Education OR Information OR Literacy OR Training *in the Intervention*

*Recruitment Status is* ALL

87 records for 87 trials found!

## Data and analyses

**Comparison 1:** Patient education and standard care versus standard care

Outcome or subgroup title	No. of studies	No. of participants	Statistical method	Effect size
1.1 Disease activity at study end	2	479	Std. Mean Difference (IV, Random, 95% CI)	‐0.03 [‐0.25, 0.20]
1.1.1 IBD (3 months post intervention)	1	179	Std. Mean Difference (IV, Random, 95% CI)	‐0.26 [‐0.56, 0.03]
1.1.2 CD (TELE‐IBD every other week)	1	104	Std. Mean Difference (IV, Random, 95% CI)	0.13 [‐0.27, 0.54]
1.1.3 CD (TELE‐IBD weekly)	1	99	Std. Mean Difference (IV, Random, 95% CI)	‐0.14 [‐0.55, 0.27]
1.1.4 UC (TELE‐IBD every other week)	1	48	Std. Mean Difference (IV, Random, 95% CI)	0.17 [‐0.42, 0.76]
1.1.5 UC (TELE‐IBD weekly)	1	49	Std. Mean Difference (IV, Random, 95% CI)	0.35 [‐0.23, 0.94]
1.2 Disease activity at study end (fixed‐effect sensitivity analysis)	2	479	Std. Mean Difference (IV, Fixed, 95% CI)	‐0.05 [‐0.24, 0.13]
1.2.1 IBD (3 months post intervention)	1	179	Std. Mean Difference (IV, Fixed, 95% CI)	‐0.26 [‐0.56, 0.03]
1.2.2 CD (TELE‐IBD every other week)	1	104	Std. Mean Difference (IV, Fixed, 95% CI)	0.13 [‐0.27, 0.54]
1.2.3 CD (TELE‐IBD weekly)	1	99	Std. Mean Difference (IV, Fixed, 95% CI)	‐0.14 [‐0.55, 0.27]
1.2.4 UC (TELE‐IBD every other week)	1	48	Std. Mean Difference (IV, Fixed, 95% CI)	0.17 [‐0.42, 0.76]
1.2.5 UC (TELE‐IBD weekly)	1	49	Std. Mean Difference (IV, Fixed, 95% CI)	0.35 [‐0.23, 0.94]
1.3 Flare‐ups or relapse (continuous)	2	1022	Mean Difference (IV, Random, 95% CI)	‐0.00 [‐0.06, 0.05]
1.4 Flare‐ups or relapse (continuous ‐ fixed‐effect sensitivity analysis)	2	1022	Mean Difference (IV, Fixed, 95% CI)	‐0.00 [‐0.06, 0.05]
1.5 Flare‐ups or relapse (dichotomous)	3	307	Risk Ratio (M‐H, Random, 95% CI)	0.94 [0.41, 2.18]
1.6 Flare‐ups or relapse (dichotomous: fixed‐effect sensitivity analysis)	3	307	Risk Ratio (M‐H, Fixed, 95% CI)	0.96 [0.42, 2.20]
1.7 Quality of life at study end	6	1364	Std. Mean Difference (IV, Random, 95% CI)	0.08 [‐0.03, 0.18]
1.8 Quality of life at study end (fixed‐effect sensitivity analysis)	6	1364	Std. Mean Difference (IV, Fixed, 95% CI)	0.08 [‐0.03, 0.18]
1.9 Quality of life at study end: sensitivity analysis for risk of bias	1	300	Mean Difference (IV, Random, 95% CI)	1.11 [‐5.74, 7.97]
1.10 Quality of life at study end: sensitivity analysis excluding cluster‐RCTs	5	1238	Std. Mean Difference (IV, Random, 95% CI)	0.07 [‐0.04, 0.18]
1.11 Quality of life at study end: sensitivity analysis using IBDQ only	4	514	Mean Difference (IV, Random, 95% CI)	1.82 [‐3.72, 7.36]

## Characteristics of studies

### Characteristics of included studies [ordered by study ID]

**Table:** Berding 2017

***Study characteristics***		
Methods	**Study design:** prospective RCT **Study duration:** 2011‐2015 **Setting:** IBD referral centres	
Participants	**State of disease at beginning of study per IG/CG:** inactive or low disease activity **Disease type per IG/CG:** mean (SD) Crohn’s disease: IG 3.1 (1.9); CG 3.8 (2.4) 0.096 Ulcerative colitis: IG 2.9 (3.0) CG 4.1 (3.2) **Inclusion criteria:** patients aged 18 or over with an established diagnosis of IBD **Exclusion:** insufficient language skills, severe vision or hearing impairment, serious physical or psychological comorbidity. Attendance at an IBD education programme up to 6 months before the study. **Age at beginning of study per IG/CG:** Mean (SD) IG: 39.6 (13.2); CG 40.1 (12.3) **Sex per IG/CG:** IG: 33.7% of 86 = 29 men, 57 women CG: 28.4% of 95 = 27 men, 68 women **Disease duration per IC/CG:** mean (SD) years IG: 10.9 (10.8); CG: 9.6 (8.9) **Number randomised per IG/CG:** IG 105; CG 102 **Number reaching end of study per IG/CG:** IG 84; CG 95	
Interventions	**IG:** a 2‐part patient education seminar involving tasks and discussions covering medical information and coping and self‐management skills lasting for 11.5 hours over two days. **CG:** treatment as usual (no education)	
Outcomes	**Duration of follow ‐up**: 3 months **Primary outcomes as defined by study authors:** disease related worries and concerns measured using the Rating Form of the IBD Patient Concerns Questionnaire **Secondary outcomes as defined by study authors:** fear of progression, coping with anxiety, health competencies, HRQoL, symptoms of depression and anxiety, disease‐related knowledge and coping strategies	
Notes	**Funding source:** German Federal Ministry of Education and Research, the German Pension Insurance, the National Association of Statutory Health Insurers and Association of Private Health Insurers. Funding number 01GX1001. **Conflicts of interest:** none	
***Risk of bias***		
**Bias**	**Authors' judgement**	**Support for judgement**
Random sequence generation (selection bias)	Low risk	Computer‐generated numbers used.
Allocation concealment (selection bias)	Unclear risk	Author states "To ensure allocation concealment, central block randomization (ratio 1: 1) was used", but this explanation does not ensure allocation concealment. Authors were contacted, no response was received.
Blinding of participants and personnel (performance bias) All outcomes	High risk	Blinding not possible due to the nature of the intervention (patient education).
Blinding of outcome assessment (detection bias) All outcomes	Unclear risk	No mention of blinding of outcome assessment. Authors were contacted, no response was received.
Incomplete outcome data (attrition bias) All outcomes	Low risk	The intervention group lost 21/105 (attendance issues n =19, did not return questionnaires n = 2) and the wait list group lost 7/102 (did not return questionnaires n=7). In the end, there is no major imbalance, taking into account the nature of the comparison and the outcomes assessed.
Selective reporting (reporting bias)	Unclear risk	No protocol or trial registration. Outcomes presented in the methods section were clearly presented.
Other bias	Low risk	No baseline imbalances, however the baseline characteristics are only presented for the completers and not for all randomised patients who completed the baseline assessment. No other concerns.

**Table:** Borgaonkar 2002

***Study characteristics***		
Methods	**Study design:** prospective RCT **Study duration:** NR **Setting:** ambulatory gastroenterology clinic	
Participants	**State of disease at beginning of study per IG/CG:** IG active/inactive disease: 40% of 34 = 13.6 probably rounded to 14/20 CG active/inactive disease: 48% of 25 = 12/13 **Disease type per IG/CG:** mix IG: CD/UC 18/16 and CG CD/UC: 18/7 **Inclusion criteria:** patients were eligible to participate if their diagnosis had been confirmed by endoscopy, radiography, and/or histologic examination. **Exclusion:** subjects were excluded if they were not fluent in English or had a significant comorbid illness that could potentially impair HRQoL (for example, rheumatoid arthritis) **Age at beginning of study per IG/CG:** mean (SD) IG: 41.5 (11.9); CG: 43 (124.2) **Sex per IG/CG:** N (%) IG: Female 21 (61.8%), Male: 13 (38.2%); CG: Female 12 (48%), Male 13 (52%) **Disease duration per IC/CG:** mean (SD) months IG: 96.4 (85.21); CG: 9.6 (8.9) **Number randomised per IG/CG:** IG: 34; CG: 25 **Number reaching end of study per IG/CG:** IG 30; CG 23	
Interventions	**IG:** information booklets available from the Crohn’s and Colitis Foundation of Canada administered to the IG. **CG:** "usual care".	
Outcomes	**Duration of follow ‐up:** the mean time between enrolment and follow‐up was 27.0 ± 15.6 days for the education group and 22.6 ± 9.3 days for the control group **Primary outcomes as defined by study authors:** HRQoL measured using the Inflammatory Bowel Disease Questionnaire (IBDQ), and the Quality Index in Crohn’s and Colitis (QuICC) **Secondary outcomes as defined by study authors:** NR	
Notes	**Funding source:** NR **Conflicts of interest:** NR	
***Risk of bias***		
**Bias**	**Authors' judgement**	**Support for judgement**
Random sequence generation (selection bias)	Low risk	Computer‐generated
Allocation concealment (selection bias)	Unclear risk	No mention. Authors were contacted, no response was received.
Blinding of participants and personnel (performance bias) All outcomes	High risk	It is not possible to blind participants to the intervention
Blinding of outcome assessment (detection bias) All outcomes	Unclear risk	No mention. Authors were contacted, no response was received.
Incomplete outcome data (attrition bias) All outcomes	Unclear risk	Balanced attrition between groups but no details given about the dropouts. Authors were contacted, no response was received.
Selective reporting (reporting bias)	Unclear risk	No protocol or trial registration. Outcomes mentioned in the methods section were appropriate and reported in the results
Other bias	Low risk	There are no sizeable imbalances between groups. No other concerns.

**Table:** Cross 2019

***Study characteristics***		
Methods	**Study design:** prospective RCT **Study duration:** 2013‐2016 **Setting:** hospitals	
Participants	**State of disease at beginning of study per IG/CG:** IG1 (TELE‐IBD W), IG2 (TELE‐IBD EOW) and CG had a mix of active and inactive IBD patients Baseline HBI IG1 (TELE‐IBD EOW): 5.2 (5) IG2 (TELE‐IBD W): 4.7 (4.1) CG: 4.2 (4.2) **Baseline SCCAI scores** IG1 (TELE‐IBD EOW): 2.9 (3.1) IG2 (TELE‐IBD W): 2.7 (3.1) CG: 2.5 (2.5) **Disease type per IG/CG:** CD participants (n = 236) IG1 (TELE‐IBD EOW): 79 IG2 (TELE‐IBD W): 78 CG: 79 CD participants (n = 112) IG1 (TELE‐IBD EOW): 36 IG2 (TELE‐IBD W): 38 CG: 38 **Inclusion criteria:** Documented IBD on the basis of usual diagnostic criteria Experienced at least one flare of IBD in the 2 years prior to the baseline visit (an increase in IBD symptoms sufficient to warrant a change in medication dose or addition of a medication) >18 years of age Patients in remission secondary to oral steroid use were included. **Exclusion:** inability to speak English, GI surgery in the past, pending or imminent surgery, history of short bowel syndrome, uncontrolled medical or psychiatric disease or pregnancy. **Age at beginning of study per IG/CG:** Mean (SD): IG1: 39.5 (13.4); IG2: 36.9 (11.2); CG: 39.2 (12.1) **Sex per IG/CG:** N (%) of males: IG1: 30 (40%); IG2: 31 (44%); CG: 32 (43%) N (%) of females: IG1: 45 (60%); IG2 39 (56%); CG: 42 (57%) **Disease duration per IC/CG:** mean (SD) years IG: 12.4 (9.7); CG: 11.7 (10.0) **Number randomised per IG/CG:** IG1:115; IG2: 116; CG: 117 **Number reaching end of study per IG/CG:** IG1: 88; IG2: 81; CG: 90	
Interventions	The main intervention was educational text messages with different frequencies; IG1 received a weekly telemedicine message (TELE‐IBD W) IG2 received biweekly telemedicine message (TELE‐IBD EOW) CG: did not receive any educational messages but only received educational materials at routine clinic	
Outcomes	**Duration of follow ‐up:** study visits were conducted at baseline, 6 months then 12 months **Primary outcomes as defined by study authors:** Assessment of disease activity and of quality of life **Secondary outcomes as defined by study authors:** Assessment of utilisation of healthcare resources, patient knowledge, social constraint, self‐efficacy, locus of control, client satisfaction, participant attitudes towards IBD	
Notes	**Funding source:** the Agency for Healthcare Research and Quality (1R01HS018975‐01A1) and the University of Maryland General Clinical Research Centers Program. Ameer Abutaleb and Kenechukwu Chudy‐Onwugaje were supported by T32 training grant DK067872. **Conflicts of interest:** none	
***Risk of bias***		
**Bias**	**Authors' judgement**	**Support for judgement**
Random sequence generation (selection bias)	Low risk	Randomisation order was computer generated. A permuted block randomisation procedure with randomly varied block sizes was used.
Allocation concealment (selection bias)	Low risk	Once completed, the randomisation arm assignments for each of the 4 (UC remission, UC active disease, CD remission, and CD active disease) strata were sent to the Cooperative Studies Program (CSP) Coordinating Center at the Veterans Affairs in Perry Point, MD, and entered into their interactive voice response system. Investigators and research staff remain blinded to the randomisation order. After all responses were entered into the interactive voice response system after informed consent, research staff were alerted to group assignment.
Blinding of participants and personnel (performance bias) All outcomes	High risk	Blinding not possible for this study
Blinding of outcome assessment (detection bias) All outcomes	Low risk	Quote: "Investigators and staff were blinded to the randomization order, but patients, staff, and providers were not masked to group assignment." However, according to the trial registration https://clinicaltrials.gov/ct2/show/NCT01692743, this study is single blinded (outcome assessors) Response from authors: "The research staff was blind to the study group during the outcomes assessment."
Incomplete outcome data (attrition bias) All outcomes	Low risk	Balanced attrition and reasons for withdrawals thoroughly explained by the authors in our correspondence (27 October 2021). 48 patients in the intervention groups discontinued and were accounted for in the published paper, while 42 patients were lost‐to‐follow up in the control group as the author explained to us.
Selective reporting (reporting bias)	Low risk	In the 2015 published protocol for the study, there are more secondary outcomes than the ones reported in the results. Most of them have been reported in three publications referenced in this RCT, including all the ones relevant to this review. The outcomes match with the registration of the trial (NCT01692743)
Other bias	Low risk	No baseline imbalances and no other concerns.

**Table:** De Jong 2017

***Study characteristics***		
Methods	**Study design:** "pragmatic" randomised controlled trial **Study duration:** 9 September 2014 to 18 May 2015 **Setting:** conducted at four hospitals in the Netherlands: two academic hospitals (Maastricht University Medical Centre and Leiden University Medical Centre), and two large, non‐academic regional hospitals (Zuyderland Medical Centre, Sittard and St Antonius Hospital, Nieuwegein).	
Participants	**State of disease at beginning of study per IG/CG:** baseline disease activity: IG: remission 394 (85%), active disease 71 (15%) CG: remission 380 (86%), active disease 64 (14%) **Disease type per IG/CG:** IG: 282 (61%) CD patients, 183 (39%) UC patients CG: 262 (59%) CD patients, 182 (41%) UC patients **Inclusion criteria:** IBD patients, between 18 and 75 years, fulfilling the international diagnostic criteria for inflammatory bowel disease **Exclusion:** all IBD patient who were not able to read or understand the informed consent form, and did not have Internet access by computer, tablet, or Smartphone or patients with a hospital admission within 2 weeks before inclusion were excluded for ethical reasons, because these patients were deemed unable to make an informed decision for participation. Patients with an ileoanal pouch or ileorectal anastomosis were also excluded. **Age at beginning of study per IG/CG:** mean age in years (SD) IG: 44.0 (14.1) CG: 44.1 (14.2) **Sex per IG/CG: IG:** Number of males (%): IG 194 (42%); CG 180 (41%) Number of females (%): IG 271 (58%); CG 264 (59%) **Disease duration per IC/CG:** mean (SD) years IG: 12.8 (10.4); CG: 13.1 (10.8) **Number randomised per IG/CG:** IG: 465; CG: 444 **Number reaching end of study per IG/CG:** IG: 438; CG: 443	
Interventions	**Telemedicine or myIBDcoach in the intervention group (IG) versus standard care in the control group (CG)** **IG:** participants received instructions, a username, and a password for a telemedicine system (myIBDcoach), which included intensified monitoring modules (weekly in case of flare), outpatient visit modules (to prepare for an outpatient visit), e‐learning modules, a personal care plan, and an administrator page used by the healthcare provider (i.e. gastroenterologist or nurse). Participants used the system for 12 months and were instructed to plan at least one routine outpatient visit per year. Additional follow‐up visits were scheduled on the basis of alarm symptoms recognised by the telemedicine system or at the requests of individual patients. **CG:** participants continued their routine follow‐up visits following the local protocol, with the opportunity to schedule an extra visit if symptoms relapsed. At baseline and after 12 months all participants received a paper questionnaire regarding perceived quality of care, medication adherence, quality of life, self‐efficacy, disease‐related and medication‐related knowledge, and smoking behaviour.	
Outcomes	**Primary outcomes:** number of outpatient visits and patient‐reported quality of care. The number of outpatient visits and telephone consultations with gastroenterologists and nurses during the 12‐month period were retrieved from patients’ electronic medical records. **Secondary outcomes:** adherence to treatment, quality of life, self‐efficacy, disease‐related and medication‐related knowledge, smoking behaviour, and disease outcomes.	
Notes	**Funding:** supported by an academic incentive fund of the Maastricht University Medical Centre (31962340B). MyIBDcoach was developed by Sananet BV using an unrestricted grant from Ferring. The funding source had no role in study design, data collection, data analysis, data interpretation, or writing of the report. The corresponding author had full access to all the data in the study and had final responsibility for the decision to submit for publication. **Declaration of interests:** MJdJ reports non‐financial support from Merck Sharpe & Dohme, outside the submitted work. AEvdM‐dJ reports grants and non‐financial support from Takeda, personal fees from AbbVie, and non‐financial support from Tramedico, all outside the submitted work. AAvB reports personal fees from AbbVie, MSD, Ferring, Tramedico, Takeda, Pfizer, and Janssen, all outside the submitted work. GD reports speaker’s fees from Shire, AbbVie, and Takeda, and a grant for investigator‐initiated research from Takeda, all outside the submitted work. AAM reports grants from Grünenthal, Zon MW GGG (government), Will Pharma, BioActor, Pentax Europe, Falk Pharma, and Almiral Pharma, all outside the submitted work. AB received research grants to her department from AbbVie, Amgen, and Merck, and advisory board honoraria from Janssen and Sandoz, all unrelated to the current work. MJP reports personal fees from AbbVie, Ferring, Janssen, and Takeda, and grants from Falk, all outside the submitted work. All other authors declare no competing interests.	
***Risk of bias***		
**Bias**	**Authors' judgement**	**Support for judgement**
Random sequence generation (selection bias)	Low risk	Using ALEA Screening and Enrolment Application Software where patients were randomly assigned (1:1) to care via the telemedicine system (intervention) or standard care (control).
Allocation concealment (selection bias)	Unclear risk	No mention of allocation concealment. We contacted the author, and they responded "Participants, health‐care providers, and staff who assessed outcome measures were not masked to treatment allocation"
Blinding of participants and personnel (performance bias) All outcomes	High risk	Participants, healthcare providers, and staff who assessed outcome measures were not masked to treatment allocation
Blinding of outcome assessment (detection bias) All outcomes	High risk	No mention of any outcome assessment blinding.
Incomplete outcome data (attrition bias) All outcomes	Low risk	The telemedicine follow‐up group lost 27/465 and the routine follow‐up group lost 1/444. There is a detailed explanation of the reasons for patients withdrawing and we think the reasons are appropriate given what each group received and there's no major imbalance that will affect the outcomes.
Selective reporting (reporting bias)	Low risk	This trial was registered at ClinicalTrials.gov (NCT02173002) and appropriate outcomes reported as per method section.
Other bias	Low risk	No baseline imbalances and no other concerns.

**Table:** Jaghult 2007

***Study characteristics***		
Methods	**Study design:** prospective RCT **Study duration:** NR **Setting:** IBD Clinic	
Participants	**State of disease at beginning of study per IG/CG:** all participants had inactive disease **Disease type per IG/CG:** CD: 26/16; UC: 26/16 **Inclusion criteria:** people with confirmed diagnosis of CD or UC of less than 2 years’ duration and in clinical remission, and who visited the IBD clinic between November 2002 and November 2004. Patient also had to have a good understanding of the Swedish language and be able to complete a questionnaire. **Exclusion:** people with any other chronic disease **Age at beginning of study per IG/CG:** mean (range) IG: 41.71 (17‐75); CG: 39.44 (18‐73) **Sex per IG/CG:** Number of male/female IG: 22/30; CG: 19/13 **Disease duration per IC/CG:** mean (range) years IG: 1.60 (1‐2); CG: 1.59 (1‐2) **Number randomised per IG/CG:** IG: 55; CG: 44 **Number reaching end of study per IG/CG:** IG: 52; CG: 32	
Interventions	**IG:** participants attended a multi professional group‐based education programme with CD patients in separate groups from UC patients. **CG:** participants received regular information during visits to the IBD clinic.	
Outcomes	**Duration of follow ‐up**: 6 months **Primary outcomes as defined by study authors:** HRQoL measured using the Health Index (HI), IBDQ and the RFIPC **Secondary outcomes as defined by study authors:** coping capacity measured using the Sense of Coherence (SOC) questionnaire.	
Notes	**Funding source:** NR **Conflicts of interest:** NR	
***Risk of bias***		
**Bias**	**Authors' judgement**	**Support for judgement**
Random sequence generation (selection bias)	Low risk	Author response: computer‐generated randomisation
Allocation concealment (selection bias)	High risk	Author response: "patients were randomized to an intervention or control group, using allocation concealment". However, after contact, the author stated that they were "the only one that had access to the allocation list, and it was locked in a cabinet."
Blinding of participants and personnel (performance bias) All outcomes	High risk	It is not possible to blind the patients to the intervention (education)
Blinding of outcome assessment (detection bias) All outcomes	High risk	Author response: Unblinded for outcome assessors
Incomplete outcome data (attrition bias) All outcomes	Unclear risk	Author response about reasons for drop‐outs: For the control group 6 patients dropped out and gave the reason that they wanted to be in the intervention group. In the intervention group there were 3 dropouts without giving an explanation. This resulted in 55 patients receiving the intervention and 38 receiving the control intervention.
Selective reporting (reporting bias)	High risk	No protocol/trial registration. Only mean of results given with no standard deviation. After contact the author responded that they "did not perform this analysis".
Other bias	Low risk	No baseline imbalances and no other concerns.

**Table:** Kennedy 2002

***Study characteristics***		
Methods	**Study design:** prospective multicentre cluster‐RCT **Study duration:** patients were recruited between July 1999–August 2000, and followed for 12 months. The trial ended 12 months after the last patient entered the study **Setting:** outpatient departments of 19 hospitals	
Participants	**State of disease at beginning of study per IG/CG:** N (%) active: CG: 85 (23.3%); IG: 69 (29.6%) N (%) relapse in past 18 months: CG: 196 (53.7%); IG: 137 (50.7%) N (%) in remission with no flare‐ups in past 18 months: CG: 58 (15.9%); IG: 47 (17.4%) **Disease type per IG/CG:** mix of UC and CD Disease type N (%): CG: UC: 226 (61.9%); CD: 139 (38.1%) IG: UC: 177 (65.6%); CD: 92 (34.1%) **Inclusion criteria:** established UC or CD, over the age of 16 years, able to write English **Exclusion:** NR **Age at beginning of study per IG/CG:** Mean age (SD) for CG: 46.3 (15.1); IG: 44.4 (14.9) **Sex per IG/CG:** N (%) males in CG: 157 (43%); IG: 112 (41.55) N (%) females in CG: 208 (57%); IG: 158 (58.5) **Disease duration per IC/CG:** Diagnosed in the past year: IG: 15/119 CG: 21/121 Diagnosed over 20 years ago: IG: 14/119 CG: 12/121 **Number randomised per IG/CG:** IG: 9 control sites and 119 participants; CG: 10 control sites and 121 participants **Number reaching end of study per IG/CG:** IG: 70 participants; CG: 94 participants	
Interventions	**IG:** participants received guidebooks for both Crohn's and UC **CG:** standard care which was deemed appropriate by the hospital specialist	
Outcomes	**Duration of follow‐up:** 12 months **Primary outcomes as defined by study authors:** quality of life, health service resource use, and patient satisfaction. **Secondary outcomes as defined by study authors:** enablement/confidence to cope with the condition.	
Notes	**Funding source:** Health Technology Assessment Programme of the UK NHS & Career Scientist Award in Public Health funded by the NHS R&D programme **Conflicts of interest:** none	
***Risk of bias***		
**Bias**	**Authors' judgement**	**Support for judgement**
Random sequence generation (selection bias)	Unclear risk	Cluster randomisation mentioned but no description of the method and no information could be provided by the authors. Author was contacted, no response received.
Allocation concealment (selection bias)	Unclear risk	It is unclear if all clusters were randomised at the same time. We contacted the authors but received no response
Blinding of participants and personnel (performance bias) All outcomes	High risk	No blinding within cluster sites receiving intervention
Blinding of outcome assessment (detection bias) All outcomes	Unclear risk	No mention of any outcome assessment blinding. Author was contacted, no response received.
Incomplete outcome data (attrition bias) All outcomes	Low risk	Balanced attrition and reasons given
Selective reporting (reporting bias)	Unclear risk	No protocol/trial registration. Appropriate outcomes reported as per the method section
Other bias	Low risk	No baseline imbalances. Clustering taken into account for the analysis. No other concerns.

**Table:** Moreau 2021

***Study characteristics***		
Methods	**Study design:** prospective RCT **Study duration:** NR **Setting:** 19 French Tertiary Centres	
Participants	**State of disease at beginning of study per IG/CG:** NR **Disease type per IG/CG:** mixed N (%) in IG**:** CD 95 (71.4%), UC 38 (28.6%) N (%) in CG: CD 97 (75.2%), UC 32 (24.8%) **Inclusion criteria:** adults aged between 18 and 70 years; diagnosed with IBD (CD or UC); with either a recent diagnosis (less than 6 months), or significant event in the disease course and/or change in treatment (recent hospitalisation, complication, surgery, or immunosuppressant or biologic considered). **Exclusion:** patients unable to communicate, understand, or participate in the educational programme, mainly for linguistic reasons. **Age at beginning of study per IG/CG:** median age (IQR): IG 29.9 (25.2–42.0); CG 32.5 (24.9–42.2) **Sex per IG/CG:** N (%) IG Male 54 (40.6%) Female 79 (59.4%); CG Male 51 (39.5%) Female 78 (60.5%) **Disease duration per IC/CG:** median (IQR) months IG: 49.5 (6.4‐111.9); CG: 40.6 (7.3‐ 122.8) **Number randomised per IG/CG:** IG: 133; CG: 130 **Number reaching end of study per IG/CG:** IG: 133; CG: 129	
Interventions	**IG:** education programme delivered by a dedicated staff (mainly nurses) using an illustrated book, covering the different dimensions of life with IBD. **CG:** no education programme. After 6 months, there was a cross‐over procedure and patients from the control group followed the same programme as the educated group.	
Outcomes	**Duration of follow ‐up:** 12 months **Primary outcomes as defined by study authors:** the psycho‐pedagogic impact of the education programme on IBD patients’ skills with regard to their disease. It was measured by the change in composite ECIPE score from baseline to 6 months. **Secondary outcomes as defined by study authors:** the changes of ECIPE scores between 6 and 12 months in both groups; the impact of the education programme on disease progression (rates of hospitalisation, complications, or surgery), adherence to treatment (assessed by the modified Morisky adherence scale), quality of life (assessed by the short quality of life score for IBD (SIBDQ)), work productivity (assessed by the work productivity and activity impairment questionnaire (WPAI)), and patients’ concerns (assessed by the RFIPC); the impact of the education programme on the health care professionals performing the sessions.	
Notes	**Funding:** financially supported by grants from MSD France and Association François Aupetit. **Conflict of interest:** JM received honoraria from MSD, Janssen, Abbvie, Pfizer, Ferring, Takeda, and Vifor; NH received honoraria from Janssen, Tillots Pharma, and MSD; LM received honoraria from Bayer, Merck, Novartis, Takeda; CTP received lecture fees from Abbvie, Takeda, Maat Pharma, Janssen, and advisory board fees from MSD and Tillots; MN received honoraria from Abbvie, Adacyte, Amgen, Biogen, Ferring, Janssen, Mayoli‐Spindler, MSD, Pfizer, and Takeda; RA received advisory board fees from Takeda, Abbvie, Norgine, Tillots, MSD, Biogen, and Janssen; JCG received honoraria from Abbvie, Pfizer, Janssen, Takeda, and MSD; SO received honoraria from MSD, Abbvie, Janssen,Otsuka, Takeda, Gilead, and GSK; XH received honoraria from Abbvie, Amgen, Biogen, Celltrion, Ferring, HAC Pharma, Hospira, Janssen, MSD, Pfizer, and Takeda; AA received honoraria from Abbvie, Janssen, Takeda, Ferring, and MSD; PS received honoraria from Takeda, MSD, Biocodex, Ferring, Pfizer, and AbbVie, and grant support from Biocodex; XR received honoraria from MSD, Abbvie, Biogen, Pfizer, Janssen, Takeda, and Theradiag; SN received lecturer or advisory board fees from AbbVie, MSD, Vifor Pharma, Pfizer, Janssen, and Ferring; GS received lecture fees and travel grants from MSD, Ferring, Takeda, Pfizer, Janssen, Vifor, HAC Pharma, Abbvie, Tillots, and Norgine; BM has no conflict of interest to declare; CS received honoraria from Takeda, lecture fees from Abbvie, Fresenius Kabi, Pfizer, and Janssen and travel accommodation from MSD, Takeda, Abbvie, Pfizer, and Janssen; MS received honoraria from Abbvie, Takeda, and Mylan: BC received honoraria from Abbvie, Mayoly Spindler, Sanofi, and Kyowa Kyrin; MF received honoraria from Abbvie, Ferring, MSD, Janssen, Takeda, Tillots, Gilead, Celgene, Boehringer, Biogen, Pfizer; FC received honoraria from Amgen, BMS, Celltrion, Enterome, Ferring, Janssen, Medtronic, Pfizer, Pharmacosmos, and Roche, as well as lecture fees from Abbvie, Astra, BMS, Ferring, Janssen, MSD, Pfizer, Pileje, Takeda, and Tillotts; LPB received honoraria from AbbVie, Janssen, Genentech, Ferring, Tillots, Pharmacosmos, Celltrion, Takeda, Boerhinger Ingelheim, Pfizer, Index Pharmaceuticals, Sandoz, Celgene, Biogen, Samsung Bioepis, Alma, Sterna, Nestle, Enterome, Allergan, MSD, Roche, Arena, Gilead, Hikma, Amgen, BMS, Vifor, Norgine, Mylan, Lilly, Fresenius, Oppilan Pharma, Sublimity Therapeutics, Applied Molecular Transport, OSE Immunotherapeutics, Enthera, grants from Abbvie, MSD, Takeda, and has stock options in CTMA; SC has no conflict of interest to declare; MA received honoraria from Abbvie, MSD, Janssen, Takeda, Pfizer, Novartis, Ferring, Tillots, Celgene, and Genentech/Roche.	
***Risk of bias***		
**Bias**	**Authors' judgement**	**Support for judgement**
Random sequence generation (selection bias)	Unclear risk	Randomisation method not specified. Author was contacted, no response received.
Allocation concealment (selection bias)	Unclear risk	No mention of allocation concealment. Author was contacted, no response received.
Blinding of participants and personnel (performance bias) All outcomes	High risk	It is not possible to blind the participants to the intervention (education)
Blinding of outcome assessment (detection bias) All outcomes	Low risk	The ECIPE score was calculated by a physician independent of the education team and blinded to the allocation group of the patient
Incomplete outcome data (attrition bias) All outcomes	Low risk	There was only 1 dropout and reason for dropout mentioned. No baseline imbalance between IG and CG
Selective reporting (reporting bias)	High risk	Trial registration mentions 3 other outcomes (hospitalisation, flare‐ups and major complications), the results of which were not presented in the paper. Also only odd ratios of outcomes were presented. Author was contacted, no response received. Trial number NCT02550158
Other bias	Low risk	No baseline imbalance between IG and CG. No other concerns.

**Table:** Nikolaus 2017

***Study characteristics***		
Methods	**Study design:** prospective RCT **Study duration:** NR **Setting:** multicentre (tertiary referral centre; specialised community hospital; specialised private practice)	
Participants	**State of disease at beginning of study per IG/CG:Intention to treat group** Clinical activity assessed by the Colitis Activity Index (CAI) N (%): 0–4 (remission): CG: 89 (73%); IG: 82 (65.1%) > 4–9 (mild to moderate activity): CG 14 (11.5%); IG 25 (19.9%) > 9 (severe activity): CG 5 (4%); IG 3 (2.4%) Missing: CG: 14 (11.5%); IG: 16 (12.7%) **Disease type per IG/CG:** all participants had UC **Inclusion criteria:** age ≥ 18 years, with diagnosis of UC confirmed by colonoscopy and histology, and a minimum duration of disease of 2 years. Individual exceptions by the lead principal investigator could be given to included patients ≥ 16 years. At inclusion, disease activity had to reflect remission or mild disease CAI ≤ 9), and the participant had to be on a treatment with oral (not rectal) mesalamine (irrespectively of any other treatment) or had to be willing to start a treatment with oral mesalamine with a dose of 1.2 g to 4.8 g/day upon inclusion (medication was provided to all participants by the insurance system, without patients having to pay for it). All participants had to give written informed consent and be willing and able to follow a standardised education programme. **Exclusion:** people with CD or indeterminate colitis, significant comorbidities, a CAI > 9 or an intolerance/contraindication to mesalamine; people after colectomy or with a current ostomy. **Age at beginning of study per IG/CG: intention to treat (ITT) group:** median (range) IG: 46.68 (19.61–88.09); CG: 44.6 (18.41–81.02) **Sex per IG/CG: intention to treat group:** IG: Male 68 (54.4%) Female 58 (45.6%); CG: Male 66 (54.6%) Female 60 (45.4%) **Disease duration per IC/CG:** median (range) years IG: 5.34 (0.35–40.36); CG: 5.71 (0.27–26.64) **Number randomised per IG/CG:** IG: 126; CG: 122 **Number reaching end of study per IG/CG:** IG: 47; CG: 52	
Interventions	**IG:** a standardised education programme delivered by either a certified nurse or the trial physician using a standardised slide set, followed by a group session in which all participants asked questions and a contact for further individual questions (e.g. by telephone or email) was established. **CG:** participants received standard care and were also offered participation in the education programme after the study ended.	
Outcomes	**Duration of follow ‐up:** 14 months **Primary outcomes as defined by study authors:** adherence to mesalamine treatment measured using the Morisky Medication Adherence Scale (MMAS) **Secondary outcomes as defined by study authors:** secondary endpoints included short‐term adherence, quality of life, disease activity, and self‐assessment of adherence. Self‐assessment of adherence was measured by the MMAS21 as described above. To evaluate short‐term adherence, adherence data at the end of the supervision phase were used. Data of the MMAS‐scale were correlated with results of the 5‐aminosalicylic acid measurements in the urine and corrected, if applicable.	
Notes	**Funding source:** supported by a non‐conditional grant of Shire Germany given to the German Competence Network. **Conflicts of interest:** NS, SS, SB, BB, BE, BO, GD, HU, SM, and KW have no relevant conflicts of interest concerning the instant publication.	
***Risk of bias***		
**Bias**	**Authors' judgement**	**Support for judgement**
Random sequence generation (selection bias)	Unclear risk	Method of randomisation not specified. Author was contacted, no response received.
Allocation concealment (selection bias)	Unclear risk	No mention of allocation concealment. Author was contacted, no response received.
Blinding of participants and personnel (performance bias) All outcomes	High risk	It is not possible to blind the participants to the intervention (education)
Blinding of outcome assessment (detection bias) All outcomes	High risk	Author states that it was a "non‐blinded trial"
Incomplete outcome data (attrition bias) All outcomes	Unclear risk	Large number of withdrawals but balanced and reasons given for all. No imbalance between groups. However, it's not unclear how many people were randomised as the text mentions 248 but the flow‐diagram 258. It's not explained neither in the text nor the flow diagram what happened to the remaining 10 participants, only that one patient withdrew consent before randomisation. Author was contacted, no response received.
Selective reporting (reporting bias)	Unclear risk	Results of all outcomes mentioned in trial registration were included in the methods.However, a statement on several secondary outcomes was made with no specific data. The authors were contacted but no response received. Trial number: DRKS00008905
Other bias	Low risk	No baseline imbalance between IG and CG. No other concerns.

**Table:** Oxelmark 2007

***Study characteristics***		
Methods	**Study design:** prospective RCT **Study duration:** The first intervention group (UC) was started in 1996, the second (CD) in 1997, the third (UC) in 1999, and the fourth (CD) in 2000. **Setting:** IBD‐outpatient clinic at the Karolinska University Hospital Huddinge, Stockholm, Sweden	
Participants	**State of disease at beginning of study per IG/CG:** all patients were in remission or had low disease activity at inclusion, but numbers were not specified. **Disease type per IG/CG:** mix of UC and CD. **UC:** IG: 11; CG: 6 **CD:** IG: 13; CG: 14 **Inclusion criteria:** people with UC or CD who had had at least one serious flare and had been treated at least once with glucocorticosteroids (GCS) orally or intravenously. All patients had to be in remission or have only mild disease activity at inclusion. **Exclusion:** patients with high‐dose steroid treatment (more than 10 mg prednisolone or equivalent), blood in stools, and previous bowel surgery. **Age at beginning of study per IG/CG:** mean (range) years: IG: 36.3 (18‐71); CG: 38.5 (21‐59) **Sex per IG/CG:** Male: IG: 11; CG: 7; Female: IG: 13; CG: 13 **Disease duration per IC/CG:** mean (range) years IG: 4.6 (1‐11); CG: 5.2 (1‐10) **Number randomised per IG/CG:** IG: 24; CG: 22 **Number reaching end of study per IG/CG: At 6 months:** IG 18; CG 15. **At 12 months:** IG 20; CG 15	
Interventions	**IG:** nine different sessions (once a week, each session lasted for 1½ hours) for about 3 months, with lectures alternating with group therapy. **CG:** patients in the control groups received conventional “on demand” medical and psychosocial/psychological treatment during the study period.	
Outcomes	**Duration of follow‐up:** 12 months **Primary outcomes as defined by study authors:** HRQoL measured using the IBDQ and coping ability measured using the Sense of Coherence scale (SOC) **Secondary outcomes as defined by study authors:** patient evaluation of the intervention using a visual analogue scale (VAS)	
Notes	**Funding source:** NR **Conflicts of interest:** NR	
***Risk of bias***		
**Bias**	**Authors' judgement**	**Support for judgement**
Random sequence generation (selection bias)	Low risk	Author response: "For each time point patients’ participation in either control or treatment group was decided by a simple lottery. Ten patients who had accepted to participate (for each time point) and were carefully informed that they would be allocated to either intervention or control group. The patient’s names were written separately on small paper notes. Each note was folded separately and placed into a hat, and one of the researchers then took out one note at a time without looking into the hat. Every second note was allocated to the control group and the others to the intervention group"
Allocation concealment (selection bias)	High risk	Patients were "pre‐allocated", as explained above by the author, and there was no concealment of the allocation list.
Blinding of participants and personnel (performance bias) All outcomes	High risk	It is not possible to blind the participants to the intervention.
Blinding of outcome assessment (detection bias) All outcomes	High risk	Author response: assessors were not blinded.
Incomplete outcome data (attrition bias) All outcomes	Low risk	Reasons for dropouts were given and no imbalance between IG and CG
Selective reporting (reporting bias)	Unclear risk	No protocol or trial registration. Outcomes reported as per the methods section.
Other bias	Low risk	No baseline imbalance between IG and CG. No other concerns.

**Table:** Uran 2019

***Study characteristics***		
Methods	**Study design:** prospective RCT **Study duration:** 8 weeks **Setting:** gastroenterology polyclinic	
Participants	**State of disease at beginning of study per IG/CG:** At baseline: N (%) disease activity of UC: Remission IG (web‐based education): 5 (31.3%); CG (standard education): 4 (25.0%) N (%) disease activity of CD: Remission IG (web‐based education): 5 (35.6%); CG (standard education): 9 (64.3%) **Disease type per IG/CG:** IG (web‐based education): 16 UC and 14 CD CG (standard education): 16 UC and 14 CD **Inclusion criteria:** people were diagnosed with IBD at least six months previously, able to use computer, Internet and mobile phone and aged 18 years and over. **Exclusion:** people with advanced comorbid diseases such as cancer, diabetes, chronic obstructive pulmonary disease, hypertension **Age at beginning of study per IG/CG:** Mean (unspecified type of variation): IG (web‐based education): 37.26 (± 12.99) CG (standard education): 41.63 (± 11.85) **Sex per IG/CG:** Numbers in IG (web‐based education): 13/30 females and 17/30 males Numbers in CG (standard education): 12/30 females and 18/30 males **Disease duration per IC/CG:** mean (SD) months IG: 82.23 (54.52); CG: 81.93 (56.71) **Number randomised per IG/CG:** IG (web‐based education): 30; CG (standard education): 30 **Number reaching end of study per IG/CG** IG (web‐based education): 30; CG (standard education): 30	
Interventions	**IG(web‐based education):** information presented via online web‐site **CG (standard education):** information presented via easy‐to‐read, illustrated, colour‐printed books Both group had exactly the same educational content that differed only in mode of delivery	
Outcomes	**Duration of follow‐up:** NR **Primary outcomes as defined by study authors:** the effect of web‐based education on disease activity, symptom management and quality of life **Secondary outcomes as defined by study authors:** NR	
Notes	**Funding source:** the authors declared no financial supports **Conflicts of interest:** the authors declared no conflict of interest	
***Risk of bias***		
**Bias**	**Authors' judgement**	**Support for judgement**
Random sequence generation (selection bias)	Unclear risk	Simple randomisation and stratified randomisation were made separately according to the criteria of age, gender, educational level and duration of disease. Randomisation was made by a statistician. Author was contacted, no response received.
Allocation concealment (selection bias)	Unclear risk	No details. Author was contacted, no response received.
Blinding of participants and personnel (performance bias) All outcomes	High risk	The researcher was blinded to the randomisation process but participants knew which treatment they received
Blinding of outcome assessment (detection bias) All outcomes	Unclear risk	No mention of any outcome assessment blinding. Author was contacted, no response received.
Incomplete outcome data (attrition bias) All outcomes	Unclear risk	No patient flow details given. Author was contacted, no response received.
Selective reporting (reporting bias)	Unclear risk	No protocol/trial registration. Appropriate outcomes reported as per the method section
Other bias	Low risk	No baseline imbalance. No other concerns.

**Table:** Vaz 2019

***Study characteristics***		
Methods	**Study design:** prospective RCT **Study duration:** NR **Setting:** gastroenterology clinic	
Participants	**State of disease at beginning of study per IG/CG:** all randomised participants had inactive disease at baseline. **Disease type per IG/CG:** mixed 67% had CD and 33% had UC, numbers were not specified for IG and CG **Inclusion criteria:** confirmed IBD diagnosis (i.e. CD, UC, or indeterminate colitis), prescribed at least 1 daily oral medication for the control of IBD (i.e. steroid, thiopurine, or aminosalicylate). **Exclusion:** significant developmental disorders or serious mental illness, enrolment in another intervention targeting adherence at the time of the study **Age at beginning of study per IG/CG:** mean age (SD) of randomised participants in the study was 14.9 years (1.9); mean ages were not specified for the IG and CG separately. **Sex per IG/CG:** 44% of all randomised participants were girls, and 56% were boys. Specific numbers for IG and CG not provided. **Disease duration per IC/CG:** NR **Number randomised per IG/CG:** IG: 7, CG: 6 **Number reaching end of study per IG/CG:** IG: 5, CG: 4	
Interventions	**IG:** a 30‐minute educational session using the IBD Pocket Guide **CG:** participants received usual care	
Outcomes	**Duration of follow‐up**: 4 weeks **Primary outcomes as defined by study authors:** medication adherence measured via the MedMinder Pill Dispensing System. **Secondary outcomes as defined by study authors:** IBD knowledge measured using the IBD Knowledge Inventory Device (IBD‐KID). Transition readiness measured using the c(TRAQ) Version 5.0	
Notes	**Funding source:** this study was supported by a National Institutes of Health training grant awarded to the Cincinnati Children’s Hospital Medical Center Division of Pediatric Gastroenterology, Hepatology and Nutrition (T32 DK007727). **Conflicts of interest:** none	
***Risk of bias***		
**Bias**	**Authors' judgement**	**Support for judgement**
Random sequence generation (selection bias)	Low risk	Computer‐generated block randomisation (information provided by the author)
Allocation concealment (selection bias)	High risk	Only the lead investigator had access to the allocation and followed the randomisation schedule after they finished the run‐in period and qualified for the study (information provided by the author)
Blinding of participants and personnel (performance bias) All outcomes	High risk	It is not possible to blind the participants to the intervention.
Blinding of outcome assessment (detection bias) All outcomes	High risk	Assessors were not blinded (information provided by the author)
Incomplete outcome data (attrition bias) All outcomes	Low risk	Equal attrition between groups and the authors confirmed that in all cases, the participants found the study too difficult to complete and withdrew
Selective reporting (reporting bias)	Unclear risk	No trial registration or protocol. Results for all outcomes mentioned in the method section were reported, but not all raw data is clearly reported per group. Authors could not provide it.
Other bias	Unclear risk	Baseline characteristics of IG vs CG not reported. Authors could not provide them. No other concerns.

**Table:** Walkiewicz 2011

***Study characteristics***		
Methods	**Study design:** prospective RCT **Study duration:** NR **Setting:** IBD clinic	
Participants	**State of disease at beginning of study per IG/CG:** NR **Disease type per IG/CG:** IBD (UC and CD) Specific figures NR in abstract **Inclusion criteria:** NR **Exclusion:** NR **Age at beginning of study per IG/CG:** age range 11‐21 years **Sex per IG/CG:** NR **Disease duration per IC/CG:** NR **Number randomised per IG/CG:** total randomised 36 **Number reaching end of study per IG/CG:** NR	
Interventions	**IG:** IG1: "Internet blog access" IG2: "the receipt of text messaging" IG3: "combination of Internet blog access and text messaging" **CG:** standard care	
Outcomes	**Duration of follow‐up:** 3 months **Primary outcomes as defined by study authors:** disease‐related knowledge assessed using a modified version of the Crohn's and Colitis Foundation of America (CCFA) Knowledge Score (I‐M‐AWARE) **Secondary outcomes as defined by study authors:** NR	
Notes	**Funding source:** NR **Conflicts of interest:** NR	
***Risk of bias***		
**Bias**	**Authors' judgement**	**Support for judgement**
Random sequence generation (selection bias)	Unclear risk	Only the abstract was available as contact person for the study said he no longer has access to the study.
Allocation concealment (selection bias)	Unclear risk	No information (abstract only and author responded they have no access to the study data anymore)
Blinding of participants and personnel (performance bias) All outcomes	High risk	It is not possible to blind the participants to the intervention
Blinding of outcome assessment (detection bias) All outcomes	Unclear risk	No information (abstract only and author responded they have no access to the study data anymore)
Incomplete outcome data (attrition bias) All outcomes	Unclear risk	No information (abstract only and author responded they have no access to the study data anymore)
Selective reporting (reporting bias)	Unclear risk	Results presented in the abstract are reflective of the outcomes mentioned in the method section but the data is not clearly presented per group
Other bias	Unclear risk	No information on baseline characteristics (abstract only and author responded they have no access to the study data anymore). No other concerns.

**Table:** Waters 2005

***Study characteristics***		
Methods	**Study design:** prospective RCT **Study duration:** NR **Setting:** outpatient clinic	
Participants	**State of disease at beginning of study per IG/CG:** mean (nature of figures provided in brackets unclear in source article) CDAI mean score: IG: 126.8 (93.3); CG: 188.3 (117.1) (low score = better result) Seo UC activity index (mean score) IG:111.8 (±25.8), CG: 114.1 (±37.8) (low score = better result) **Disease type per IG/CG:** mixed: UC/CD IG: 14/31; CG: 18/26 **Inclusion criteria:** people 17 years of age and older with a diagnosis of IBD confirmed by radiographic/endoscopic examination and/or histology/surgical pathology, who lived within a 2‐hour drive of the University of Alberta Hospital (Edmonton, Alberta), were able to attend the education programme, and had fluency in written and spoken English. **Exclusion:** people with short gut syndrome, disease limited to ulcerative proctitis, a proctocolectomy for UC, an ostomy, on total parenteral nutrition, or who underwent surgery during the study that required an ostomy **Age at beginning of study per IG/CG:** mean (SD) IG: 40.3 (12.8); CG: 45.0 (13.5) **Sex per IG/CG:** male/female IG: 29/16 CG: 22/22 **Disease duration per IC/CG:** mean (SD) years IG: 10.5 (9.0); CG: 13.4 (9.84) **Number randomised per IG/CG:** IG: 45; CG: 44 **Number reaching end of study per IG/CG:** IG: 31; CG: 38	
Interventions	**IG:** the education group, in addition to standard of care, attended a 12‐hour structured education programme provided in 3‐ hour blocks over four consecutive weeks. **CG:** received standard care consisting of physician visits at the discretion of the physicians and patients, with physician‐directed ad hoc teaching during visits and the presentation of printed educational literature. The control group was offered the full education programme after the study data collection was completed.	
Outcomes	**Duration of follow‐up:** 8 weeks **Primary outcomes as defined by study authors:** knowledge, assessed using the Crohn’s and Colitis Knowledge Questionnaire (CCKNOW) and the Knowledge Questionnaire (KQ) and QOL measured using IBDQ and RFIPC **Secondary outcomes as defined by study authors:** medication adherence was assessed by three methods: survey at baseline; a set of questions on the Patient Satisfaction Questionnaire; and participant self‐report; healthcare use measured by number of physician visits and hospital admissions related to IBD and the associated complications; participant satisfaction with medical care and the education programme was assessed with questionnaires using a Likert scale with 1 being “strongly agree” and 4 being “strongly disagree”.	
Notes	**Funding source:** NR **Conflicts of interest:** NR	
***Risk of bias***		
**Bias**	**Authors' judgement**	**Support for judgement**
Random sequence generation (selection bias)	Unclear risk	Randomisation method not specified. Author was contacted, no response received.
Allocation concealment (selection bias)	Unclear risk	No mention of allocation concealment. Author was contacted, no response received.
Blinding of participants and personnel (performance bias) All outcomes	High risk	It is not possible to blind the participants to the intervention.
Blinding of outcome assessment (detection bias) All outcomes	Unclear risk	No mention of blinding of outcome assessor. Author was contacted, no response received.
Incomplete outcome data (attrition bias) All outcomes	Low risk	The IG lost 14/45 and the CG lost 6/44. Reasons for drop‐outs given and we don't think they have an impact on the outcomes.
Selective reporting (reporting bias)	Unclear risk	The authors do not provide clear raw data for multiple results (QOL, medication adherence, healthcare visits) in their results section. Author was contacted, no response received.
Other bias	Low risk	No baseline imbalance between IG and CG. No other concerns.

**Table:** Weizman 2021

***Study characteristics***		
Methods	**Study design:** cluster‐RCT **Study duration:** January 2017 to January 2018 **Setting:** tertiary IBD centres	
Participants	**State of disease at beginning of study per IG/CG:** all participants had active disease flare‐ups **Disease type per IG/CG:** 91 patients with UC **Inclusion criteria:** people with a known diagnosis of UC admitted to hospital for an acute disease flare between January 2017 and January 2018. Only the first UC flare hospitalisation during the study period was included for each participant. **Exclusion:** people with CD and those unable to provide informed consent **Age at beginning of study per IG/CG:** the figures reporting age were labelled 'age at admission' and did not state the nature of the numbers provided; they are probably mean (spread) values, but presented in the source material as "n(%)": IG: 32.1 (11.4%); CG: 35.6 (12.6%) **Sex per IG/CG:** IG: 21/46 males and 25/46 females CG: 22/45 males and 23/45 females **Disease duration per IC/CG:** NR **Number randomised per IG/CG:** CG: 45; IG: 46 **Number reaching end of study per IG/CG:** CG: 42; IG: 45	
Interventions	**IG (educational intervention):** participants were provided with an iPad containing specific patient‐directed educational material which focused on the optimal in‐hospital management of acute severe UC. The educational intervention was an original, interactive video that provided a summary of the 2012 Canadian consensus statements on the treatment of hospitalised adult patients with severe UC, and it used a patient‐friendly languages and images. **CG:** standard care	
Outcomes	**Duration of follow‐up**: 6 months Total duration of the study was 12 months **Primary outcomes as defined by study authors:** patient‐reported outcomes: Trust in Physician Scale (SD), Global CACHE score using questionnaire, hospital anxiety and depression score (HADS), and clinical outcomes, which were overall length of stay, the development of hospital‐acquired venous thromboembolism and the occurrence of colectomy. **Secondary outcomes as defined by study authors:** NR	
Notes	**Funding source:** funded in part through an unrestricted educational grant from Abbvie **Conflicts of interest:** A.V.W. has served as an advisory board member for Abbvie, Janssen, Takeda, Ferring and as a speaker for Abbvie, Janssen, Takeda, Ferring, Pfizer; B.B. has served as an advisory board member and speaker for Ferring, Janssen, Abbvie, Takeda, Pfizer, Novartis, Merck, an advisory board member for Robarts Clinical Trials, Celgene, Microbiome Insights, Merck, Amgen, Pendopharm, Genentech, BMS, Allergan, Protagonist and has received research support from Janssen, Abbvie, GSK, BMS, Amgen, Genentech, Merck, BI, Qu Biologic, Celgene, Alvine. He owns stock options in Qu Biologic; C.H.S. has served as an advisory board member for Janssen, Abbvie, Takeda, Ferring, Shire, Pfizer and as a speaker for Janssen, Abbvie, Takeda, Ferring, Shire, Pfizer; W.A. has served as an advisory board member for Janssen, Abbvie, Takeda, Merck, Pfizer and has received research support from Theradiag, Prometheus; N.M.A.: none; L.T. has served as an advisory board member for Janssen, Abbvie, Merck, Pfizer, Takeda, Mallinckrodt, as a speaker for Janssen, Takeda and has received research funding from Janssen; D.H.N.: none; J.L.J. has served as an advisory board member for Janssen, Merck, Pfizer, Abbvie, Shire, Takeda and as a speaker for Janssen, Pfizer, Abbvie, Shire, Takeda; V.H.: none; S.K.M. has served as an advisory board member for Takeda, Ferring, Shire, Abbvie and as a speaker for Ferring, Pfizer; G.C.N: none	
***Risk of bias***		
**Bias**	**Authors' judgement**	**Support for judgement**
Random sequence generation (selection bias)	Unclear risk	Computer‐generated randomisation was performed centrally. Patients admitted with a UC flare within a specific 6‐month time period were allocated to the intervention designated for that time cluster. At the end of the 6‐month time period, sites that had been randomised to the intervention group would then return to usual care or vice versa (Information provided by the author). Although the clusters were randomised at the same time, the researchers had to decide whether individual patients should be entered into the study which raises the possibility of bias: "All hospital admissions were scanned daily on weekdays by research staff to identify potential enrollees, and suitable participants were approached to participate in the study"
Allocation concealment (selection bias)	Low risk	As this is a cluster‐RCT lack of concealment of an allocation sequence should not be an issue
Blinding of participants and personnel (performance bias) All outcomes	High risk	Not possible to blind study participants and personnel for this study
Blinding of outcome assessment (detection bias) All outcomes	High risk	The outcome assessors were not blinded.
Incomplete outcome data (attrition bias) All outcomes	Low risk	Small number of patients withdrawing with reasons given and no imbalance between IG and CG
Selective reporting (reporting bias)	Low risk	Study has a trial registration NCT02569333) which and the stated outcomes are presented in the results
Other bias	Low risk	No baseline imbalances and clustering taken into account for the analysis. No other concerns.

**CCFA:** Crohn's & Colitis Foundation of America; **CD:** Crohn's disease; **CDAI:** Crohn's Disease Activity Index; **CG:** control group; **GI:** gastrointestinal; **HBI:** Harvey‐Bradshaw Index; **HRQoL:** health‐related quality of life; **IBD:** inflammatory bowel disease; **IBDQ:** Inflammatory Bowel Disease Questionnaire; **IG:** intervention group; **ITT:** intention to‐treat; **IQR:** interquartile range; **IV:** intravenous; **NR:** not reported; **QOL:** quality of life; **QuICC:** Quality Index in Crohn’s and Colitis; **RCT:** randomised controlled trial; **RFIPC:** Rating Form of IBD Patient Concerns; **SC:** subcutaneous; **SD:** standard deviation; **SCCAI:** Simple Clinical Colitis Activity Index; **TNF:** tumour necrosis factor; **UC:** ulcerative colitis; **UCEIS:** Ulcerative Colitis Endoscopic Index of Severity

### Characteristics of excluded studies [ordered by study ID]

**Table:**

Study	Reason for exclusion
Bregenzer 2005	Not an RCT
Chapman 2020	Not an RCT
Cheema 2020	Not an RCT
Cross 2012	Ineligible intervention ‐ disease‐specific education was given to both groups
Dewulf 2011	Ineligible intervention
Eaden 2002	Ineligible intervention – on cancer
Elkjaer 2010a	Ineligible intervention ‐ minimal education and primarily telehealth tool
Elkjaer 2010b	Not an RCT
Gerbarg 2015	Ineligible study design ‐ not an RCT ("After signing informed consent, subjects were randomised to the 2 groups according to numbers assigned to each subject as they arrived at the first session (baseline visit). Each subject was designated a number (001, 002, 003, etc.) in the order of his or her arrival.")
Greenley 2015	Ineligible intervention ‐ education was on a form of psychological therapy and not IBD (problem solving)
Hueppe 2020	Ineligible intervention
IRCT2015041921850N1	Ineligible intervention (no focus on or adequate educational element)
Kamat 2018	Not an RCT
Karia 2012	Ineligible intervention
Korzenik 2016	Not an RCT
Kyaw 2014	Ineligible intervention
Lange 1996	Not an RCT
Ley 2020	Ineligible intervention
Lim 2020	It was not RCT
Long 2020	Ineligible intervention
Maya 2012	Ineligible intervention
Meng 2018	Ineligible intervention ‐ not IBD focused
NCT00248742	Ineligible intervention
NCT03059186	Ineligible intervention
NCT03186872	Ineligible intervention
NCT04207008	Ineligible intervention
Norton 2015	Ineligible population (faecal incontinence)
Reusch 2016	Ineligible intervention (pschoeducational program)
Robinson 2001	Ineligible intervention – self‐management
Schimdt 2018	Not an RCT
Siegel 2018	Ineligible intervention
Sutton 2019	Ineligible intervention (not IBD education)
Tsavdaroglou 2019	Ineligible intervention ‐ not IBD education
Tung 2015	Not an RCT
Wang 2020	Not an RCT
Wierstra 2018	Ineligible intervention (focus on reproductive knowledge)
Zhang 2020	Ineligible population

**IBD:** inflammatory bowel disease; **RCT:** randomised controlled trial

### Characteristics of studies awaiting classification [ordered by study ID]

**Table:** Almario 2022

Methods	RCT
Participants	152 patients
Interventions	CG: biologics fact sheet IG: IBD&me ‐ a freely available, unbranded, interactive decision aid
Outcomes	The primary outcome was patient perception of SDM as measured by the 9‐Item SDM Questionnaire
Notes	This study was identified during the update search for this review and will be included in the analysis when this review is updated

**Table:** Atreja 2015

Methods	RCT
Participants	IG:162 CG: 158
Interventions	IG: HealthPROMISE application CG: patient education app
Outcomes	Change in quality of care Disparities in IBD‐related emergency room visits and hospitalisations Change in QOL score from baseline Proportion of patients reporting controlled disease status
Notes	We contacted the authors on 15 June 2021 about information about the patient education element of their trial but received no response

**Table:** De Dycker 2022

Methods	RCT
Participants	447 IBD patients
Interventions	In centre A, all patients were informed on the new SC formulations and the accompanying care pathway by an information leaflet and a face‐to‐face interaction with the IBD nurse, prior to completing the survey In centre B, patients on a minimal interval of every 8 weeks were digital invited to the same survey via the e‐health application of the hospital
Outcomes	Demographics, patient reported outcomes, willingness to switch and reasons for IV vs. SC preferences were captured
Notes	This study was identified during the update search for this review and will be included in the analysis when this review is updated

**Table:** DRKS00022935

Methods	RCT
Participants	100
Interventions	IG: education programme CG: standard therapy
Outcomes	Primary: change in medical knowledge on IBD Secondary: none
Notes	We contacted authors for a full report on 15 June 2021 and we received no response

**Table:** Haslbeck 1996

Methods	Quasi‐experimental prospective trial
Participants	IG: 18 CG: 18
Interventions	IG: interdisciplinary training programme for IBD patients CG: no training
Outcomes	Disease activity Medication use Disease knowledge Self‐activity Depression QOL Beliefs of internal control Social activity
Notes	We contacted authors to determine whether this was an RCT on 15 June 2021 and we received no response

**Table:** Homel 2015

Methods	RCT
Participants	194
Interventions	IG: telehealth behavioural treatment CG: education only
Outcomes	Medication adherence Disease severity Patient QOL Health care utilisation
Notes	We contacted authors for a report of their results on 15 June 2021 and we received no response

**Table:** IRCT20180520039736N1

Methods	RCT
Participants	45
Interventions	Group 1:education of Lifestyle Group 2: the education of mindfulness based cognitive therapy Group 3: on the waiting list: an individual counselling session during interventions
Outcomes	Severity of fatigue Disease activity Self‐care agency Anxiety Depression QOL Illness perception Perceived stress Disease‐related worries and concerns
Notes	We contacted authors for a report of their results on 15 June 2021 and we received no response

**Table:** IRCT20191026045251N1

Methods	RCT
Participants	90
Interventions	IG: Teach back group (content will be taught to patients in 2 sessions (2 days) for 45 minutes. The training package will finally be provided to the patient within 7 days. The interval between training sessions will be 1‐3 days.) CG: App group: the app will be installed in a 30‐minute session for patients, and how to use the app will be explained to them.
Outcomes	QOL Lifestyle Illness severity Recurrence and remission of the disease
Notes	We contacted authors for a report of their results on 15 June 2021 and we received no response

**Table:** ISRCTN67674151

Methods	RCT
Participants	106
Interventions	IG: a nurse‐led consultation lasting a minimum of 30 minutes. Patient's beliefs and attitudes to medication adherence are discussed, strategies developed to increase adherence and information and support regarding management of their UC will be offered. The intervention will be delivered using a concordance‐led consultation. CG: no intervention.
Outcomes	Medication adherence Changes in IBD‐specific quality of life Disease activity and relapse Additional explanatory variables are: illness perception (IPQ‐R), beliefs about medicines (BMQ), self reported medication adherence, preferred role in the decision making process (Degner and Beatton patterns of decision‐making framework)
Notes	We contacted authors for a report of their results on 15 June 2021 and we received no response

**Table:** Lorenzon 2016

Methods	RCT
Participants	IG: 81 CG: 81
Interventions	IG: patient support programme CG: conventional management
Outcomes	Disease activity Adherence to therapy Work productivity and daily activities QOL
Notes	We contacted authors about the educational element of their trial on 15 June 2021 and we received no response

**Table:** Magharei 2019

Methods	RCT
Participants	IG:42 CG: 42
Interventions	IG: self‐management education CG: routine training
Outcomes	Self‐efficacy QOL
Notes	We contacted authors about the educational element (not clear if the topic study is education or CBT) of their trial on 15 June 2021 and we received no response

**Table:** Martinato 2022

Methods	RCT
Participants	49 IBD patients
Interventions	CG: “standard of care” information package IG: synthetic, standardised, and structured informative intervention developed with a “less is more” approach in collaboration with local IBD patients’ associations
Outcomes	Knowledge level was measured with the Crohn’s and Colitis Knowledge score – CCKNOW Perception of QOL with the Short Inflammatory Bowel Disease Quality of life ‐ SIBDQ Disease activity was assessed with the Modified Trulove and Witt Severity Index ‐ MTWSI ‐ in ulcerative colitis and the Harvey Bradshaw Index – HBI – in Crohn’s disease
Notes	This study was identified during the update search for this review and will be included in the analysis when this review is updated

**Table:** Menze 2022

Methods	RCT
Participants	30 IBD patients, aged 10‐18 years
Interventions	IG: app for Android software called KARLOTTA (Kids + Adolescents Research Learning On Tablet Teaching Aachen) CG: unclear
Outcomes	Outcome parameters were an increase in knowledge, changes in quality of life and analysis of the feedback questionnaires for patient and physician
Notes	This study was identified during the update search for this review and will be included in the analysis when this review is updated

**Table:** Moshkovska 2010

Methods	RCT
Participants	71
Interventions	IG: education and motivation session; participants also received an educational leaflet. Relatives and friends were also invited to attend and a leaflet specifically designed for these people was also offered. Optional components of the tailored intervention included simplification of the dosing regime if clinically appropriate and a choice of practical reminders such as pill dispensers with and without alarms. CG: no details
Outcomes	Medication adherence Beliefs about medication Satisfaction with information
Notes	We contacted authors about the educational element of their trial on 15 June 2021 and we received no response

**Table:** NCT03695783

Methods	RCT
Participants	152
Interventions	IG: online decision aid called IBD&me CG: standardised educational material
Outcomes	Patient perceptions of shared decision‐making (primary) Patient perceptions of decisional conflict Patient satisfaction Disease control and IBD‐related QO Initiation or switch of a treatment
Notes	We contacted authors about a full report of their results on 15 June 2021 and we received no response

**Table:** NCT04183608

Methods	RCT
Participants	238
Interventions	Group1: adalimumab + patient education + calprotectin + e‐monitoring Group 2: adalimumab Group 3: e‐Monitoring, home fecal calprotectin testing and therapy education Group 4: standard of care (patient only visits every 3 months the doctor so the optimisation of treatment can be done only at this frequency)
Outcomes	Endoscopic remission (primary) Clinical remission (defined as a total Mayo score ≤ 2 points, with no individual sub score > 1, and a Mayo endoscopy sub score of 0 or 1) Remission without steroids Endoscopic healing rate with Mayo score 0 or 1 UCEIS score Histological healing (Nancy score) Remission rate and remission rate without steroids at study visits and W48 Quality of life evolution (evaluate visit W0 vs W14, W26, W38 and W48) Patient satisfaction Continuous response Safety and tolerability Anti‐TNF pharmacokinetics Number of visits in trial Number of UC‐related hospitalisations Number of colectomies Treatment compliance (questionnaire) Patient adhesion (questionnaire) Medico‐economic analysis
Notes	We contacted authors about a full report of their results on 15 June 2021 and we received no response

**Table:** Otilia 2019

Methods	RCT
Participants	IG: 30 CG: 30
Interventions	IG: specialised educational and psychological counselling (SEPC) CG: current medical practices
Outcomes	Disease activity QOL Personality traits
Notes	We contacted authors about the educational element (is it education or CBT) of their trial on 15 June 2021 and we received no response

**Table:** Stewart 2009

Methods	RCT
Participants	Unknown
Interventions	Unknown
Outcomes	Unknown
Notes	We contacted authors about information on their trial on 15 June 2021 and we received no response. We could not find the content of this abstract/poster in any other way.

**Table:** Ying  2020

Methods	RCT
Participants	IG: 32 CG: 32
Interventions	IG: micro‐lecture and workshop education CG: routine verbal and demonstration education
Outcomes	Differences in the rate of blockage Accidental extubation Aspiration rate of enteral nutrition catheters Patients’ satisfaction with nursing work
Notes	We contacted authors about the full report of their trial on 15 June 2021 and we received no response. We could not find the content of this paper in any other way.

**Table:** Zhuo 2021

Methods	RCT
Participants	63 UC patients
Interventions	IG: health education method based on WeChat platform and oriented by the trans theoretical model and stages of change (TTM) on the positive emotion, negative emotion and self‐care ability CG: unclear
Outcomes	Positive emotion and negative emotion, self‐care ability, quality of life, bloody stool and recurrence within 24 weeks
Notes	This study was identified during the update search for this review and will be included in the analysis when this review is updated

**BMQ:** Beliefs about Medicines Questionnaire**; CG:** control group; **IBD:** inflammatory bowel disease; **IG:** Intervention group; **IPQ‐R:** Illness‐Perception Questionnaire Revised; **IV:** intravenous; **QOL:** Quality of Life; **RCT:** randomised controlled trial; **SC:** subcutaneous; **SDM:** Shared Decision Making Questionnaire; **TNF:** tumour necrosis factor; **UC:** ulcerative colitis; **UCEIS:** Ulcerative Colitis Endoscopic Index of Severity; **W:** week

### Characteristics of ongoing studies [ordered by study ID]

**Table:** IRCT201510137612N2

Study name	A clinical trial to study the effect of information prescription in reducing relapse among patients with inflammatory bowel disease
Methods	RCT
Participants	160
Interventions	The description of the interventions was not entirely clear. IG: the intervention appears to be in the form of a written copy of the information prescribed by a doctor in addition to the usual information that is given to patients. The description continues, "Treatment with oral prescription information to librarians about the information they receive. Information content includes information about inflammatory bowel disease, medications, tests, symptoms, exercise and self‐care is health." CG: "The control group did not receive written information and oral librarian. Patients in control group only received verbal information typically provided by your doctor, and the doctor does not receive health information."
Outcomes	Reduction of relapses (primary) Medical expenses "Frequency of complications Hypochondrias" Change in the number of drugs and dose of intake QOL
Starting date	23 July 2015
Contact information	Dr Vahideh Zarea: vgavgani@gmail.com
Notes	The authors responded to us that the RCT has been completed but they have not published the results yet.

**Table:** IRCT20170731035424N2

Study name	The effect of an educational‐supportive program based on chronic care model on self‐efficacy and health related quality of life of patients with ulcerative colitis
Methods	RCT
Participants	Target sample size: 70
Interventions	IG: programme containing 4 components of the chronic care model CG: regular visits to the doctor as usual
Outcomes	Self‐efficacy score based on Strategies Used by People to Promote Health Questionnaire Health‐related quality of life score based on Inflammatory Bowel Disease Questionnaire(IBDQ‐9)
Starting date	2 August 2021
Contact information	Sedigheh Farzi: sedighehfarzi@nm.mui.ac.ir
Notes

**Table:** IRCT20200613047757N1

Study name	Evaluation of the effectiveness of mobile‐based inflammatory bowel disease management system by using gamification techniques on disease activity index, mental health and quality of life
Methods	RCT
Participants	Target sample size: 210
Interventions	Intervention group: will receive a mobile‐based IBD management system using playfulness techniques in addition to standard care. Control group: will receive standard and routine outpatient clinics based on guidelines.
Outcomes	QOL, disease activity, stress, depression
Starting date	22 November 2022
Contact information	Narges Norouzkhani: narges.norouzkhani@yahoo.com
Notes

**Table:** Kim 2020

Study name	A cluster‐randomised controlled trial of a decision aid (myAID) for ulcerative colitis patients to enhance patients quality of life, empowerment, quality of decision making and disease control
Methods	Cluster RCT
Participants	460
Interventions	IG: myAID is an internet‐based multimedia decision aid (Emmi Solutions, Chicago) designed to support shared decision making for treatment choices in UC management CG: usual care without the use of the decision aid. Patients will be reviewed by their respective gastroenterologist (or team) within 2‐4 weeks of study inclusion to help with the decision making process.
Outcomes	Primary outcome: HRQoL Secondary outcomes: Empowerment Health literacy Quality of decision making Anxiety Productivity Medication adherence Disease activity and control, as measured by: Simple Clinical Colitis Activity IndexTime point (8)12 months faecal calprotectin proportion of patients taking steroids (captured through self‐report via online questionnaire) proportion of patients requiring surgery (captured through self‐report via online questionnaire) proportion of patients requiring unplanned hospital admissions (captured through self‐report via online questionnaire) Acceptability of the decision aid, as measured by questions specifically developed for the study (intervention arm only)
Starting date	25 August 2018
Contact information	Dr Andrew Kim: andrew.h.kim@student.unsw.edu.au
Notes	Author responded on 16 June 2021 their study is not yet complete although has now finished recruitment. Full data analysis is therefore not available as yet.

**Table:** NCT03827109

Study name	Peer mentoring to improve self‐management in youth with IBD
Methods	RCT
Participants	300
Interventions	IG: peer mentoring programme for improving the self‐management of youth with IBD CG: "Educational Activity" comparison
Outcomes	The primary outcomes are youth QOL and functioning in typical life activities. Secondary outcomes are disease outcomes, including disease severity and clinical outcomes (hospital admissions, clinic appointments, missed appointments, procedures). Mentor and parent QOL will also be assessed as secondary outcomes
Starting date	1 February 2019
Contact information	Dr Laura Mackner: Laura.Mackner@nationwidechildrens.org>
Notes	Per the authors, due to COVID this trial was extended and the extension would get them through 2024, but data analysis might continue into 2025.

**Table:** RBR‐79dn4k

Study name	Impact of an intervention program on nonadherence to drugs in ulcerative colitis patients: randomised clinical trial
Methods	RCT
Participants	90
Interventions	IG: video of approximately 5 minutes duration will be presented presenting basic contents on IBD and importance of adherence to the prescribed drug treatment, made by the research team. The video is available on following link: https://youtu.be/vcvm9DXAXNg. Educational booklet will be presented presenting basic contents on the UC and the importance of adherence to the prescribed drug treatment, prepared by the research team based on the available scientific evidence. The subject will have 10 minutes for silent reading, and at the end, the team will be available to answer questions from the participants. The verbal guidelines will be using as reference the drug monographs available in the Micromedex® database. Behavioral interventions: ‐ a therapeutic scheme shall be drawn up. Participants who demonstrate difficulty in understanding the written guidelines shall additionally receive a Pharmaceutical Guidance in order to facilitate the understanding of schedules and quantities of each prescribed medication. Short Message Service (SMS) messages will be sent to the cell phones registered by the participants: ‐ Reminder messages of the date of care for procurement of medicines. This intervention will be carried out at least 2 days before the date of return and 2 hours before the scheduled time; ‐ messages of the motivational type with the contents exemplified. This intervention will be carried out once a week. CG: participants will not receive the above
Outcomes	Medication adherence QOL Clinical status
Starting date	10 January 2019
Contact information	Genoile Oliveira Santana: milabahia@yahoo.com.br
Notes	The authors responded they are submitting the complete work for publication and it will be soon fully available.

**CG:** control group**; HRQoL:** health‐related quality of life; **IBD:** inflammatory bowel disease; **IG:** Intervention group**; QOL:** quality of life; **RCT:** randomised controlled trial; **UC:** ulcerative colitis

## Differences between protocol and review

The Types of interventions section has been clarified. This further explains the inclusion and exclusion characteristics used. This now makes the distinction between education that allows another separate intervention, such as education on psychosocial therapies, education on remote monitoring tools or education on diagnostic tools and clarifies these are excluded. All such interventions are covered in alternative reviews in the IBD portfolio from this Cochrane group.

When educational content focused on IBD itself, specific knowledge on the disease, symptoms, causes, management, side effects, direct skills to enhance outcomes and medication use and or adherence, studies were included.

The search methods for the protocol were not peer‐reviewed. After the publication of the protocol and before running the searches, we asked an independent Cochrane Information Specialist to peer‐review, revise, and run the searches. Based on his comments, we removed manual scanning for conference abstracts because all major relevant conferences are now indexed in Embase and CENTRAL. We also removed scanning the Internet from the method as an unclear description. In addition, peer‐review comments suggested that three of the databases were irrelevant to the topic of this review and we could remove them if we had limited resources to conduct searches. As a result, and since we had limited sources, we removed them.

Another difference from the protocol is the clarification about standard therapies and related terms as used in the included studies. We consider it highly unlikely that standard therapies were replaced by any of the interventions studied in this review, and we have assumed that they were offered for all study groups whether or not that was mentioned by the study authors. We have also assumed that all standard therapies were similar enough to be categorised together for the purposes of our meta‐analyses.

Due to a lack of data we did not perform the subgroup and sensitivity analyses we had planned, apart from the sensitivity analysis for the cluster‐RCTs. For the same reason we did not perform the funnel plot analysis to check for publication bias.

We changed the requirement for a sensitivity analysis for risk of bias in our methods section from any high or unclear judgements for allocation or performance bias to high or unclear judgements in any field except performance bias, as all studies were open‐label and thus performance bias was high for all of them.

We clarified our plans for dealing with missing data. These did not change, but were not clear in the protocol and as the editorial management of the review shifted with time, it became important to clarify them. For negative outcomes we used the plausible worst‐case scenario and added the numbers of dropouts to the numerator, as is normal practice for reviews for IBD, given the chronic nature of the condition and the high rates of adverse events and treatment failures across a patient's journey. For withdrawals due to adverse events specifically, we considered as adverse events all unspecified reasons and all reasons that did not automatically preclude the possibility of an adverse event. For analyses using continuous outcomes, we used the sample numbers as reported by the authors, for each particular continuous outcome. If the sample numbers were not reported, we estimated the sample number based on the attrition percentages reported. For cluster‐trial data we estimated effective sample sizes based on chapter 23 of the *Cochrane Handbook for Systematic Reviews of Interventions* (Higgins 2020).

We also did not perform the planned qualitative analysis for patient knowledge, or skill, and knowledge assessments that we had planned in the protocol, due to lack of data.

We initially planned sensitivity analysis on just one primary outcome. However, as all primary outcomes are critical we revised this plan to all of them.

Finally, peer review feedback from another review we were conducting highlighted that a sensitivity analysis for quality of life measures in which unvalidated measures were removed would be of value and so we amended our plan in this review.
